# Causal effect of gut microbiota on juvenile idiopathic arthritis: A two‐sample Mendelian a randomization study

**DOI:** 10.1111/jcmm.70183

**Published:** 2024-10-30

**Authors:** Lian Zhang, Zhihua Yang, LuLu Zhang, Yanwen Wei, Lisheng Wan

**Affiliations:** ^1^ Shenzhen Children's Hospital Shenzhen China; ^2^ Department of Internal Medicine V Hematology Oncology Rheumatology Heidelberg University Hospital Heidelberg Germany; ^3^ Shenzhen Hospital of Integrated Traditional Chinese and Wsestern Medicine Shenzhen China

**Keywords:** gut microbiome, juvenile idiopathic arthritis, two‐sample Mendelian randomization

## Abstract

There is increasing evidence of a significant association between the gut microbiome and juvenile idiopathic arthritis (JIA). However, whether this association is causal remains to be determined. This study was a two‐sample Mendelian randomization (MR) study using publicly available genome‐wide association study (GWAS) summary data to investigate the causal relationship between the gut microbiome and JIA. We used summary data on gut flora and JIA obtained from genome‐wide association studies (GWAS) from MiBioGen and NHGRI‐EBI, using inverse variance weighting as the main method to analyse causality in the TSMR causality analysis. To check the stability of the TSMR results, we performed several sensitivity analyses and assessed the presence of reverse causality through a reverse TSMR analysis. We calculated the degree of sample overlap where applicable. The current TSMR analyses identified four bacterial taxa associated with JIA. Specifically, two bacteria, Catenibacterium (*p* = 2 × 10–2) and Holdemania (*p* = 4 × 10–2), were negatively associated with the risk of developing JIA, suggesting a protective effect, while Olsenella (*p* = 1 × 10–2) and Rikenellaceae (RC9gutgroup) (*p* = 1 × 10–2) were positively associated with the risk of JIA, suggesting that these two bacteria may be risk factors for JIA. However, the results for Catenibacterium and Holdemania should be interpreted with caution due to instability observed in ‘leave‐one‐out’ sensitivity analyses. Reverse TSMR analyses found no evidence of reverse causality between JIA and gut flora. Our confirmation of a causal relationship between gut flora and JIA provides an innovative perspective for the study of JIA: targeting and modulating dysregulation of specific bacterial taxa to prevent and treat JIA.

## 1 INTRODUCTION

Juvenile idiopathic arthritis (JIA), the most common chronic rheumatic disease of childhood, is not a single disease entity but a group of genetically heterogeneous and phenotypically distinct disorders that affect not only joints but also extra‐articular structures, including the skin, eyes and internal organs, leading to disability and even death in severe cases. It has an annual incidence of 2–20 cases per 100,000 population and a prevalence of 16–150 cases per 100,000 in high‐income countries.^1^ JIA is defined as monoarthritis or polyarthritis of at least 6 weeks duration, beginning before the age of 16 years and excluding other known aetiologies.^2^ JIA is an autoimmune disease with a complex aetiology, whose pathogenesis is not yet fully understood and involves a variety of mechanisms. It's currently thought that the main factors in the development of JIA are genetic and environmental.^3^ The treatment of JIA inevitably involves the systemic application of high‐dose glucocorticosteroids, which, although effective in the short term, produce serious side effects in the long time, including osteoporosis, growth inhibition, immunosuppression, and other adverse prognosis.^4^ It is, therefore, imperative to understand the aetiology of JIA to facilitate the development of therapeutic strategies with low or no side effects.

Recently, the causal relationship between the composition of the gut microbiome and the risk of JIA has received much attention. The microbiome is the genomes of bacteria, archaebacteria, fungi and viruses that inhabit our gut, skin, mouth and other body sites. Collectively, these microbial genes far outnumber human genes and perform critical functions that help maintain homeostasis in the body, not only in the microbes' respective environments but also in a wide range of human hosts.^5^ Recent studies have found close interactions between the gut microbiome and human immune cells, suggesting that the gut microbiome plays a vital role in regulating the immune system.^6^ A case–control study of the gut microbiome in children with JIA described differences in the composition of the faecal microbiota between children with JIA and unaffected children and found that the microbial diversity was reduced in children with JIA, leading them to think that alterations in the composition of the faecal microbiota may contribute to the development of subclinical intestinal inflammation and promote inflammation in the joints.^7^ A study suggests that alterations in gut microflora may contribute to the development of JIA by challenging the mucosal immune system in genetically susceptible individuals predisposed to a local pro‐inflammatory cascade.^8^ Although the pathogenesis of JIA is not fully understood, there is increasing evidence that the gut microbiome may play an essential role in the development and progression of JIA. However, it is unclear whether there is a causal relationship between the gut microbiome and JIA.

In this context, mendelian randomization (MR) is a novel approach to exploring causal associations between gut microbiota and JIA. MR is a method for integrating summary data from genome‐wide association studies (GWAS) that minimizes confounding factors, such as environmental factors. MR is widely used to infer the presence or absence of causal associations and to evaluate associations between exposures and complex outcomes. It uses genetic variants significantly associated with exposure as instrumental variables (IV) to infer causality.^9^ If exposure has a causal effect on the outcome, then IVs that affect exposure will proportionally affect the outcome. In the current study, we used GWAS summary statistics from MiBioGen and NHGRI‐EBI to perform a two‐sample MR analysis to explore whether there is a causal relationship between gut microbiome composition and JIA risk. Where relevant, we also examined and calculated the degree of sample overlap between the exposures and outcomes.

## 2 MATERIALS AND METHODS

### 2.1 Study design

This study used two‐sample MR analysis to assess the causal relationship between gut flora and JIA. We obtained summary‐level data on gut flora and JIA from the Genome‐Wide Association Study (GWAS) and preliminarily screened isolated gut microbiota (exposure factors) significantly associated with JIA for detailed MR analysis. In addition, to obtain reliable results, the MR analysis met the following three assumptions^10^:(1) the IVs ultimately included for use must be strongly correlated with the exposure factor; (2) the included IVs and confounders are independent of each other; and (3) there is no horizontal pleiotropy: the IV affects the outcome only through the exposure, implying that there is no horizontal pleiotropic effect between the IV and the outcome. Where applicable, we also calculated the degree of sample overlap between the exposures and outcomes and reported the implications of any overlap in the results.

### 2.2 Data sources

We chose single nucleotide polymorphisms (SNPs) associated with the composition of the human gut microbiome as IVs in a large‐scale, multi‐ethnic GWAS to explore associations between human autosomal genetic variants and the gut microbiome. The study included 24 cohorts of European, Hispanic, Middle Eastern, Asian and African ancestry, comprising a total of 18,340 individuals, with the European cohort being the largest (*n* = 13,266). The study used seven different faecal DNA extraction methods and analysed microbial composition by targeting three other variable regions of the 16S rRNA gene (V4, V3–V4, V1–V2). We then used a direct taxonomic hierarchy to classify the genera. In this study, genus level was considered the lowest taxonomic level, and a total of 131 genera with mean abundance more significant than 1% were identified, including 12 unknown genera. Finally, 119 genus‐level taxa were included in this study for further analysis.^11^ The screening threshold for each type of intestinal flora in this article was *p* < 1.0 × 10^−5^.^12^ GWAS summary statistics for JIA are available from the published GWAS of JIA (*n* = 31, 142 European individuals) (https://www.ebi.ac.uk/gwas/).^13^ This GWAS included 6056 cases and 25,086 controls of European origin. Detailed characteristics of the study population were described in the original study.

### 2.3 IV (Instrument variables) selection

To ensure the integrity and accuracy of our conclusions regarding the causal relationship between gut microbiota and JIA risk, we used the following quality control steps in selecting the optimal IVs:(1) Correlations between the IVs and the gut microbiota were assessed using a threshold of *p* < 1.0 × 10–5^12^;(2) In this study, a clustering process was performed (*R* ^2^ < 0.001 and cluster distance = 10,000 kb) to assess linkage disequilibrium (LD) between the included SNPs; (3) To avoid allelic effects on the causal relationship between gut microbial (GM) taxa and JIA, we removed palindromic SNPs.

### 2.4 Statistical analysis

In this study, we chose a variety of methods including inverse variance weighted (IVW), MR‐Egger, weighted median and weighted mode to examine whether there is a causal relationship between gut microbiota and JIA. IVW is essentially a meta‐analytic approach that uses weighted regression to transform the results of IVs on exposure effects to obtain an overall estimate of the effect of the gut microbiome on the risk of JIA, with the intercept restricted to zero.^14^ When horizontal pleiotropy was absent, we used the IVW method to avoid the effects of confounders and thus obtain unbiased estimates. OR and 95% confidence intervals (CIs) indicate effect sizes. MR‐Egger regressions were performed based on the assumption that the instrument strength is independent of the direct effect (InSIDE), which allows using an intercept term to assess the presence of pleiotropy. The intercept term is equal to zero if the instrument strength is equal to the direct effect, and the intercept term is equal to zero if the instrument strength is equal to the immediate impact. If the intercept term is equal to zero, it indicates the absence of horizontal pleiotropy, and the results of the MR‐Egger regression are consistent with IVW.^15^ However, MR‐Egger can be strongly influenced by outlying genetic variables, leading to inaccurate estimates. Despite some invalid IVs, the MR‐Egger method can still provide unbiased estimates and can be used to detect the presence of genetic correlation bias. Weighted medians can provide consistent appraisals of causal effects even when up to 50% of the information in the analysis comes from invalid IVs.^16^ The weighted modal approach is similar to the weighted median method, but unlike the weighted median method, the weighted modal process can deal with the case of multiple multinomials. It applies more to circumstances with several valid IVs with similar causal estimates. The weighted modal approach remains valid even when other IVs do not meet the requirements of the MR method for causal inference.^17^ People often use Cochrane's *Q* test to test for heterogeneity, where an IV of *p* < 0.05 indicates the presence of heterogeneity. People often use the intercept of the MR Egger regression to assess potential pleiotropy in IV. If *p* > 0.05, horizontal pleiotropy is not to be considered. To check the reliability and stability of the causal effect estimates, we used leave‐one‐out sensitivity analyses to determine the presence of potentially strong effect SNPs. The results for Catenibacterium and Holdemania were found to be unstable in these analyses, indicating the need for cautious interpretation. To ensure the accuracy of the causality results (based on the IVW results) between GM taxa and JIA, we performed further pluripotency analyses using the MR‐PRESSO test (using the R package ‘MRPRESSO’) and excluded possible outliers. To assess the causal relationship between gut microbiota and JIA, we also performed reverse MR analysis and identified bacteria causally associated with JIA in forward MR analysis. The methodology and setup of the reverse MR analysis were consistent with that of the forward MR.

We calculated the F statistic using the formula *F* = *R* ^2^ × (*N*‐1‐*K*)/(1‐*R* ^2^) × *K*, where *R* ^2^ is the proportion of exposure variance explained by genetic variation, *N* is the sample size, and *K* is the number of instruments. No significant weak instrumental bias was considered if the corresponding *F* statistic was >10.^18^ We performed statistical analyses using R software (version 4.3.1, TwoSampleMR package).

## 3 RESULTS

### 3.1 Main results of the 119 bacterial traits with the risk of JIA

A total of 1548 SNPs were used as IVs for 119 bacterial genera based on the screening criteria for IVs. For more information on the selected IVs, see Additional file 1: Table S1. Briefly, we observed a profile of suggestive evidence for five bacterial traits associated with JIA risk using the IVW approach, as detailed in Table 1.

**TABLE 1:** MR estimates for the association between gut microbiota and JIA.

Bacterial taxa (exposure)	*N*. snp	MR method	Beta	SE	OR (95% CI)	*p*‐value	*F*‐statistic	Horizontal pleiotropy			Heterogeneity	
								Egger intercept	SE	*p*‐value	Cochran's *Q*	*p*‐value
Catenibacterium	6	MR‐Egger	‐0.90	0.97	0.40 (0.06‐2.71)	0.40	65.95	0.09	0.13	0.53	4.27	0.37
	6	IVW	‐0.25	0.10	0.78 (0.64–0.96)	0.02						
	6	Weighted median	‐0.20	0.14	0.82 (0.62‐1.08)	0.15						
	6	Simple mode	‐0.41	0.23	0.67 (0.42‐1.05)	0.14						
	6	Weighted mode	‐0.15	0.23	0.86 (0.55‐1.35)	0.54						
Holdemania	20	MR‐Egger	‐0.29	0.28	0.75 (0.43‐1.29)	0.31	62.45	0.01	0.03	0.69	15.9	0.60
	20	IVW	‐0.19	0.09	0.83 (0.69–0.99)	0.04						
	20	Weighted median	‐0.12	0.14	0.88 (0.68‐1.16)	0.37						
	20	Simple mode	‐0.04	0.25	0.96 (0.59‐1.58)	0.88						
	20	Weighted mode	‐0.04	0.27	0.96 (0.57‐1.62)	0.89						
Intestinimonas	65	MR‐Egger	0.41	0.28	1.50 (0.87‐2.59)	0.15	41.66	‐0.05	0.02	0.03*	88.1	0.02
	65	IVW	‐0.20	0.07	0.82 (0.71–0.94)	0.01						
	65	Weighted median	‐0.61	0.09	0.54 (0.46–0.64)	<0.01						
	65	Simple mode	‐0.63	0.19	0.53 (0.37–0.78)	<0.01						
	65	Weighted mode	‐0.62	0.20	0.54 (0.36–0.80)	<0.01						
Olsenella	13	MR‐Egger	‐0.18	0.30	0.84 (0.47‐1.50)	0.57	66.79	0.05	0.04	0.21	11.2	0.43
	13	IVW	0.20	0.08	1.23 (1.05–1.44)	0.01						
	13	Weighted median	0.17	0.11	1.19 (0.95‐1.49)	0.12						
	13	Simple mode	0.17	0.20	0.57 (1.19‐1.77)	0.41						
	13	Weighted mode	0.17	0.19	1.18 (0.82‐1.71)	0.40						
Rikenellaceae (RC9gutgroup)	13	MR‐Egger	0.18	0.45	1.19 (0.50‐2.86)	0.70	45.76	<0.01	0.06	0.98	4.66	0.95
	13	IVW	0.19	0.08	1.21 (1.04–1.40)	0.01						
	13	Weighted median	0.21	0.10	1.23 (1.01–1.51)	0.04						
	13	Simple mode	0.29	0.16	1.33 (0.98‐1.82)	0.09						
	13	Weighted mode	0.27	0.16	1.31 (0.96‐1.78)	0.11						

**FIGURE 1:**
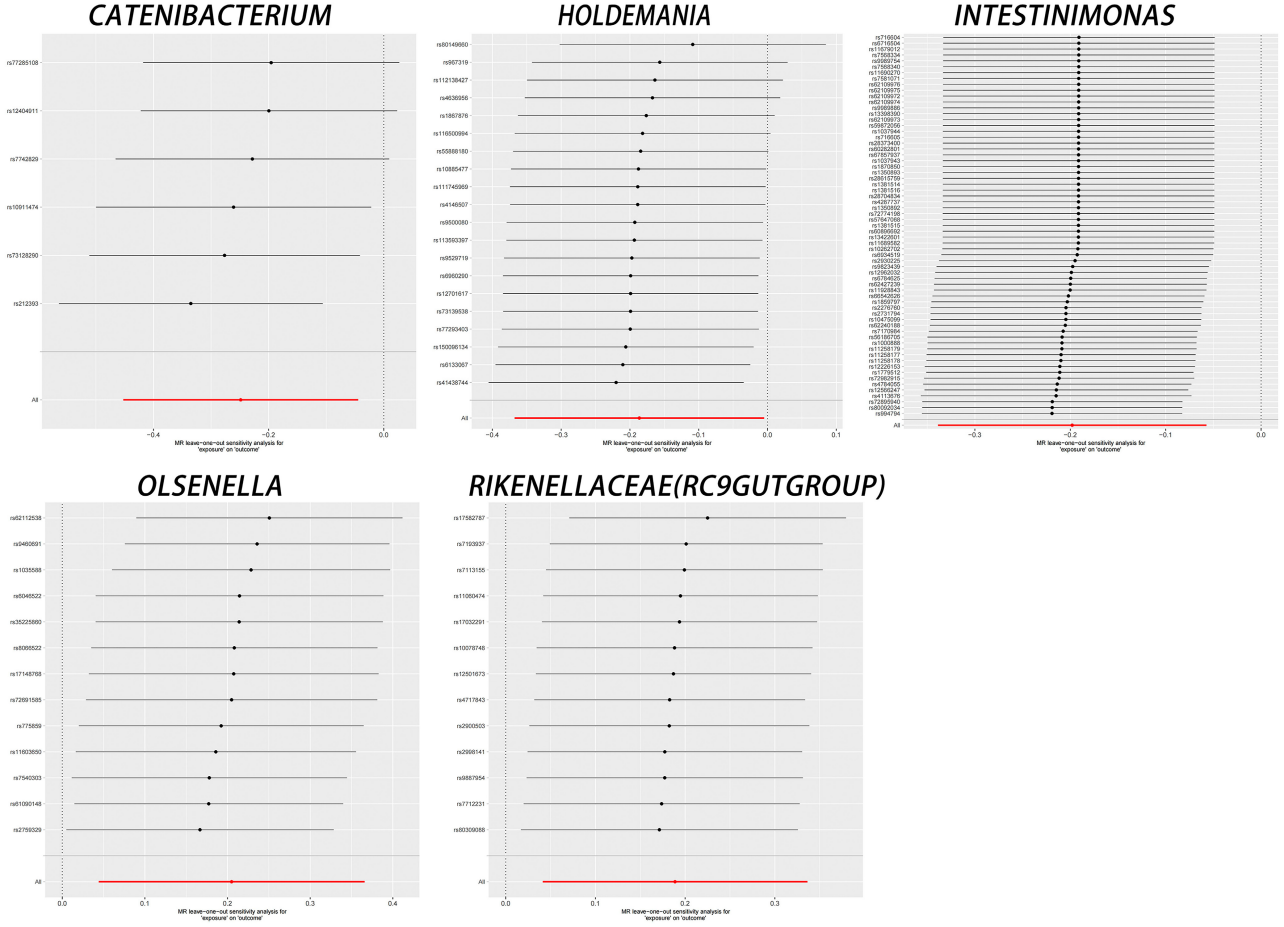
Leave‐one‐out plots for the causal association between gut microbiota and JIA. This figure displays the results of leave‐one‐out sensitivity analysis, which tests the stability of the causal association for each gut microbiota with JIA. For each bacteria: Catenibacterium, Holdemania, Intestinimonas, Olsenella and Rikenellaceae (RC9gutgroup), one SNP is removed at a time to assess the robustness of the results.

We found that in the IVW approach, the results indicated that Catenibacterium was negatively associated with the risk of developing JIA, suggesting a protective effect (OR = 0.78, 95% CI: 0.64–0.96, *p* = 2 × 10–2). The intercept of the MR‐Egger regression showed no potential horizontal pleiotropy (intercept *p*‐value = 0.09). The results of the Cochran IVW *Q*‐test (*Q*_*p*‐value = 0.37) showed no significant heterogeneity in these IVs. Holdemania's IVW estimates also showed a negative association with the risk of JIA prevalence, suggesting a protective effect (OR = 0.83, 95% CI: 0.69–0.99, *p* = 4 × 10–2). The intercept of the MR‐Egger regression showed no potential for horizontal pleiotropy (*p*‐value of the intercept = 0.01). The results of the Cochran IVW *Q*‐test (*Q*_*p*‐value = 0.60) showed no significant heterogeneity in these IVs. However, it is important to note that the results for Catenibacterium and Holdemania were found to be unstable in the ‘leave‐one‐out’ sensitivity analysis (Figure 1), indicating that the protective effect observed should be interpreted with caution. Although the IVW method suggested a protective effect of Intestinimonas against JIA (OR = 0.82, 95% CI: 0.71–0.94, *p* = 1 × 10–2), weighted median (OR = 0.54, 95% CI: 0.46–0. 64, *p* < 1 × 10 −2), simple mode (OR = 0.53, 95% CI: 0.37–0.78, *p* < 1 × 10–2), weighted mode (OR = 0.54, 95% CI: 0.36–0.80, *p* < 1 × 10–2) also showed its suggestive protective effect against JIA. However, MR‐Egger regression showed (intercept *p*‐value = −0.05, *p*‐value <0.05) suggesting a horizontal pleiotropy between gut flora and JIA, which did not fulfil the three main hypotheses of MR and was therefore excluded. The IVW estimates for Olsenella showed a positive association with the risk of developing JIA, suggesting that Olsenella may be a risk factor for JIA (OR = 1.23, 95% CI: 1.05–1.44, *p* = 1 × 10–2), and the intercepts of the MR‐Egger regression showed no potential horizontal pleiotropy (intercepts *p*‐value = 0.05). The results of the Cochran IVW *Q*‐test (*Q*_*p*‐value = 0.43) showed no significant heterogeneity in these IVs. The IVW estimate of Rikenellaceae(RC9gutgroup) also showed a positive association with the risk of developing JIA, suggesting that Rikenellaceae(RC9gutgroup) may be a risk factor for JIA (OR = 1.21, 95% CI: 1.04–1.40, *p* = 1 × 10 −2), and the association between Rikenellaceae(RC9gutgroup) and JIA remained stable in the weighted median method (OR: 1.23; 95% CI: 1.01–1.51; *p* = 4 × 10–2). MR‐Egger regression of the intercepts showed no potential for horizontal pleiotropy (intercept p‐value <0.01) and the results of the Cochran IVW *Q*‐test (*Q*_*p*‐value = 0.95) showed no significant heterogeneity in these IVs.

**FIGURE 2:**
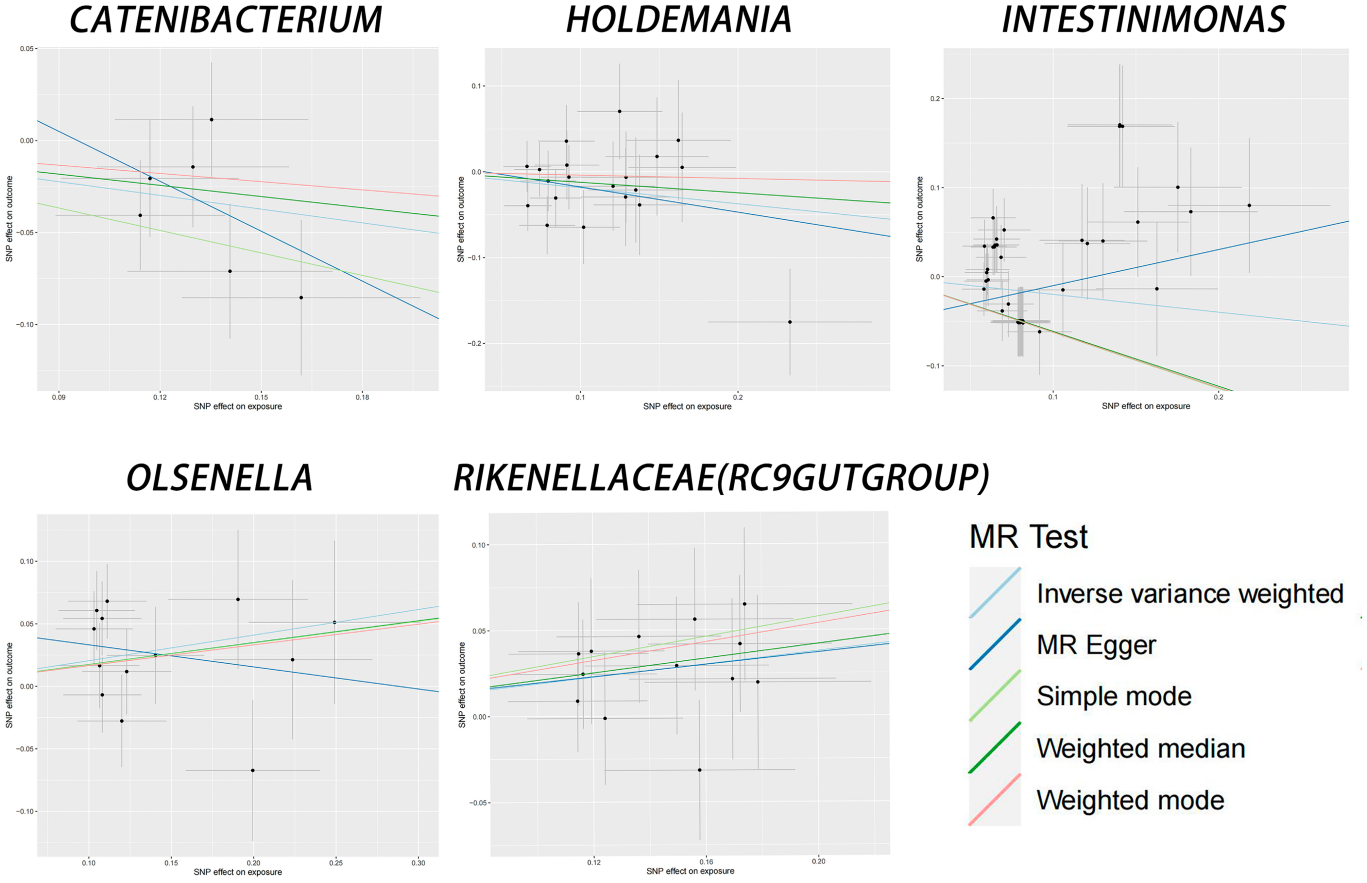
Scatter plots for the causal association between gut microbiota and JIA. This figure shows the scatter plots for the causal effect of five gut microbiota on JIA risk using different MR methods. The methods used are Inverse variance weighted, MR‐Egger, Simple mode, Weighted median and Weighted mode, applied to Catenibacterium, Holdemania, Intestinimonas, Olsenella and Rikenellaceae (RC9gutgroup), respectively.

**FIGURE 3:**
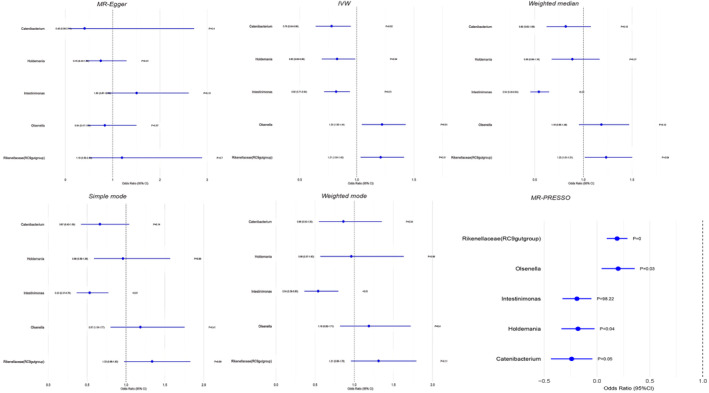
Forest plots for the causal association between gut microbiota and JIA. This figure shows forest plots of the causal association between gut microbiota and JIA based on various MR methods. Methods used include MR‐Egger, Inverse variance weighted (IVW), Weighted median, Simple mode, Weighted mode and MR‐PRESSO, applied to Catenibacterium, Holdemania, Intestinimonas, Olsenella and Rikenellaceae (RC9gutgroup), respectively.

In addition, horizontal pleiotropy between Intestinimonas and JIA was excluded based on the results of MR‐Egger regression intercept analysis. For the remaining four causal relationships, the range of F‐statistics for the IVs was 45.76–66.79, which eliminated the bias of weak IVs. The results of the Cochran IVW *Q*‐test showed that there was no significant heterogeneity in these IVs. There was no significant directional level pleiotropy. In the scatterplot, potential outliers (Figure 2) and leave‐one‐out plots (Figure 1) were present for Holdemania, Intestinimonas and Olsenella IVs by visual inspection. Further MR‐PRESSO analysis revealed significant outliers for Intestinimonas (overall test *p* = 98.22), and MR‐PRESSO analysis for the remaining four colonies revealed no significant outliers (overall test *p* > 0.05). (as detailed in Table 2). A ‘forest plot’ was constructed by R software to visualize the causal relationship between gut microbiota and JIA (Figure 3). Thus, the evidence again suggests the existence of pleiotropy at the level of association between Intestinimonas and JIA. However, there was insufficient evidence for pleiotropy at the association level between the remaining four bacteria and JIA.

**TABLE 2:** MR‐PRESSO analysis for the association between gut microbiota and JIA.

Bacterial taxa (exposure)	MR Analysis	Causal Estimate	SD	*T*	*p*‐value	RSSobs	Global test *p*‐value	Remove SNP
Catenibacterium	MR‐PRESSO	‐0.24	0.10	‐2.44	0.05	7.13	0.47	/
Holdemania	MR‐PRESSO	‐0.18	0.08	‐2.18	0.04	18.53	0.62	/
Intestinimonas	MR‐PRESSO	‐0.19	0.07	‐2.76	98.22	0.01	>0.05	/
Olsenella	MR‐PRESSO	0.20	0.08	2.50	0.03	15.23	0.40	/
Rikenellaceae (RC9gutgroup)	MR‐PRESSO	0.19	0.05	4.03	0.00	5.51	0.97	/

### 3.2 The result of reverse MR analysis

We assessed potential inverse associations of the four bacterial traits with JIA using inverse MR analysis. Using the IVW method, we found no statistically significant associations between JIA and any of these four bacterial traits (OR: 1.03; 95% CI: 0.97,1.09; *p* = 0.311 for Catenibacterium; OR: 0. 99; 95% CI: 0.97, 1.02; *p* = 0.588 for Holdemania; OR: 1.00; 95% CI: 0.95, 1.07; *p* = 0.801 for Olsenella; OR: 1.02; 95% CI: 0.97, 1.06; *p* = 0.496 for RikenellaceaeRC9gutgroup).

## 4 DISCUSSION

JIA is a group of chronic arthritis with childhood onset. The pathogenesis of the disease is now mostly considered to be multifactorial. For the microbiota, the influencing factors may be the early use of antibiotics, early weaning breastfeeding and caesarean sections.^19^, ^20^, ^21^ Recent hot topics of discussion are the gut microbiota and its interaction with the mucosal and gastrointestinal immune systems, which may contribute to disease progression in JIA.^22^, ^23^

The trillions of commensal gut microbiomes involved in the regulation and maintenance of the host immune system colonize the mucosal surface of the human gastrointestinal tract in large numbers. Thus, ecological dysregulation of the gut microbiome interacts closely with the gut mucosal immune system.^24^ The study found altered gut flora in patients with JIA, characterized by a reduction in the abundance of short‐chain fatty acids (SCFAs) produced by four gut flora species, with the decrease in the four species suggesting more severe clinical indicators.^25^ SCFA, including acetic, propionic, butyric and valeric acids, may exert a more pronounced immunomodulatory effect through several pathways, such as inducing the differentiation of regulatory T cells, enhancing IL‐10 production and inhibiting Th 17 cells.^26^, ^27^, ^28^ In addition, butyrate‐supplemented mice suppressed the expression of inflammatory cytokines via the Treg/IL‐10/Th17 axis, and ameliorated collagen‐induced arthritis in the mice.^27^ However, the role of gut microbiota and SCFAs in JIA remains unclear and requires further research. Several studies have found an altered gut microbiome in JIA, but no single microorganism or microbiota is associated with JIA. Changes in the faecal microbiome of children with new‐onset JIA compared to healthy children.^8^ Differences in microbial diversity and classification suggest that gut microbes may be involved in the pathogenesis of early JIA.

Study Finds Lower Levels of Catenibacterium in Gut Flora Associated with Low Adherence to Mediterranean Diet (MD).^29^ A large body of evidence suggests that MD is beneficial to human health and is associated with reduced markers of inflammation. Catenibacterium, as a producer of SCFAs, plays a crucial role in intestinal homeostasis and is thought to benefit host health.^30^, ^31^ We also found that the level of Slfn1, a late LPS‐responsive gene in mouse macrophages, mRNA in the colon was positively correlated with the relative abundance of Catenibacterium, suggesting a potential regulatory interaction between Catenibacterium and Slfn1.^32^ Holdemania is involved in butyrate production, and Olsenella has been reported as an SCFA‐producing bacterium^33^; butyrate affects gut physiology and the immune system and has been linked to the activation of Treg cell differentiation in the gut through histone acetylation,^26^ which plays a role in maintaining gut homeostasis and resistance to inflammation.^34^

In contrast, another study found that an increase in Olsenella was associated with increased inflammation; when inflammatory hyperthermia leads to disruption of the host gut microbiota, proliferation of the low abundance pathogenic bacterium Olsenella leads to further worsening of the inflammatory response.^35^ In contrast, the down‐regulation of Olsenella reduces the likelihood of rumen epithelial and organismal inflammation.^36^ The HLA‐B27 gene is a significant risk factor for several clinical diseases, including JIA. Still, the mechanisms of increased risk are not fully understood, and HLA‐B27 positivity is strongly correlated with the ERA subtype of JIA. One study found that HLA‐B27‐positive rats had a reduced relative abundance of Rikenellaceae (RC9gutgroup) in the gut compared to wild‐type rats.^37^ Rikenellaceae (RC9gutgroup) was positively associated with ‘harmful indicators’ and negatively associated with ‘beneficial indicators’ induced by a high‐fat diet.^38^

This is the first bidirectional TSMR study to demonstrate a causal relationship between gut microbiota and JIA that is not confounded by confounders or reverse causality. Four bacterial taxa associated with JIA were identified using TSMR analysis. These identified key bacterial taxa may be candidates for microbiome interventions in future JIA clinical trials. Additionally, we suggest that further exploration of the ‘gut‐bone axis’ in future studies may help uncover the mechanisms by which gut microbiota influence bone metabolism, offering new therapeutic targets for JIA. In the meantime, our findings may provide an innovative perspective for JIA research: targeting and modulating specific bacterial taxa, such as supplementing beneficial bacteria and inhibiting the growth of harmful bacteria, for the prevention and treatment of JIA. Although our study analysed the typical gut microbiota, the gut microbiota is large in number, and therefore, our study data may still need to be expanded. Further subgroup analyses were not possible due to the lack of demographic data (e.g. gender and ethnicity) in the original study. In addition, our study only analysed populations from the European region, which means that the extension of the findings to other populations must be carefully considered. In conclusion, our results suggest that specific gut microbiota may reduce the risk of JIA and that attention needs to be paid to the particular mechanisms of these effects of the gut microbiota and to elucidate the complex interactions with the gut microbiota.

## 5 CONCLUSION

Our results showed that Catenibacterium and Holdemania reduced the risk of JIA and had a protective effect, whereas Olsenella and Rikenellaceae (RC9gutgroup) increased the risk of JIA. However, due to the instability observed in sensitivity analyses, particularly for Catenibacterium and Holdemania, these findings should be interpreted with caution. Further research is needed to investigate the protective effects and specific mechanisms by which these gut microbiota influence JIA.

## AUTHOR CONTRIBUTIONS

**Lian Zhang:** Data curation (equal); methodology (equal); resources (equal); writing – original draft (equal). **Yanwen Wei:** Visualization (equal); writing – original draft (equal). **Lisheng Wan:** Funding acquisition (equal); writing – review and editing (equal). **LuLu Zhang:** Writing – review and editing (equal). **Zhihua Yang:** Writing – review and editing (equal).

## FUNDING INFORMATION

This study was supported by Basic Research of Shenzhen Science and Technology Innovation Commission (top‐level project): Study on the mechanism of action of Dispelling Wind and Remitting Drink Soup based on lipid metabolism pathway in intervening bronchial asthma in children with cold croup (Project No. JCYJ20210324120809026) and Guangdong Provincial Bureau of Traditional Chinese Medicine: Study on the Effect of Dispelling Wind Remitting Drinking Soup on Lipid Metabolism in Expiratory Condensate of Children with Cold Croup Bronchial Asthma (Project No. 20221341).

## CONFLICT OF INTEREST STATEMENT

No conflicts of interest to disclose. The authors have no financial relationships relevant to this article to disclose.

## Supporting information


Table S1.

## Data Availability

The datasets generated and analysed during the current study are available from the corresponding author on reasonable request.
