# Supplementary material for: Causal effect of gut microbiota on juvenile idiopathic arthritis: A two‐sample Mendelian a randomization study
Source: J Cell Mol Med. 2024 Oct 30;28(20):e70183. doi: 10.1111/jcmm.70183 (PMC11522359; doi:10.1111/jcmm.70183)
Supplement: Supplementary file 1 — Table S1. [file JCMM-28-e70183-s001.docx]

| **Table S1 Instrumental variables used in MR analysis of the association between gut microbiota and JIA.** | | | | | | | | | |  |
| --- | --- | --- | --- | --- | --- | --- | --- | --- | --- | --- |
| **Bacterial taxa (exposure)** | **SNP** | **Effect allele** | **Other allele** | **Exposure (Bacteria)** | | | **Outcome (PE)** | | |  |
|  |  |  |  | **Beta** | **SE** | **P-value** | **Beta** | **SE** | **P-value** | |
| *Actinomyces* | rs10787984 | G | C | 0.094 | 0.021 | 9.99E-06 | 0.012 | 0.037 | 0.75 | |
| *Actinomyces* | rs16920436 | A | G | 0.086 | 0.019 | 8.73E-06 | -0.003 | 0.034 | 0.92 | |
| *Actinomyces* | rs2715439 | C | T | 0.075 | 0.016 | 5.89E-06 | -0.039 | 0.030 | 0.19 | |
| *Actinomyces* | rs34583783 | G | T | 0.127 | 0.027 | 2.41E-06 | -0.032 | 0.048 | 0.51 | |
| *Actinomyces* | rs35011108 | A | G | 0.233 | 0.051 | 5.54E-06 | 0.034 | 0.073 | 0.65 | |
| *Actinomyces* | rs4073240 | G | A | 0.075 | 0.017 | 7.49E-06 | 0.006 | 0.030 | 0.84 | |
| *Actinomyces* | rs4146653 | G | A | 0.099 | 0.021 | 4.23E-06 | 0.061 | 0.039 | 0.12 | |
| *Actinomyces* | rs71315246 | A | G | -0.097 | 0.022 | 9.72E-06 | -0.074 | 0.040 | 0.07 | |
| *Actinomyces* | rs7915461 | T | C | 0.188 | 0.040 | 2.94E-06 | 0.113 | 0.064 | 0.08 | |
| *Actinomyces* | rs9565620 | C | T | -0.088 | 0.020 | 9.37E-06 | -0.005 | 0.036 | 0.89 | |
| Adlercreutzia | rs12522517 | A | T | -0.105 | 0.023 | 7.86E-06 | 0.095 | 0.054 | 0.08 | |
| Adlercreutzia | rs13231526 | C | A | 0.143 | 0.031 | 4.31E-06 | 0.110 | 0.055 | 0.05 | |
| Adlercreutzia | rs2147798 | C | G | 0.092 | 0.019 | 1.55E-06 | -0.017 | 0.039 | 0.66 | |
| Adlercreutzia | rs34181676 | G | T | -0.135 | 0.028 | 1.74E-06 | -0.008 | 0.051 | 0.87 | |
| Adlercreutzia | rs36057244 | A | G | 0.123 | 0.028 | 9.16E-06 | 0.050 | 0.053 | 0.35 | |
| Adlercreutzia | rs55719207 | G | A | -0.070 | 0.016 | 9.66E-06 | -0.010 | 0.031 | 0.75 | |
| Adlercreutzia | rs6664405 | T | C | -0.095 | 0.021 | 6.12E-06 | 0.002 | 0.045 | 0.96 | |
| Adlercreutzia | rs7680684 | C | T | -0.083 | 0.017 | 7.95E-07 | 0.009 | 0.032 | 0.78 | |
| Adlercreutzia | rs80078995 | A | T | -0.113 | 0.023 | 1.12E-06 | 0.102 | 0.045 | 0.02 | |
| Adlercreutzia | rs9490822 | C | T | -0.073 | 0.016 | 2.42E-06 | -0.006 | 0.029 | 0.84 | |
| Adlercreutzia | rs9915817 | T | C | 0.075 | 0.017 | 8.55E-06 | 0.074 | 0.032 | 0.02 | |
| Akkermansia | rs11184341 | G | C | 0.066 | 0.014 | 4.01E-06 | -0.034 | 0.032 | 0.28 | |
| Akkermansia | rs117107102 | A | G | 0.204 | 0.043 | 2.18E-06 | -0.070 | 0.077 | 0.36 | |
| Akkermansia | rs11729256 | T | C | 0.075 | 0.015 | 5.82E-07 | 0.000 | 0.033 | 1.00 | |
| Akkermansia | rs12908520 | G | A | 0.062 | 0.013 | 2.39E-06 | 0.035 | 0.030 | 0.24 | |
| Akkermansia | rs2602429 | C | T | 0.075 | 0.016 | 1.83E-06 | -0.079 | 0.036 | 0.03 | |
| Akkermansia | rs34896295 | A | T | -0.108 | 0.024 | 8.44E-06 | -0.024 | 0.054 | 0.65 | |
| Akkermansia | rs4242783 | G | A | 0.069 | 0.015 | 3.47E-06 | 0.059 | 0.035 | 0.10 | |
| Akkermansia | rs4936098 | A | G | 0.065 | 0.014 | 1.79E-06 | -0.035 | 0.031 | 0.25 | |
| Akkermansia | rs61779207 | G | A | -0.076 | 0.017 | 5.81E-06 | -0.041 | 0.038 | 0.28 | |
| Akkermansia | rs7100838 | G | A | 0.077 | 0.017 | 9.42E-06 | -0.023 | 0.039 | 0.56 | |
| Akkermansia | rs72999540 | T | C | -0.127 | 0.028 | 8.15E-06 | 0.055 | 0.068 | 0.42 | |
| Akkermansia | rs74542928 | T | C | 0.113 | 0.024 | 1.90E-06 | 0.052 | 0.048 | 0.28 | |
| Akkermansia | rs9349825 | A | G | -0.070 | 0.015 | 1.75E-06 | -0.010 | 0.033 | 0.75 | |
| Alistipes | rs1107244 | G | A | 0.076 | 0.017 | 9.37E-06 | -0.065 | 0.052 | 0.21 | |
| Alistipes | rs11769002 | G | A | -0.053 | 0.011 | 1.34E-06 | 0.032 | 0.031 | 0.29 | |
| Alistipes | rs11958296 | A | G | -0.098 | 0.022 | 6.94E-06 | 0.023 | 0.059 | 0.69 | |
| Alistipes | rs12990744 | C | T | -0.078 | 0.017 | 7.14E-06 | 0.030 | 0.048 | 0.53 | |
| Alistipes | rs1339725 | T | A | 0.089 | 0.020 | 8.55E-06 | -0.063 | 0.055 | 0.25 | |
| Alistipes | rs1689282 | A | C | -0.052 | 0.011 | 5.01E-06 | -0.076 | 0.031 | 0.02 | |
| Alistipes | rs2837096 | A | G | 0.090 | 0.020 | 5.05E-06 | -0.019 | 0.058 | 0.74 | |
| Alistipes | rs2875322 | T | C | -0.058 | 0.013 | 9.87E-06 | -0.023 | 0.042 | 0.57 | |
| Alistipes | rs34417064 | A | G | -0.048 | 0.011 | 6.47E-06 | -0.025 | 0.029 | 0.39 | |
| Alistipes | rs36021379 | A | G | -0.064 | 0.015 | 9.73E-06 | 0.009 | 0.041 | 0.84 | |
| Alistipes | rs4810359 | A | G | -0.065 | 0.015 | 8.05E-06 | -0.014 | 0.040 | 0.72 | |
| Alistipes | rs62576416 | T | C | 0.049 | 0.011 | 7.14E-06 | -0.038 | 0.030 | 0.20 | |
| Alistipes | rs67281112 | G | C | 0.063 | 0.014 | 4.15E-06 | -0.010 | 0.040 | 0.81 | |
| Alistipes | rs67705352 | T | G | -0.053 | 0.011 | 1.57E-06 | 0.000 | 0.031 | 1.00 | |
| Alistipes | rs7129639 | C | A | -0.052 | 0.011 | 1.66E-06 | 0.026 | 0.030 | 0.39 | |
| Alistipes | rs8130320 | A | G | -0.049 | 0.011 | 4.82E-06 | -0.002 | 0.029 | 0.95 | |
| Allisonella | rs1901739 | T | G | 0.116 | 0.025 | 3.22E-06 | 0.032 | 0.029 | 0.27 | |
| Allisonella | rs35110698 | T | C | -0.146 | 0.032 | 5.11E-06 | 0.036 | 0.039 | 0.36 | |
| Allisonella | rs35778461 | C | T | 0.147 | 0.030 | 7.99E-07 | -0.038 | 0.036 | 0.30 | |
| Allisonella | rs594561 | C | T | 0.112 | 0.025 | 8.22E-06 | -0.029 | 0.029 | 0.32 | |
| Allisonella | rs602075 | A | G | 0.169 | 0.030 | 1.27E-08 | 0.023 | 0.033 | 0.49 | |
| Allisonella | rs6742198 | G | A | 0.149 | 0.032 | 2.44E-06 | -0.005 | 0.037 | 0.89 | |
| Allisonella | rs76904847 | G | A | 0.149 | 0.033 | 9.19E-06 | -0.013 | 0.044 | 0.77 | |
| Allisonella | rs7803657 | A | G | -0.150 | 0.034 | 8.45E-06 | 0.066 | 0.045 | 0.14 | |
| Allisonella | rs7898615 | T | G | 0.168 | 0.037 | 6.92E-06 | -0.032 | 0.047 | 0.50 | |
| Alloprevotella | rs10902921 | G | A | -0.178 | 0.039 | 4.89E-06 | -0.020 | 0.043 | 0.65 | |
| Alloprevotella | rs117927780 | T | G | 0.121 | 0.027 | 5.43E-06 | -0.001 | 0.029 | 0.98 | |
| Alloprevotella | rs12675596 | G | T | 0.146 | 0.029 | 5.41E-07 | -0.045 | 0.034 | 0.18 | |
| Alloprevotella | rs17380632 | A | T | 0.126 | 0.028 | 7.54E-06 | 0.027 | 0.032 | 0.40 | |
| Alloprevotella | rs1956760 | T | C | 0.132 | 0.030 | 9.09E-06 | 0.003 | 0.033 | 0.93 | |
| Alloprevotella | rs2154444 | T | G | 0.138 | 0.031 | 7.67E-06 | -0.055 | 0.037 | 0.14 | |
| Alloprevotella | rs34619204 | G | A | -0.156 | 0.034 | 5.88E-06 | -0.024 | 0.037 | 0.52 | |
| Alloprevotella | rs4364940 | A | G | 0.126 | 0.028 | 7.48E-06 | 0.043 | 0.034 | 0.21 | |
| Alloprevotella | rs4680035 | A | G | -0.120 | 0.026 | 4.03E-06 | -0.034 | 0.029 | 0.25 | |
| Alloprevotella | rs500765 | G | A | -0.149 | 0.033 | 7.16E-06 | -0.027 | 0.039 | 0.49 | |
| Alloprevotella | rs58212166 | A | G | -0.162 | 0.036 | 6.88E-06 | 0.008 | 0.043 | 0.85 | |
| Anaerofilum | rs10794359 | T | C | -0.095 | 0.020 | 2.00E-06 | 0.008 | 0.032 | 0.80 | |
| Anaerofilum | rs1563175 | A | C | 0.092 | 0.020 | 4.88E-06 | -0.018 | 0.030 | 0.55 | |
| Anaerofilum | rs17012738 | T | G | 0.090 | 0.020 | 6.43E-06 | 0.013 | 0.029 | 0.65 | |
| Anaerofilum | rs17096874 | C | T | -0.126 | 0.027 | 2.63E-06 | -0.078 | 0.040 | 0.05 | |
| Anaerofilum | rs17105491 | G | C | -0.193 | 0.041 | 2.65E-06 | -0.060 | 0.060 | 0.31 | |
| Anaerofilum | rs356049 | G | A | 0.133 | 0.029 | 4.75E-06 | 0.019 | 0.046 | 0.67 | |
| Anaerofilum | rs4244069 | G | A | -0.147 | 0.033 | 6.99E-06 | 0.023 | 0.049 | 0.65 | |
| Anaerofilum | rs4506496 | G | A | 0.103 | 0.021 | 1.28E-06 | -0.002 | 0.031 | 0.96 | |
| Anaerofilum | rs712981 | A | C | 0.101 | 0.020 | 6.84E-07 | 0.015 | 0.030 | 0.60 | |
| Anaerofilum | rs79598899 | C | T | 0.183 | 0.036 | 3.22E-07 | -0.024 | 0.060 | 0.68 | |
| Anaerofilum | rs816292 | T | C | -0.113 | 0.022 | 2.94E-07 | -0.009 | 0.033 | 0.78 | |
| Anaerofilum | rs9299345 | T | C | -0.136 | 0.030 | 6.45E-06 | 0.002 | 0.044 | 0.97 | |
| Anaerostipes | rs2014785 | T | C | 0.052 | 0.011 | 4.30E-06 | -0.001 | 0.030 | 0.96 | |
| Anaerostipes | rs2396460 | T | C | -0.051 | 0.011 | 2.88E-06 | 0.031 | 0.032 | 0.34 | |
| Anaerostipes | rs2804244 | A | G | -0.053 | 0.011 | 1.72E-06 | -0.012 | 0.029 | 0.67 | |
| Anaerostipes | rs3900776 | G | A | -0.110 | 0.024 | 3.23E-06 | 0.053 | 0.063 | 0.40 | |
| Anaerostipes | rs62157625 | T | C | 0.089 | 0.019 | 1.81E-06 | 0.025 | 0.050 | 0.62 | |
| Anaerostipes | rs62215703 | G | A | 0.064 | 0.014 | 2.38E-06 | -0.033 | 0.037 | 0.36 | |
| Anaerostipes | rs6474958 | A | G | -0.050 | 0.011 | 8.01E-06 | -0.049 | 0.030 | 0.10 | |
| Anaerostipes | rs6726833 | C | A | -0.088 | 0.019 | 3.61E-06 | 0.001 | 0.055 | 0.98 | |
| Anaerostipes | rs6854026 | T | C | -0.051 | 0.011 | 3.14E-06 | -0.043 | 0.029 | 0.14 | |
| Anaerostipes | rs7193624 | C | T | 0.075 | 0.015 | 6.35E-07 | 0.043 | 0.040 | 0.28 | |
| Anaerostipes | rs7823228 | G | C | -0.062 | 0.014 | 5.46E-06 | 0.003 | 0.037 | 0.94 | |
| Anaerostipes | rs78735375 | A | C | -0.137 | 0.031 | 6.75E-06 | 0.034 | 0.071 | 0.63 | |
| Anaerotruncus | rs10150232 | A | G | 0.057 | 0.012 | 5.59E-06 | 0.023 | 0.034 | 0.51 | |
| Anaerotruncus | rs115414803 | A | C | -0.144 | 0.032 | 5.46E-06 | -0.180 | 0.071 | 0.01 | |
| Anaerotruncus | rs1272208 | G | T | -0.061 | 0.013 | 2.45E-06 | -0.010 | 0.036 | 0.79 | |
| Anaerotruncus | rs1431492 | C | T | -0.065 | 0.015 | 7.45E-06 | -0.059 | 0.039 | 0.12 | |
| Anaerotruncus | rs17734739 | T | C | 0.066 | 0.015 | 9.53E-06 | -0.027 | 0.038 | 0.47 | |
| Anaerotruncus | rs4669806 | G | T | 0.058 | 0.012 | 2.78E-06 | 0.000 | 0.033 | 1.00 | |
| Anaerotruncus | rs6494922 | A | G | 0.090 | 0.020 | 8.00E-06 | 0.025 | 0.055 | 0.65 | |
| Anaerotruncus | rs6563550 | T | C | 0.088 | 0.018 | 6.95E-07 | -0.067 | 0.052 | 0.20 | |
| Anaerotruncus | rs7155595 | C | A | 0.054 | 0.012 | 5.73E-06 | -0.041 | 0.031 | 0.18 | |
| Anaerotruncus | rs7675045 | A | T | -0.050 | 0.011 | 6.73E-06 | 0.043 | 0.029 | 0.14 | |
| Anaerotruncus | rs8005030 | C | T | 0.055 | 0.012 | 2.54E-06 | 0.036 | 0.031 | 0.25 | |
| Anaerotruncus | rs9347879 | T | C | 0.051 | 0.011 | 4.62E-06 | 0.031 | 0.029 | 0.29 | |
| Bacteroides | rs11585893 | A | G | -0.074 | 0.015 | 5.24E-07 | -0.068 | 0.045 | 0.13 | |
| Bacteroides | rs13207588 | A | G | -0.059 | 0.013 | 6.40E-06 | 0.066 | 0.045 | 0.14 | |
| Bacteroides | rs1340391 | T | C | -0.059 | 0.013 | 7.58E-06 | 0.024 | 0.037 | 0.52 | |
| Bacteroides | rs17619981 | T | G | 0.088 | 0.019 | 2.46E-06 | -0.012 | 0.052 | 0.82 | |
| Bacteroides | rs2023437 | T | C | -0.078 | 0.017 | 3.06E-06 | 0.019 | 0.052 | 0.72 | |
| Bacteroides | rs2366421 | T | A | -0.053 | 0.012 | 6.50E-06 | -0.031 | 0.032 | 0.34 | |
| Bacteroides | rs28757219 | T | A | 0.082 | 0.017 | 1.55E-06 | 0.153 | 0.044 | 0.00 | |
| Bacteroides | rs495004 | C | G | -0.061 | 0.013 | 2.90E-06 | 0.023 | 0.039 | 0.55 | |
| Bacteroides | rs66474973 | G | T | 0.081 | 0.016 | 7.81E-07 | -0.116 | 0.047 | 0.01 | |
| Bacteroides | rs66710942 | C | T | 0.049 | 0.011 | 5.53E-06 | -0.019 | 0.030 | 0.53 | |
| Bacteroides | rs6795673 | C | T | 0.054 | 0.011 | 3.11E-07 | 0.073 | 0.029 | 0.01 | |
| Bacteroides | rs9507307 | C | T | 0.060 | 0.013 | 2.85E-06 | 0.011 | 0.038 | 0.78 | |
| Barnesiella | rs11155559 | T | C | 0.096 | 0.021 | 7.15E-06 | -0.032 | 0.051 | 0.52 | |
| Barnesiella | rs113258194 | A | G | 0.099 | 0.021 | 3.78E-06 | -0.005 | 0.055 | 0.93 | |
| Barnesiella | rs12909713 | C | T | -0.055 | 0.012 | 4.48E-06 | 0.027 | 0.029 | 0.36 | |
| Barnesiella | rs13242616 | T | C | -0.058 | 0.012 | 2.15E-06 | 0.019 | 0.033 | 0.56 | |
| Barnesiella | rs199035 | G | A | 0.056 | 0.012 | 2.96E-06 | 0.021 | 0.030 | 0.49 | |
| Barnesiella | rs2057922 | G | C | 0.092 | 0.019 | 2.57E-06 | 0.001 | 0.049 | 0.98 | |
| Barnesiella | rs2276875 | A | G | -0.070 | 0.014 | 5.84E-07 | -0.052 | 0.039 | 0.19 | |
| Barnesiella | rs2428166 | G | A | -0.166 | 0.034 | 8.78E-07 | -0.010 | 0.065 | 0.88 | |
| Barnesiella | rs28418786 | C | G | -0.079 | 0.017 | 6.10E-06 | 0.035 | 0.045 | 0.43 | |
| Barnesiella | rs35177866 | A | G | 0.092 | 0.019 | 1.41E-06 | 0.000 | 0.055 | 1.00 | |
| Barnesiella | rs60316894 | C | T | -0.121 | 0.025 | 1.41E-06 | -0.079 | 0.070 | 0.26 | |
| Barnesiella | rs62251337 | A | G | -0.069 | 0.015 | 3.76E-06 | -0.059 | 0.036 | 0.10 | |
| Barnesiella | rs72684847 | T | C | -0.114 | 0.025 | 6.64E-06 | -0.063 | 0.061 | 0.31 | |
| Barnesiella | rs76181748 | C | T | -0.078 | 0.017 | 5.73E-06 | 0.040 | 0.042 | 0.34 | |
| Barnesiella | rs77455852 | T | G | -0.089 | 0.020 | 5.17E-06 | 0.028 | 0.052 | 0.59 | |
| Barnesiella | rs79795328 | A | G | -0.082 | 0.018 | 3.49E-06 | 0.030 | 0.042 | 0.48 | |
| Barnesiella | rs9372872 | C | G | -0.082 | 0.018 | 5.74E-06 | 0.040 | 0.049 | 0.41 | |
| Bifidobacterium | rs10841473 | G | C | -0.062 | 0.013 | 1.42E-06 | 0.002 | 0.033 | 0.95 | |
| Bifidobacterium | rs10923462 | T | G | -0.122 | 0.027 | 7.66E-06 | 0.116 | 0.064 | 0.07 | |
| Bifidobacterium | rs12022129 | G | A | 0.062 | 0.014 | 8.28E-06 | 0.036 | 0.037 | 0.33 | |
| Bifidobacterium | rs182549 | C | T | 0.120 | 0.013 | 5.27E-21 | -0.003 | 0.036 | 0.93 | |
| Bifidobacterium | rs2491158 | G | A | 0.071 | 0.016 | 8.25E-06 | 0.061 | 0.039 | 0.12 | |
| Bifidobacterium | rs2686790 | T | C | 0.071 | 0.016 | 7.49E-06 | -0.028 | 0.041 | 0.50 | |
| Bifidobacterium | rs4567981 | T | A | 0.056 | 0.012 | 1.87E-06 | -0.056 | 0.029 | 0.06 | |
| Bifidobacterium | rs540489 | T | G | -0.064 | 0.014 | 4.31E-06 | 0.005 | 0.037 | 0.89 | |
| Bifidobacterium | rs55888705 | A | G | 0.055 | 0.012 | 6.49E-06 | -0.024 | 0.030 | 0.43 | |
| Bifidobacterium | rs56108664 | T | C | 0.073 | 0.016 | 3.80E-06 | -0.077 | 0.036 | 0.03 | |
| Bifidobacterium | rs5746486 | T | C | -0.054 | 0.012 | 9.04E-06 | 0.038 | 0.030 | 0.20 | |
| Bifidobacterium | rs62181700 | G | A | -0.062 | 0.013 | 1.93E-06 | 0.013 | 0.033 | 0.70 | |
| Bifidobacterium | rs7322849 | T | C | 0.112 | 0.020 | 2.53E-08 | 0.060 | 0.067 | 0.37 | |
| Bifidobacterium | rs73797465 | T | G | -0.095 | 0.021 | 5.18E-06 | -0.180 | 0.064 | 0.01 | |
| Bifidobacterium | rs75344046 | C | T | 0.232 | 0.051 | 4.39E-06 | 0.079 | 0.077 | 0.30 | |
| Bifidobacterium | rs76671854 | C | G | -0.085 | 0.018 | 4.26E-06 | 0.019 | 0.052 | 0.72 | |
| Bifidobacterium | rs7971116 | G | A | 0.053 | 0.012 | 8.19E-06 | 0.007 | 0.030 | 0.81 | |
| Bifidobacterium | rs857444 | C | T | 0.056 | 0.012 | 4.12E-06 | -0.040 | 0.031 | 0.20 | |
| Bilophila | rs116261629 | G | C | 0.128 | 0.026 | 9.50E-07 | 0.052 | 0.062 | 0.39 | |
| Bilophila | rs1241171 | G | A | -0.069 | 0.015 | 3.97E-06 | 0.002 | 0.036 | 0.97 | |
| Bilophila | rs12912081 | G | A | -0.096 | 0.022 | 8.37E-06 | -0.014 | 0.053 | 0.80 | |
| Bilophila | rs1571225 | C | T | 0.083 | 0.017 | 1.26E-06 | -0.015 | 0.043 | 0.72 | |
| Bilophila | rs1917709 | A | T | 0.119 | 0.027 | 9.35E-06 | -0.036 | 0.060 | 0.55 | |
| Bilophila | rs1969927 | G | A | 0.056 | 0.013 | 8.67E-06 | -0.012 | 0.031 | 0.70 | |
| Bilophila | rs3827020 | C | T | 0.077 | 0.016 | 1.83E-06 | -0.033 | 0.041 | 0.42 | |
| Bilophila | rs542415 | T | C | -0.061 | 0.013 | 4.25E-06 | 0.005 | 0.031 | 0.87 | |
| Bilophila | rs6793291 | C | A | 0.113 | 0.024 | 3.08E-06 | 0.032 | 0.057 | 0.57 | |
| Bilophila | rs72676854 | T | C | 0.123 | 0.027 | 4.58E-06 | -0.144 | 0.062 | 0.02 | |
| Bilophila | rs7802841 | C | A | 0.067 | 0.014 | 1.14E-06 | 0.061 | 0.033 | 0.06 | |
| Bilophila | rs8013541 | A | T | -0.057 | 0.013 | 5.33E-06 | 0.029 | 0.029 | 0.32 | |
| Blautia | rs11149971 | C | T | 0.118 | 0.023 | 4.96E-07 | 0.170 | 0.065 | 0.01 | |
| Blautia | rs113271346 | C | T | 0.078 | 0.017 | 5.43E-06 | 0.042 | 0.048 | 0.37 | |
| Blautia | rs115043014 | G | A | -0.207 | 0.044 | 2.65E-06 | 0.040 | 0.069 | 0.57 | |
| Blautia | rs117001700 | T | C | 0.196 | 0.044 | 8.48E-06 | -0.011 | 0.069 | 0.87 | |
| Blautia | rs12453000 | C | T | 0.063 | 0.013 | 1.50E-06 | -0.036 | 0.040 | 0.36 | |
| Blautia | rs3005511 | A | G | 0.050 | 0.011 | 6.08E-06 | 0.013 | 0.030 | 0.68 | |
| Blautia | rs4926264 | T | C | 0.083 | 0.018 | 3.56E-06 | 0.070 | 0.056 | 0.21 | |
| Blautia | rs67794373 | C | T | 0.060 | 0.012 | 1.09E-06 | -0.043 | 0.035 | 0.23 | |
| Blautia | rs682885 | A | G | -0.049 | 0.011 | 4.32E-06 | -0.024 | 0.029 | 0.42 | |
| Blautia | rs72973581 | A | G | 0.125 | 0.027 | 2.40E-06 | -0.008 | 0.069 | 0.90 | |
| Blautia | rs73324998 | T | C | 0.181 | 0.040 | 6.74E-06 | -0.021 | 0.071 | 0.77 | |
| Butyricicoccus | rs10084203 | A | G | 0.055 | 0.012 | 8.64E-06 | -0.073 | 0.034 | 0.03 | |
| Butyricicoccus | rs12034718 | A | G | 0.070 | 0.016 | 9.34E-06 | 0.031 | 0.043 | 0.47 | |
| Butyricicoccus | rs12585793 | T | C | -0.262 | 0.056 | 3.43E-06 | 0.143 | 0.074 | 0.05 | |
| Butyricicoccus | rs2017189 | G | T | -0.051 | 0.011 | 4.25E-06 | -0.020 | 0.029 | 0.50 | |
| Butyricicoccus | rs2146325 | G | A | 0.080 | 0.018 | 9.28E-06 | -0.035 | 0.048 | 0.47 | |
| Butyricicoccus | rs3737495 | A | G | -0.071 | 0.016 | 7.38E-06 | -0.009 | 0.045 | 0.85 | |
| Butyricicoccus | rs4962426 | G | T | 0.061 | 0.014 | 6.27E-06 | -0.070 | 0.041 | 0.09 | |
| Butyricicoccus | rs56221232 | T | C | 0.083 | 0.017 | 7.56E-07 | -0.075 | 0.046 | 0.10 | |
| Butyricicoccus | rs62478070 | T | G | 0.224 | 0.049 | 6.00E-06 | 0.027 | 0.068 | 0.69 | |
| Butyricicoccus | rs7322368 | T | C | 0.082 | 0.018 | 8.45E-06 | 0.089 | 0.050 | 0.07 | |
| Butyricicoccus | rs75238760 | T | A | 0.062 | 0.014 | 9.59E-06 | -0.051 | 0.038 | 0.18 | |
| Butyricimonas | rs11228830 | A | G | 0.135 | 0.030 | 5.82E-06 | -0.065 | 0.056 | 0.25 | |
| Butyricimonas | rs113054641 | G | A | -0.145 | 0.027 | 1.32E-07 | 0.016 | 0.060 | 0.79 | |
| Butyricimonas | rs12458763 | A | C | 0.122 | 0.027 | 5.99E-06 | 0.000 | 0.057 | 1.00 | |
| Butyricimonas | rs1862649 | G | A | 0.113 | 0.025 | 5.16E-06 | -0.057 | 0.052 | 0.27 | |
| Butyricimonas | rs2114713 | G | T | 0.063 | 0.014 | 6.41E-06 | -0.060 | 0.030 | 0.04 | |
| Butyricimonas | rs2642760 | G | C | 0.071 | 0.015 | 9.28E-07 | 0.020 | 0.030 | 0.52 | |
| Butyricimonas | rs270727 | G | C | -0.069 | 0.015 | 4.16E-06 | 0.050 | 0.035 | 0.15 | |
| Butyricimonas | rs326049 | C | G | 0.076 | 0.017 | 5.83E-06 | 0.007 | 0.042 | 0.87 | |
| Butyricimonas | rs4145834 | A | G | 0.086 | 0.019 | 9.15E-06 | 0.000 | 0.044 | 1.00 | |
| Butyricimonas | rs62130338 | G | A | -0.073 | 0.016 | 3.71E-06 | -0.030 | 0.036 | 0.40 | |
| Butyricimonas | rs62390301 | T | C | -0.087 | 0.017 | 5.99E-07 | -0.027 | 0.039 | 0.49 | |
| Butyricimonas | rs7083431 | A | C | 0.070 | 0.014 | 1.11E-06 | 0.058 | 0.031 | 0.06 | |
| Butyricimonas | rs71428626 | G | T | -0.133 | 0.029 | 4.34E-06 | -0.034 | 0.058 | 0.55 | |
| Butyricimonas | rs72814525 | A | G | 0.066 | 0.015 | 9.22E-06 | -0.008 | 0.031 | 0.79 | |
| Butyricimonas | rs782080 | T | A | 0.065 | 0.014 | 2.18E-06 | 0.010 | 0.029 | 0.75 | |
| Butyricimonas | rs78453362 | A | G | -0.149 | 0.033 | 4.87E-06 | -0.019 | 0.068 | 0.78 | |
| Butyricimonas | rs9657374 | C | T | 0.068 | 0.015 | 4.35E-06 | -0.016 | 0.032 | 0.61 | |
| Butyrivibrio | rs1007475 | G | T | 0.118 | 0.026 | 6.22E-06 | 0.012 | 0.032 | 0.71 | |
| Butyrivibrio | rs11761679 | T | C | 0.155 | 0.032 | 1.48E-06 | 0.001 | 0.038 | 0.98 | |
| Butyrivibrio | rs13409081 | C | G | 0.138 | 0.031 | 9.76E-06 | -0.005 | 0.038 | 0.90 | |
| Butyrivibrio | rs16934069 | T | C | -0.134 | 0.030 | 7.90E-06 | -0.009 | 0.039 | 0.81 | |
| Butyrivibrio | rs16941336 | C | T | 0.127 | 0.027 | 1.95E-06 | -0.100 | 0.033 | 0.00 | |
| Butyrivibrio | rs17163238 | G | A | 0.141 | 0.031 | 5.09E-06 | 0.086 | 0.036 | 0.02 | |
| Butyrivibrio | rs28846706 | A | G | 0.176 | 0.039 | 7.13E-06 | 0.033 | 0.050 | 0.51 | |
| Butyrivibrio | rs4537857 | T | C | -0.125 | 0.026 | 1.81E-06 | 0.007 | 0.032 | 0.82 | |
| Butyrivibrio | rs486484 | A | G | -0.108 | 0.024 | 6.35E-06 | -0.037 | 0.029 | 0.20 | |
| Butyrivibrio | rs4928024 | A | G | -0.175 | 0.039 | 7.21E-06 | -0.031 | 0.051 | 0.54 | |
| Butyrivibrio | rs72723662 | C | T | 0.224 | 0.045 | 6.14E-07 | 0.068 | 0.060 | 0.26 | |
| Butyrivibrio | rs7412979 | C | G | 0.187 | 0.039 | 1.59E-06 | -0.010 | 0.046 | 0.83 | |
| Butyrivibrio | rs74622183 | A | G | -0.201 | 0.043 | 2.67E-06 | -0.064 | 0.055 | 0.25 | |
| Butyrivibrio | rs7752361 | A | G | -0.119 | 0.024 | 6.75E-07 | 0.009 | 0.030 | 0.75 | |
| Butyrivibrio | rs7763512 | G | A | 0.120 | 0.025 | 2.24E-06 | 0.007 | 0.030 | 0.81 | |
| Butyrivibrio | rs9349693 | A | G | 0.118 | 0.026 | 5.63E-06 | 0.021 | 0.032 | 0.50 | |
| CandidatusSoleaferrea | rs10090365 | A | G | -0.083 | 0.018 | 4.02E-06 | 0.043 | 0.029 | 0.14 | |
| CandidatusSoleaferrea | rs10108780 | A | G | -0.093 | 0.020 | 3.38E-06 | -0.011 | 0.033 | 0.73 | |
| CandidatusSoleaferrea | rs10809135 | T | C | 0.083 | 0.018 | 4.73E-06 | -0.032 | 0.030 | 0.30 | |
| CandidatusSoleaferrea | rs11153159 | G | C | -0.128 | 0.029 | 7.26E-06 | 0.085 | 0.046 | 0.07 | |
| CandidatusSoleaferrea | rs12500231 | A | T | 0.081 | 0.018 | 8.40E-06 | -0.024 | 0.030 | 0.43 | |
| CandidatusSoleaferrea | rs2193878 | T | A | 0.228 | 0.051 | 7.39E-06 | -0.069 | 0.068 | 0.31 | |
| CandidatusSoleaferrea | rs386526 | C | G | 0.082 | 0.018 | 5.50E-06 | 0.038 | 0.031 | 0.22 | |
| CandidatusSoleaferrea | rs4678258 | T | C | 0.099 | 0.022 | 4.81E-06 | 0.019 | 0.037 | 0.61 | |
| CandidatusSoleaferrea | rs61825792 | T | C | 0.112 | 0.023 | 1.30E-06 | -0.054 | 0.037 | 0.14 | |
| CandidatusSoleaferrea | rs6489992 | A | G | -0.084 | 0.019 | 7.00E-06 | 0.003 | 0.030 | 0.93 | |
| CandidatusSoleaferrea | rs6494306 | A | G | -0.097 | 0.021 | 6.05E-06 | -0.021 | 0.034 | 0.55 | |
| CandidatusSoleaferrea | rs6881988 | G | C | -0.082 | 0.018 | 6.66E-06 | 0.034 | 0.035 | 0.32 | |
| CandidatusSoleaferrea | rs7400877 | T | C | -0.095 | 0.021 | 7.84E-06 | 0.032 | 0.035 | 0.36 | |
| CandidatusSoleaferrea | rs830149 | C | G | 0.185 | 0.040 | 3.35E-06 | 0.119 | 0.070 | 0.09 | |
| CandidatusSoleaferrea | rs9973954 | A | G | 0.089 | 0.020 | 4.99E-06 | 0.030 | 0.032 | 0.34 | |
| Catenibacterium | rs10911474 | G | A | -0.117 | 0.026 | 8.77E-06 | 0.021 | 0.032 | 0.52 | |
| Catenibacterium | rs12404911 | C | T | 0.141 | 0.030 | 3.72E-06 | -0.071 | 0.037 | 0.05 | |
| Catenibacterium | rs212393 | G | A | -0.135 | 0.029 | 2.29E-06 | -0.011 | 0.031 | 0.71 | |
| Catenibacterium | rs73128290 | A | G | 0.130 | 0.028 | 5.14E-06 | -0.014 | 0.033 | 0.66 | |
| Catenibacterium | rs77285108 | G | A | -0.162 | 0.035 | 4.68E-06 | 0.085 | 0.042 | 0.04 | |
| Catenibacterium | rs7742829 | C | T | 0.114 | 0.025 | 5.51E-06 | -0.041 | 0.030 | 0.17 | |
| ChristensenellaceaeR | rs10461257 | A | G | -0.055 | 0.012 | 6.13E-06 | -0.073 | 0.036 | 0.04 | |
| ChristensenellaceaeR | rs16930076 | G | T | -0.066 | 0.015 | 9.70E-06 | -0.015 | 0.040 | 0.72 | |
| ChristensenellaceaeR | rs17081797 | A | G | -0.090 | 0.020 | 9.53E-06 | -0.020 | 0.056 | 0.73 | |
| ChristensenellaceaeR | rs4076564 | G | A | -0.178 | 0.040 | 8.67E-06 | 0.050 | 0.079 | 0.53 | |
| ChristensenellaceaeR | rs60954665 | T | G | 0.050 | 0.011 | 6.91E-06 | -0.046 | 0.029 | 0.12 | |
| ChristensenellaceaeR | rs62132810 | A | G | -0.083 | 0.018 | 3.94E-06 | 0.092 | 0.058 | 0.11 | |
| ChristensenellaceaeR | rs62190261 | A | C | 0.096 | 0.021 | 8.07E-06 | -0.016 | 0.059 | 0.79 | |
| ChristensenellaceaeR | rs62467127 | C | T | 0.114 | 0.025 | 5.94E-06 | 0.010 | 0.064 | 0.88 | |
| ChristensenellaceaeR | rs73952017 | C | T | -0.086 | 0.019 | 9.16E-06 | 0.028 | 0.052 | 0.59 | |
| ChristensenellaceaeR | rs78521377 | C | T | 0.125 | 0.027 | 5.40E-06 | -0.014 | 0.066 | 0.83 | |
| ChristensenellaceaeR | rs79150079 | C | A | 0.122 | 0.027 | 7.27E-06 | -0.010 | 0.064 | 0.87 | |
| ChristensenellaceaeR | rs892686 | A | G | 0.051 | 0.011 | 3.90E-06 | -0.027 | 0.029 | 0.35 | |
| ChristensenellaceaeR | rs999354 | T | A | 0.058 | 0.012 | 7.36E-07 | -0.008 | 0.031 | 0.78 | |
| *Clostridium sensustricto 1* | rs115807074 | A | G | -0.227 | 0.049 | 3.92E-06 | -0.007 | 0.071 | 0.93 | |
| *Clostridium sensustricto 1* | rs11586026 | A | T | 0.111 | 0.025 | 9.43E-06 | -0.098 | 0.058 | 0.09 | |
| *Clostridium sensustricto 1* | rs116847295 | C | T | 0.110 | 0.025 | 7.75E-06 | -0.029 | 0.062 | 0.64 | |
| *Clostridium sensustricto 1* | rs12186080 | G | A | 0.073 | 0.016 | 7.96E-06 | 0.022 | 0.039 | 0.57 | |
| *Clostridium sensustricto 1* | rs12341505 | G | A | 0.081 | 0.018 | 6.76E-06 | -0.022 | 0.045 | 0.62 | |
| *Clostridium sensustricto 1* | rs12490337 | C | G | -0.062 | 0.014 | 7.33E-06 | -0.044 | 0.034 | 0.20 | |
| *Clostridium sensustricto 1* | rs17394442 | T | C | -0.156 | 0.035 | 7.94E-06 | -0.029 | 0.077 | 0.71 | |
| *Clostridium sensustricto 1* | rs2795528 | G | A | -0.184 | 0.039 | 2.61E-06 | 0.075 | 0.064 | 0.24 | |
| *Clostridium sensustricto 1* | rs2817172 | C | T | 0.058 | 0.012 | 3.01E-06 | 0.042 | 0.031 | 0.17 | |
| *Clostridium sensustricto 1* | rs550843 | T | C | -0.078 | 0.017 | 3.68E-06 | 0.009 | 0.044 | 0.84 | |
| Collinsella | rs10890671 | T | C | -0.054 | 0.012 | 6.13E-06 | -0.027 | 0.029 | 0.36 | |
| Collinsella | rs11597285 | G | T | -0.054 | 0.012 | 8.10E-06 | -0.001 | 0.030 | 0.97 | |
| Collinsella | rs12921100 | A | T | 0.056 | 0.013 | 9.06E-06 | -0.005 | 0.032 | 0.88 | |
| Collinsella | rs149807560 | C | A | -0.104 | 0.024 | 9.83E-06 | -0.005 | 0.056 | 0.93 | |
| Collinsella | rs2103510 | G | A | 0.079 | 0.017 | 2.93E-06 | 0.007 | 0.043 | 0.88 | |
| Collinsella | rs2671662 | C | G | -0.057 | 0.012 | 2.00E-06 | -0.034 | 0.033 | 0.30 | |
| Collinsella | rs59414781 | C | G | 0.067 | 0.015 | 7.97E-06 | -0.039 | 0.036 | 0.28 | |
| Collinsella | rs62102630 | A | G | -0.070 | 0.015 | 5.59E-06 | 0.001 | 0.039 | 0.98 | |
| Collinsella | rs62448869 | T | A | -0.054 | 0.012 | 7.01E-06 | 0.010 | 0.030 | 0.72 | |
| Collinsella | rs6758729 | A | G | -0.063 | 0.014 | 9.10E-06 | 0.007 | 0.035 | 0.84 | |
| Collinsella | rs7179874 | A | G | 0.071 | 0.016 | 6.70E-06 | 0.027 | 0.037 | 0.46 | |
| Collinsella | rs73052258 | G | A | 0.093 | 0.020 | 4.43E-06 | -0.037 | 0.050 | 0.46 | |
| Collinsella | rs75672793 | A | G | -0.109 | 0.024 | 5.95E-06 | 0.014 | 0.055 | 0.80 | |
| Collinsella | rs9541268 | C | A | 0.096 | 0.020 | 1.15E-06 | 0.060 | 0.046 | 0.19 | |
| Coprobacter | rs11532348 | C | T | -0.104 | 0.023 | 4.61E-06 | 0.054 | 0.036 | 0.14 | |
| Coprobacter | rs12684609 | T | C | 0.101 | 0.022 | 4.75E-06 | 0.006 | 0.038 | 0.87 | |
| Coprobacter | rs12908296 | A | G | 0.101 | 0.022 | 6.58E-06 | 0.011 | 0.037 | 0.76 | |
| Coprobacter | rs143180826 | C | T | 0.170 | 0.038 | 8.73E-06 | -0.037 | 0.066 | 0.57 | |
| Coprobacter | rs143662916 | C | T | 0.253 | 0.054 | 2.75E-06 | 0.069 | 0.062 | 0.26 | |
| Coprobacter | rs189356 | G | A | 0.078 | 0.017 | 5.53E-06 | -0.006 | 0.031 | 0.84 | |
| Coprobacter | rs213863 | C | T | -0.089 | 0.019 | 2.47E-06 | 0.013 | 0.032 | 0.68 | |
| Coprobacter | rs305411 | A | G | 0.129 | 0.026 | 1.03E-06 | -0.016 | 0.050 | 0.75 | |
| Coprobacter | rs3828477 | G | T | -0.091 | 0.020 | 3.14E-06 | 0.011 | 0.034 | 0.74 | |
| Coprobacter | rs55672356 | T | A | -0.193 | 0.041 | 2.98E-06 | -0.035 | 0.064 | 0.58 | |
| Coprobacter | rs57543781 | T | G | 0.079 | 0.018 | 9.33E-06 | 0.006 | 0.035 | 0.85 | |
| Coprobacter | rs6014909 | A | G | 0.077 | 0.017 | 9.28E-06 | -0.029 | 0.029 | 0.32 | |
| Coprobacter | rs62177393 | G | A | 0.120 | 0.027 | 8.54E-06 | -0.089 | 0.047 | 0.06 | |
| Coprobacter | rs72821405 | T | C | -0.147 | 0.032 | 4.12E-06 | -0.058 | 0.054 | 0.28 | |
| Coprobacter | rs74919520 | G | A | 0.126 | 0.028 | 5.38E-06 | 0.041 | 0.045 | 0.37 | |
| Coprococcus1 | rs1010560 | C | A | 0.058 | 0.012 | 2.27E-06 | -0.032 | 0.033 | 0.33 | |
| Coprococcus1 | rs12794898 | G | T | 0.090 | 0.020 | 4.64E-06 | 0.060 | 0.060 | 0.32 | |
| Coprococcus1 | rs12886051 | G | C | -0.052 | 0.012 | 9.87E-06 | -0.004 | 0.031 | 0.89 | |
| Coprococcus1 | rs1576241 | A | G | -0.051 | 0.011 | 3.17E-06 | 0.029 | 0.029 | 0.33 | |
| Coprococcus1 | rs1762123 | C | T | -0.089 | 0.020 | 7.12E-06 | 0.059 | 0.064 | 0.36 | |
| Coprococcus1 | rs2907920 | A | G | 0.056 | 0.013 | 9.65E-06 | 0.015 | 0.034 | 0.65 | |
| Coprococcus1 | rs4277593 | G | A | -0.059 | 0.011 | 9.92E-08 | 0.012 | 0.029 | 0.67 | |
| Coprococcus1 | rs56405618 | A | G | -0.090 | 0.019 | 1.54E-06 | -0.017 | 0.051 | 0.73 | |
| Coprococcus1 | rs73031725 | T | C | 0.168 | 0.036 | 2.38E-06 | 0.091 | 0.076 | 0.23 | |
| Coprococcus1 | rs73167075 | T | C | 0.057 | 0.013 | 7.04E-06 | -0.016 | 0.034 | 0.63 | |
| Coprococcus1 | rs74101919 | T | C | -0.072 | 0.014 | 6.68E-07 | 0.100 | 0.038 | 0.01 | |
| Coprococcus1 | rs7784490 | C | G | -0.052 | 0.011 | 4.58E-06 | 0.002 | 0.030 | 0.96 | |
| Coprococcus1 | rs946513 | C | T | 0.206 | 0.046 | 7.61E-06 | 0.060 | 0.081 | 0.45 | |
| Coprococcus2 | rs12634070 | T | C | 0.074 | 0.016 | 7.98E-06 | 0.046 | 0.040 | 0.25 | |
| Coprococcus2 | rs13113703 | A | G | -0.060 | 0.014 | 9.99E-06 | 0.002 | 0.031 | 0.94 | |
| Coprococcus2 | rs1958519 | T | A | 0.067 | 0.014 | 1.57E-06 | 0.010 | 0.030 | 0.75 | |
| Coprococcus2 | rs2482516 | C | T | 0.075 | 0.016 | 4.59E-06 | -0.067 | 0.036 | 0.06 | |
| Coprococcus2 | rs35890118 | A | G | -0.067 | 0.015 | 6.61E-06 | -0.009 | 0.033 | 0.79 | |
| Coprococcus2 | rs59936925 | A | T | 0.117 | 0.023 | 5.62E-07 | 0.024 | 0.053 | 0.65 | |
| Coprococcus2 | rs61823518 | A | C | -0.096 | 0.022 | 9.48E-06 | 0.024 | 0.053 | 0.65 | |
| Coprococcus2 | rs62470912 | T | G | -0.103 | 0.023 | 9.12E-06 | 0.040 | 0.051 | 0.44 | |
| Coprococcus2 | rs6677933 | C | T | -0.080 | 0.016 | 9.66E-07 | -0.023 | 0.038 | 0.54 | |
| Coprococcus2 | rs6894272 | T | C | -0.113 | 0.025 | 7.31E-06 | 0.049 | 0.056 | 0.38 | |
| Coprococcus2 | rs72680320 | T | C | -0.065 | 0.014 | 3.08E-06 | -0.006 | 0.031 | 0.86 | |
| Coprococcus2 | rs9426473 | A | G | 0.073 | 0.016 | 6.82E-06 | -0.016 | 0.035 | 0.65 | |
| Coprococcus3 | rs10810043 | A | G | 0.052 | 0.012 | 8.70E-06 | -0.043 | 0.031 | 0.18 | |
| Coprococcus3 | rs11080344 | C | T | 0.052 | 0.011 | 4.82E-06 | 0.058 | 0.029 | 0.05 | |
| Coprococcus3 | rs13394391 | C | T | -0.071 | 0.015 | 2.60E-06 | -0.027 | 0.039 | 0.48 | |
| Coprococcus3 | rs178271 | T | C | 0.145 | 0.029 | 7.95E-07 | -0.032 | 0.071 | 0.65 | |
| Coprococcus3 | rs4575475 | G | A | 0.062 | 0.014 | 6.92E-06 | -0.031 | 0.035 | 0.38 | |
| Coprococcus3 | rs62481985 | G | C | -0.058 | 0.012 | 4.15E-07 | -0.049 | 0.030 | 0.10 | |
| Coprococcus3 | rs8100692 | T | C | 0.058 | 0.011 | 3.61E-07 | -0.046 | 0.029 | 0.12 | |
| DefluviitaleaceaeUCG011 | rs112893842 | T | C | 0.114 | 0.023 | 1.02E-06 | -0.014 | 0.044 | 0.75 | |
| DefluviitaleaceaeUCG011 | rs12500663 | T | G | -0.115 | 0.023 | 4.72E-07 | -0.044 | 0.041 | 0.28 | |
| DefluviitaleaceaeUCG011 | rs1582238 | T | C | 0.081 | 0.017 | 1.49E-06 | 0.042 | 0.031 | 0.17 | |
| DefluviitaleaceaeUCG011 | rs28696126 | A | T | -0.107 | 0.024 | 7.97E-06 | -0.018 | 0.044 | 0.69 | |
| DefluviitaleaceaeUCG011 | rs2892880 | G | A | 0.082 | 0.018 | 6.77E-06 | -0.046 | 0.033 | 0.17 | |
| DefluviitaleaceaeUCG011 | rs4344384 | G | T | 0.072 | 0.016 | 4.64E-06 | 0.060 | 0.029 | 0.04 | |
| DefluviitaleaceaeUCG011 | rs4677103 | A | G | 0.098 | 0.020 | 7.14E-07 | -0.028 | 0.041 | 0.49 | |
| DefluviitaleaceaeUCG011 | rs55658617 | T | C | 0.174 | 0.036 | 1.48E-06 | -0.151 | 0.070 | 0.03 | |
| DefluviitaleaceaeUCG011 | rs75250694 | G | A | 0.116 | 0.026 | 7.56E-06 | -0.041 | 0.047 | 0.38 | |
| DefluviitaleaceaeUCG011 | rs9608282 | T | G | 0.143 | 0.030 | 1.86E-06 | -0.067 | 0.060 | 0.26 | |
| DefluviitaleaceaeUCG011 | rs9725395 | A | G | -0.138 | 0.030 | 2.86E-06 | -0.027 | 0.055 | 0.62 | |
| Desulfovibrio | rs10937554 | C | T | 0.123 | 0.027 | 6.42E-06 | -0.163 | 0.066 | 0.01 | |
| Desulfovibrio | rs11160353 | T | A | -0.069 | 0.015 | 2.64E-06 | 0.001 | 0.029 | 0.97 | |
| Desulfovibrio | rs12031543 | T | C | -0.127 | 0.028 | 6.42E-06 | 0.008 | 0.064 | 0.90 | |
| Desulfovibrio | rs13066142 | G | A | 0.119 | 0.025 | 2.05E-06 | 0.025 | 0.053 | 0.64 | |
| Desulfovibrio | rs16863365 | A | G | 0.109 | 0.023 | 1.43E-06 | 0.050 | 0.043 | 0.24 | |
| Desulfovibrio | rs17062711 | A | G | 0.071 | 0.016 | 9.47E-06 | -0.082 | 0.032 | 0.01 | |
| Desulfovibrio | rs2590913 | G | A | 0.154 | 0.034 | 5.31E-06 | 0.067 | 0.070 | 0.34 | |
| Desulfovibrio | rs2853179 | C | T | 0.081 | 0.017 | 3.16E-06 | -0.003 | 0.035 | 0.93 | |
| Desulfovibrio | rs3789086 | A | G | 0.102 | 0.023 | 9.26E-06 | -0.015 | 0.045 | 0.74 | |
| Desulfovibrio | rs4797774 | G | A | 0.213 | 0.047 | 6.11E-06 | -0.123 | 0.082 | 0.13 | |
| Desulfovibrio | rs6580353 | T | C | 0.077 | 0.017 | 5.57E-06 | -0.058 | 0.038 | 0.13 | |
| Desulfovibrio | rs72647089 | T | G | -0.107 | 0.024 | 8.44E-06 | 0.006 | 0.049 | 0.91 | |
| Desulfovibrio | rs7729080 | C | A | -0.070 | 0.016 | 8.30E-06 | -0.023 | 0.031 | 0.45 | |
| Dialister | rs10938938 | G | A | -0.077 | 0.017 | 6.02E-06 | 0.013 | 0.037 | 0.72 | |
| Dialister | rs11071887 | T | C | 0.066 | 0.015 | 5.98E-06 | -0.066 | 0.038 | 0.09 | |
| Dialister | rs11166701 | G | A | -0.066 | 0.013 | 6.71E-07 | -0.034 | 0.029 | 0.24 | |
| Dialister | rs17541540 | C | G | 0.059 | 0.013 | 9.63E-06 | 0.047 | 0.035 | 0.18 | |
| Dialister | rs2314294 | T | C | 0.087 | 0.019 | 7.82E-06 | 0.006 | 0.045 | 0.90 | |
| Dialister | rs2435610 | A | C | 0.065 | 0.014 | 6.32E-06 | -0.017 | 0.034 | 0.62 | |
| Dialister | rs4747450 | C | A | 0.067 | 0.015 | 5.99E-06 | -0.027 | 0.033 | 0.41 | |
| Dialister | rs4753063 | G | A | -0.060 | 0.013 | 4.54E-06 | -0.045 | 0.030 | 0.13 | |
| Dialister | rs517089 | T | A | 0.076 | 0.017 | 7.71E-06 | 0.049 | 0.038 | 0.20 | |
| Dialister | rs75416973 | A | G | 0.073 | 0.016 | 9.85E-06 | 0.048 | 0.038 | 0.20 | |
| Dialister | rs764177 | C | A | -0.060 | 0.014 | 8.86E-06 | 0.027 | 0.034 | 0.42 | |
| Dialister | rs76680460 | G | A | -0.161 | 0.036 | 9.55E-06 | 0.090 | 0.070 | 0.20 | |
| Dialister | rs9554744 | T | C | 0.073 | 0.016 | 6.00E-06 | 0.027 | 0.036 | 0.46 | |
| Dorea | rs11150408 | T | G | 0.049 | 0.011 | 7.58E-06 | 0.026 | 0.033 | 0.43 | |
| Dorea | rs12216169 | T | A | 0.088 | 0.019 | 5.30E-06 | -0.079 | 0.055 | 0.15 | |
| Dorea | rs12537781 | T | C | -0.056 | 0.013 | 9.18E-06 | 0.045 | 0.033 | 0.17 | |
| Dorea | rs12634859 | T | C | 0.059 | 0.013 | 9.59E-06 | 0.026 | 0.037 | 0.47 | |
| Dorea | rs13279148 | G | A | 0.072 | 0.015 | 2.13E-06 | -0.020 | 0.040 | 0.62 | |
| Dorea | rs1899291 | C | T | 0.070 | 0.015 | 3.50E-06 | -0.005 | 0.044 | 0.92 | |
| Dorea | rs3005511 | A | G | 0.052 | 0.011 | 4.80E-06 | 0.013 | 0.030 | 0.68 | |
| Dorea | rs3752849 | G | A | 0.164 | 0.037 | 7.62E-06 | -0.136 | 0.077 | 0.08 | |
| Dorea | rs4793307 | C | T | 0.057 | 0.012 | 2.76E-06 | 0.046 | 0.035 | 0.18 | |
| Dorea | rs62503162 | A | G | -0.097 | 0.019 | 5.41E-07 | 0.076 | 0.051 | 0.14 | |
| Dorea | rs62583469 | T | A | -0.063 | 0.014 | 8.25E-06 | 0.045 | 0.037 | 0.22 | |
| Dorea | rs66490379 | T | C | 0.055 | 0.012 | 9.13E-06 | 0.029 | 0.033 | 0.38 | |
| Dorea | rs73729431 | C | T | -0.137 | 0.030 | 4.61E-06 | 0.122 | 0.065 | 0.06 | |
| Eggerthella | rs112205261 | T | C | -0.189 | 0.040 | 2.96E-06 | 0.036 | 0.059 | 0.54 | |
| Eggerthella | rs13070736 | A | C | -0.121 | 0.027 | 8.18E-06 | -0.087 | 0.043 | 0.04 | |
| Eggerthella | rs1377678 | C | T | 0.090 | 0.020 | 9.02E-06 | -0.022 | 0.036 | 0.54 | |
| Eggerthella | rs1784405 | T | G | 0.091 | 0.020 | 4.58E-06 | 0.012 | 0.029 | 0.68 | |
| Eggerthella | rs2223081 | G | A | 0.103 | 0.022 | 3.50E-06 | -0.036 | 0.033 | 0.28 | |
| Eggerthella | rs2240838 | A | G | 0.098 | 0.020 | 7.01E-07 | -0.030 | 0.031 | 0.32 | |
| Eggerthella | rs2877457 | G | A | -0.093 | 0.021 | 8.69E-06 | 0.009 | 0.032 | 0.77 | |
| Eggerthella | rs3851328 | T | G | -0.108 | 0.024 | 5.20E-06 | 0.015 | 0.034 | 0.67 | |
| Eggerthella | rs4985746 | G | A | 0.111 | 0.025 | 8.09E-06 | 0.058 | 0.036 | 0.11 | |
| Eggerthella | rs6430926 | C | T | 0.088 | 0.020 | 8.09E-06 | -0.019 | 0.029 | 0.53 | |
| Eggerthella | rs67490567 | T | C | 0.108 | 0.025 | 9.87E-06 | 0.046 | 0.041 | 0.26 | |
| Eggerthella | rs76663501 | C | T | 0.175 | 0.038 | 3.62E-06 | 0.009 | 0.054 | 0.87 | |
| Eisenbergiella | rs11027642 | C | T | 0.129 | 0.028 | 5.93E-06 | 0.013 | 0.044 | 0.76 | |
| Eisenbergiella | rs11651545 | T | C | 0.107 | 0.024 | 6.85E-06 | 0.068 | 0.035 | 0.06 | |
| Eisenbergiella | rs11938607 | T | C | 0.098 | 0.022 | 6.33E-06 | -0.004 | 0.035 | 0.92 | |
| Eisenbergiella | rs12257723 | A | C | -0.095 | 0.021 | 6.69E-06 | 0.011 | 0.032 | 0.74 | |
| Eisenbergiella | rs12278566 | T | A | -0.121 | 0.025 | 1.50E-06 | 0.057 | 0.040 | 0.15 | |
| Eisenbergiella | rs12710729 | C | A | 0.089 | 0.020 | 7.20E-06 | 0.035 | 0.031 | 0.26 | |
| Eisenbergiella | rs13258851 | A | G | 0.137 | 0.030 | 5.77E-06 | -0.090 | 0.050 | 0.07 | |
| Eisenbergiella | rs1508033 | A | C | 0.092 | 0.020 | 2.93E-06 | -0.046 | 0.030 | 0.12 | |
| Eisenbergiella | rs1553971 | T | G | 0.121 | 0.026 | 4.26E-06 | -0.005 | 0.041 | 0.91 | |
| Eisenbergiella | rs2683098 | C | T | 0.107 | 0.023 | 1.87E-06 | 0.037 | 0.035 | 0.30 | |
| Eisenbergiella | rs3812426 | G | A | 0.106 | 0.022 | 2.05E-06 | 0.001 | 0.035 | 0.98 | |
| Eisenbergiella | rs4462860 | G | A | 0.094 | 0.020 | 3.02E-06 | -0.012 | 0.035 | 0.72 | |
| Enterorhabdus | rs10098492 | T | C | 0.132 | 0.029 | 6.62E-06 | -0.005 | 0.051 | 0.93 | |
| Enterorhabdus | rs10242782 | C | A | -0.076 | 0.017 | 5.10E-06 | -0.050 | 0.032 | 0.12 | |
| Enterorhabdus | rs11098863 | T | A | -0.097 | 0.016 | 2.88E-09 | -0.007 | 0.029 | 0.81 | |
| Enterorhabdus | rs114731706 | T | G | 0.182 | 0.038 | 1.85E-06 | -0.072 | 0.067 | 0.28 | |
| Enterorhabdus | rs2051957 | C | T | 0.084 | 0.019 | 8.93E-06 | -0.064 | 0.033 | 0.05 | |
| Enterorhabdus | rs3017103 | A | G | 0.098 | 0.021 | 2.69E-06 | -0.005 | 0.038 | 0.89 | |
| Enterorhabdus | rs424715 | T | C | 0.082 | 0.017 | 2.84E-06 | 0.032 | 0.036 | 0.39 | |
| Enterorhabdus | rs73331712 | T | C | 0.262 | 0.055 | 2.01E-06 | -0.169 | 0.073 | 0.02 | |
| Enterorhabdus | rs77655283 | G | A | 0.133 | 0.030 | 8.27E-06 | -0.055 | 0.056 | 0.33 | |
| Enterorhabdus | rs7923280 | A | T | 0.086 | 0.017 | 3.69E-07 | 0.013 | 0.031 | 0.66 | |
| Enterorhabdus | rs9470637 | A | T | -0.076 | 0.017 | 5.52E-06 | 0.016 | 0.030 | 0.60 | |
| Erysipelatoclostridium | rs1106058 | A | G | 0.077 | 0.017 | 8.82E-06 | 0.110 | 0.035 | 0.00 | |
| Erysipelatoclostridium | rs1434153 | G | A | -0.068 | 0.015 | 7.11E-06 | -0.030 | 0.032 | 0.34 | |
| Erysipelatoclostridium | rs16936671 | C | T | -0.097 | 0.022 | 8.88E-06 | 0.077 | 0.045 | 0.09 | |
| Erysipelatoclostridium | rs17804233 | T | C | -0.066 | 0.014 | 4.28E-06 | 0.021 | 0.029 | 0.48 | |
| Erysipelatoclostridium | rs2711883 | T | C | -0.078 | 0.018 | 9.49E-06 | 0.012 | 0.037 | 0.74 | |
| Erysipelatoclostridium | rs2901723 | C | A | 0.064 | 0.014 | 8.80E-06 | 0.014 | 0.030 | 0.63 | |
| Erysipelatoclostridium | rs340991 | A | G | -0.074 | 0.016 | 3.16E-06 | 0.028 | 0.032 | 0.37 | |
| Erysipelatoclostridium | rs34528142 | C | G | -0.088 | 0.020 | 7.56E-06 | -0.057 | 0.041 | 0.17 | |
| Erysipelatoclostridium | rs45480394 | T | G | -0.069 | 0.015 | 6.02E-06 | -0.013 | 0.031 | 0.67 | |
| Erysipelatoclostridium | rs4697572 | A | G | -0.081 | 0.016 | 6.91E-07 | 0.031 | 0.034 | 0.36 | |
| Erysipelatoclostridium | rs58236560 | G | T | -0.111 | 0.023 | 2.12E-06 | -0.006 | 0.052 | 0.91 | |
| Erysipelatoclostridium | rs61806970 | C | T | 0.143 | 0.032 | 8.79E-06 | -0.065 | 0.066 | 0.33 | |
| Erysipelatoclostridium | rs622418 | A | G | -0.067 | 0.014 | 3.10E-06 | 0.032 | 0.029 | 0.27 | |
| Erysipelatoclostridium | rs6474512 | A | C | 0.067 | 0.014 | 2.87E-06 | 0.024 | 0.029 | 0.42 | |
| Erysipelatoclostridium | rs710230 | T | C | 0.143 | 0.028 | 3.52E-07 | 0.070 | 0.062 | 0.25 | |
| Erysipelatoclostridium | rs7221249 | A | G | 0.084 | 0.014 | 4.01E-09 | 0.018 | 0.030 | 0.54 | |
| Erysipelatoclostridium | rs9590927 | G | A | -0.065 | 0.014 | 6.72E-06 | 0.002 | 0.029 | 0.93 | |
| *Erysipelotrichaceae (UCG003)* | rs11666127 | A | G | -0.072 | 0.016 | 8.32E-06 | -0.001 | 0.041 | 0.98 | |
| *Erysipelotrichaceae (UCG003)* | rs11877693 | C | G | -0.095 | 0.019 | 5.39E-07 | -0.028 | 0.048 | 0.56 | |
| *Erysipelotrichaceae (UCG003)* | rs11994308 | C | T | 0.115 | 0.024 | 1.94E-06 | -0.053 | 0.061 | 0.38 | |
| *Erysipelotrichaceae (UCG003)* | rs12251396 | A | G | -0.071 | 0.016 | 8.87E-06 | 0.058 | 0.038 | 0.13 | |
| *Erysipelotrichaceae (UCG003)* | rs17798136 | G | A | 0.159 | 0.035 | 4.92E-06 | 0.064 | 0.080 | 0.43 | |
| *Erysipelotrichaceae (UCG003)* | rs2044550 | T | C | -0.061 | 0.014 | 7.50E-06 | 0.031 | 0.034 | 0.37 | |
| *Erysipelotrichaceae (UCG003)* | rs28568391 | A | G | -0.058 | 0.012 | 8.76E-07 | -0.044 | 0.034 | 0.21 | |
| *Erysipelotrichaceae (UCG003)* | rs4758231 | G | T | -0.055 | 0.012 | 6.16E-06 | -0.025 | 0.032 | 0.43 | |
| *Erysipelotrichaceae (UCG003)* | rs59068084 | T | G | 0.056 | 0.012 | 2.63E-06 | 0.002 | 0.030 | 0.94 | |
| *Erysipelotrichaceae (UCG003)* | rs62403464 | T | C | -0.073 | 0.016 | 2.94E-06 | -0.057 | 0.039 | 0.15 | |
| *Erysipelotrichaceae (UCG003)* | rs6875357 | C | T | 0.166 | 0.035 | 2.84E-06 | 0.100 | 0.070 | 0.15 | |
| *Erysipelotrichaceae (UCG003)* | rs75949021 | T | C | -0.170 | 0.037 | 5.80E-06 | -0.023 | 0.065 | 0.73 | |
| *Erysipelotrichaceae (UCG003)* | rs76502207 | T | C | 0.145 | 0.029 | 5.86E-07 | 0.126 | 0.072 | 0.08 | |
| *Erysipelotrichaceae (UCG003)* | rs76544074 | G | C | -0.121 | 0.027 | 7.65E-06 | 0.000 | 0.061 | 0.99 | |
| *Erysipelotrichaceae (UCG003)* | rs79396538 | C | G | 0.085 | 0.019 | 9.12E-06 | 0.041 | 0.046 | 0.37 | |
| *Erysipelotrichaceae (UCG003)* | rs8053479 | A | G | -0.084 | 0.019 | 6.98E-06 | 0.009 | 0.050 | 0.86 | |
| *Escherichia Shigella* | rs112767262 | T | C | 0.073 | 0.016 | 7.45E-06 | 0.048 | 0.039 | 0.21 | |
| *Escherichia Shigella* | rs113127095 | A | G | 0.151 | 0.032 | 3.03E-06 | -0.109 | 0.069 | 0.11 | |
| *Escherichia Shigella* | rs113513883 | A | G | 0.172 | 0.038 | 5.93E-06 | 0.015 | 0.079 | 0.85 | |
| *Escherichia Shigella* | rs1154904 | A | G | -0.061 | 0.013 | 2.67E-06 | 0.014 | 0.029 | 0.63 | |
| *Escherichia Shigella* | rs11706043 | T | A | 0.076 | 0.016 | 4.05E-06 | 0.039 | 0.039 | 0.31 | |
| *Escherichia Shigella* | rs117092367 | A | T | 0.117 | 0.026 | 9.14E-06 | 0.036 | 0.069 | 0.60 | |
| *Escherichia Shigella* | rs2267739 | G | C | 0.116 | 0.024 | 1.17E-06 | -0.023 | 0.064 | 0.73 | |
| *Escherichia Shigella* | rs2798105 | A | G | -0.101 | 0.022 | 5.57E-06 | -0.036 | 0.049 | 0.46 | |
| *Escherichia Shigella* | rs35555519 | C | G | 0.102 | 0.022 | 4.76E-06 | 0.070 | 0.056 | 0.22 | |
| *Escherichia Shigella* | rs4731451 | G | A | -0.061 | 0.014 | 6.41E-06 | 0.021 | 0.031 | 0.48 | |
| *Escherichia Shigella* | rs57024273 | T | C | 0.063 | 0.014 | 7.63E-06 | -0.021 | 0.033 | 0.54 | |
| *Escherichia Shigella* | rs592299 | T | C | -0.059 | 0.013 | 4.83E-06 | 0.003 | 0.030 | 0.91 | |
| *Escherichia Shigella* | rs73208162 | A | G | -0.119 | 0.025 | 1.56E-06 | 0.035 | 0.063 | 0.57 | |
| *Escherichia Shigella* | rs7502686 | G | C | -0.136 | 0.030 | 6.56E-06 | -0.076 | 0.066 | 0.25 | |
| *Escherichia Shigella* | rs76141197 | C | T | 0.132 | 0.029 | 6.18E-06 | 0.129 | 0.068 | 0.06 | |
| *Escherichia Shigella* | rs78143293 | A | G | -0.080 | 0.018 | 9.57E-06 | -0.043 | 0.042 | 0.31 | |
| *Eubacterium (brachy group)* | rs1108552 | G | T | -0.111 | 0.025 | 7.96E-06 | 0.003 | 0.033 | 0.93 | |
| *Eubacterium (brachy group)* | rs12151423 | A | G | 0.101 | 0.023 | 8.29E-06 | 0.029 | 0.029 | 0.32 | |
| *Eubacterium (brachy group)* | rs13139592 | T | C | -0.146 | 0.033 | 8.12E-06 | -0.052 | 0.043 | 0.23 | |
| *Eubacterium (brachy group)* | rs1384962 | A | G | 0.121 | 0.027 | 5.62E-06 | -0.010 | 0.036 | 0.79 | |
| *Eubacterium (brachy group)* | rs2913110 | C | T | 0.105 | 0.023 | 4.58E-06 | -0.009 | 0.030 | 0.76 | |
| *Eubacterium (brachy group)* | rs4862235 | G | A | 0.105 | 0.023 | 3.44E-06 | 0.033 | 0.030 | 0.28 | |
| *Eubacterium (brachy group)* | rs55932844 | A | G | -0.171 | 0.036 | 2.26E-06 | -0.011 | 0.049 | 0.82 | |
| *Eubacterium (brachy group)* | rs62348779 | T | C | -0.201 | 0.043 | 3.25E-06 | -0.112 | 0.064 | 0.08 | |
| *Eubacterium (brachy group)* | rs6591893 | G | A | 0.108 | 0.024 | 6.69E-06 | 0.016 | 0.031 | 0.62 | |
| *Eubacterium (brachy group)* | rs720439 | A | G | -0.112 | 0.025 | 8.40E-06 | -0.037 | 0.034 | 0.27 | |
| *Eubacterium (brachy group)* | rs73199919 | T | C | -0.237 | 0.053 | 8.38E-06 | -0.027 | 0.076 | 0.72 | |
| *Eubacterium (brachy group)* | rs79847697 | A | G | -0.146 | 0.033 | 8.12E-06 | -0.006 | 0.040 | 0.88 | |
| *Eubacterium (brachy group)* | rs9554379 | A | G | -0.161 | 0.036 | 8.13E-06 | 0.029 | 0.051 | 0.58 | |
| *Eubacterium (brachy group)* | rs9613196 | T | A | -0.239 | 0.053 | 6.29E-06 | 0.116 | 0.067 | 0.08 | |
| *Eubacterium (coprostanoligenes group)* | rs1020520 | T | G | -0.059 | 0.013 | 8.84E-06 | 0.065 | 0.037 | 0.08 | |
| *Eubacterium (coprostanoligenes group)* | rs10217667 | C | T | -0.065 | 0.015 | 7.36E-06 | 0.113 | 0.046 | 0.01 | |
| *Eubacterium (coprostanoligenes group)* | rs10444197 | A | G | -0.051 | 0.011 | 8.26E-06 | -0.016 | 0.032 | 0.61 | |
| *Eubacterium (coprostanoligenes group)* | rs11052069 | T | C | 0.048 | 0.011 | 9.37E-06 | 0.040 | 0.031 | 0.19 | |
| *Eubacterium (coprostanoligenes group)* | rs11808093 | T | C | 0.079 | 0.017 | 5.39E-06 | 0.002 | 0.049 | 0.97 | |
| *Eubacterium (coprostanoligenes group)* | rs12906958 | C | T | -0.053 | 0.012 | 4.25E-06 | -0.056 | 0.031 | 0.07 | |
| *Eubacterium (coprostanoligenes group)* | rs17159861 | C | T | 0.096 | 0.017 | 1.10E-08 | -0.060 | 0.051 | 0.24 | |
| *Eubacterium (coprostanoligenes group)* | rs2644213 | G | A | 0.054 | 0.012 | 8.82E-06 | -0.015 | 0.034 | 0.67 | |
| *Eubacterium (coprostanoligenes group)* | rs4076415 | T | G | 0.052 | 0.011 | 3.00E-06 | 0.002 | 0.032 | 0.96 | |
| *Eubacterium (coprostanoligenes group)* | rs4717831 | A | T | 0.079 | 0.017 | 6.16E-06 | 0.014 | 0.063 | 0.82 | |
| *Eubacterium (coprostanoligenes group)* | rs62024432 | C | T | -0.077 | 0.017 | 7.74E-06 | -0.031 | 0.048 | 0.52 | |
| *Eubacterium (coprostanoligenes group)* | rs6762473 | C | A | 0.052 | 0.011 | 3.44E-06 | 0.019 | 0.030 | 0.53 | |
| *Eubacterium (coprostanoligenes group)* | rs76898927 | G | A | 0.123 | 0.027 | 3.84E-06 | 0.034 | 0.073 | 0.64 | |
| *Eubacterium (coprostanoligenes group)* | rs79895140 | T | C | -0.064 | 0.014 | 5.59E-06 | 0.095 | 0.041 | 0.02 | |
| *Eubacterium (coprostanoligenes group)* | rs9648214 | T | C | -0.083 | 0.016 | 4.57E-07 | 0.041 | 0.043 | 0.34 | |
| *Eubacterium (eligens group)* | rs113154224 | G | A | 0.101 | 0.023 | 9.22E-06 | 0.004 | 0.057 | 0.95 | |
| *Eubacterium (eligens group)* | rs158115 | G | C | 0.092 | 0.019 | 1.57E-06 | -0.127 | 0.052 | 0.02 | |
| *Eubacterium (eligens group)* | rs2200429 | A | G | -0.089 | 0.020 | 7.53E-06 | -0.015 | 0.052 | 0.78 | |
| *Eubacterium (eligens group)* | rs265534 | T | G | -0.056 | 0.012 | 2.69E-06 | -0.033 | 0.030 | 0.27 | |
| *Eubacterium (eligens group)* | rs4583233 | A | C | 0.067 | 0.013 | 1.69E-07 | -0.060 | 0.033 | 0.07 | |
| *Eubacterium (eligens group)* | rs6923695 | T | G | 0.103 | 0.023 | 6.82E-06 | 0.077 | 0.054 | 0.16 | |
| *Eubacterium (eligens group)* | rs74606150 | C | G | -0.196 | 0.043 | 3.92E-06 | 0.098 | 0.076 | 0.20 | |
| *Eubacterium (eligens group)* | rs76174568 | T | A | -0.080 | 0.018 | 9.20E-06 | -0.005 | 0.043 | 0.91 | |
| *Eubacterium (fissicatena group)* | rs11818408 | G | A | 0.106 | 0.024 | 8.04E-06 | -0.019 | 0.030 | 0.52 | |
| *Eubacterium (fissicatena group)* | rs11876297 | T | C | 0.131 | 0.028 | 3.06E-06 | -0.034 | 0.037 | 0.35 | |
| *Eubacterium (fissicatena group)* | rs151257695 | A | G | 0.210 | 0.045 | 4.10E-06 | -0.032 | 0.062 | 0.60 | |
| *Eubacterium (fissicatena group)* | rs1764136 | G | C | 0.136 | 0.031 | 8.37E-06 | 0.008 | 0.039 | 0.84 | |
| *Eubacterium (fissicatena group)* | rs2733072 | G | A | 0.110 | 0.023 | 1.57E-06 | -0.042 | 0.031 | 0.17 | |
| *Eubacterium (fissicatena group)* | rs3771393 | C | T | 0.131 | 0.027 | 9.27E-07 | -0.004 | 0.033 | 0.91 | |
| *Eubacterium (fissicatena group)* | rs7104872 | G | A | 0.139 | 0.029 | 2.05E-06 | 0.028 | 0.038 | 0.47 | |
| *Eubacterium (hallii group)* | rs10501370 | C | T | -0.116 | 0.025 | 4.69E-06 | -0.048 | 0.077 | 0.53 | |
| *Eubacterium (hallii group)* | rs10798999 | C | T | 0.060 | 0.013 | 2.07E-06 | 0.026 | 0.036 | 0.46 | |
| *Eubacterium (hallii group)* | rs10808115 | A | C | -0.050 | 0.011 | 4.39E-06 | 0.048 | 0.029 | 0.10 | |
| *Eubacterium (hallii group)* | rs13116360 | T | C | 0.154 | 0.030 | 2.15E-07 | 0.102 | 0.075 | 0.18 | |
| *Eubacterium (hallii group)* | rs281379 | A | G | -0.050 | 0.011 | 8.43E-06 | -0.024 | 0.029 | 0.42 | |
| *Eubacterium (hallii group)* | rs28584818 | A | G | 0.126 | 0.027 | 2.67E-06 | -0.043 | 0.069 | 0.54 | |
| *Eubacterium (hallii group)* | rs60254196 | A | G | -0.052 | 0.011 | 2.96E-06 | 0.010 | 0.031 | 0.74 | |
| *Eubacterium (hallii group)* | rs630939 | C | T | -0.051 | 0.011 | 8.57E-06 | 0.018 | 0.031 | 0.57 | |
| *Eubacterium (hallii group)* | rs6550770 | T | C | -0.198 | 0.044 | 7.97E-06 | 0.106 | 0.078 | 0.17 | |
| *Eubacterium (hallii group)* | rs74018587 | C | T | 0.209 | 0.044 | 1.86E-06 | -0.049 | 0.083 | 0.55 | |
| *Eubacterium (hallii group)* | rs78056098 | G | T | -0.051 | 0.011 | 8.18E-06 | 0.048 | 0.030 | 0.11 | |
| *Eubacterium (hallii group)* | rs949971 | T | G | -0.054 | 0.012 | 3.28E-06 | -0.035 | 0.030 | 0.25 | |
| *Eubacterium (nodatum group)* | rs10458299 | T | C | -0.188 | 0.042 | 7.69E-06 | 0.023 | 0.054 | 0.67 | |
| *Eubacterium (nodatum group)* | rs11006576 | A | G | -0.110 | 0.025 | 7.48E-06 | 0.025 | 0.031 | 0.42 | |
| *Eubacterium (nodatum group)* | rs113893692 | C | T | -0.185 | 0.040 | 4.50E-06 | -0.064 | 0.048 | 0.19 | |
| *Eubacterium (nodatum group)* | rs13069706 | A | C | -0.109 | 0.025 | 9.96E-06 | 0.040 | 0.029 | 0.17 | |
| *Eubacterium (nodatum group)* | rs34297067 | A | G | -0.187 | 0.034 | 4.41E-08 | 0.056 | 0.041 | 0.17 | |
| *Eubacterium (nodatum group)* | rs61841040 | G | T | 0.161 | 0.034 | 2.58E-06 | 0.025 | 0.042 | 0.55 | |
| *Eubacterium (nodatum group)* | rs6818880 | A | G | -0.110 | 0.025 | 7.62E-06 | 0.061 | 0.030 | 0.04 | |
| *Eubacterium (nodatum group)* | rs77910827 | C | T | 0.202 | 0.041 | 1.07E-06 | -0.038 | 0.049 | 0.43 | |
| *Eubacterium (nodatum group)* | rs7827125 | C | T | 0.122 | 0.027 | 6.56E-06 | 0.036 | 0.032 | 0.27 | |
| *Eubacterium (nodatum group)* | rs7880204 | T | C | -0.125 | 0.028 | 5.19E-06 | 0.020 | 0.033 | 0.54 | |
| *Eubacterium (nodatum group)* | rs9425984 | T | C | -0.130 | 0.029 | 8.40E-06 | 0.004 | 0.035 | 0.91 | |
| *Eubacterium (nodatum group)* | rs9532151 | A | G | -0.110 | 0.025 | 9.88E-06 | 0.029 | 0.029 | 0.33 | |
| *Eubacterium (oxidoreducens group)* | rs12129908 | C | A | 0.089 | 0.020 | 6.67E-06 | -0.006 | 0.030 | 0.84 | |
| *Eubacterium (oxidoreducens group)* | rs12326318 | A | C | 0.149 | 0.033 | 8.11E-06 | 0.018 | 0.058 | 0.76 | |
| *Eubacterium (oxidoreducens group)* | rs12423772 | G | T | 0.141 | 0.030 | 1.77E-06 | -0.058 | 0.051 | 0.25 | |
| *Eubacterium (oxidoreducens group)* | rs1425962 | G | C | 0.091 | 0.020 | 6.28E-06 | -0.006 | 0.032 | 0.85 | |
| *Eubacterium (oxidoreducens group)* | rs2647695 | G | A | 0.112 | 0.025 | 7.30E-06 | 0.029 | 0.037 | 0.43 | |
| *Eubacterium (oxidoreducens group)* | rs2973294 | G | T | 0.092 | 0.020 | 2.29E-06 | -0.014 | 0.029 | 0.63 | |
| *Eubacterium (oxidoreducens group)* | rs34561138 | G | A | 0.216 | 0.046 | 2.61E-06 | -0.037 | 0.063 | 0.56 | |
| *Eubacterium (oxidoreducens group)* | rs440215 | C | T | 0.093 | 0.020 | 1.79E-06 | 0.015 | 0.029 | 0.61 | |
| *Eubacterium (oxidoreducens group)* | rs61884310 | C | T | 0.117 | 0.026 | 9.29E-06 | -0.018 | 0.041 | 0.67 | |
| *Eubacterium (rectale group)* | rs10248854 | C | A | -0.053 | 0.011 | 3.29E-06 | 0.026 | 0.030 | 0.39 | |
| *Eubacterium (rectale group)* | rs10797540 | A | G | 0.050 | 0.011 | 3.45E-06 | -0.022 | 0.029 | 0.46 | |
| *Eubacterium (rectale group)* | rs10892089 | C | G | -0.064 | 0.014 | 5.70E-06 | 0.055 | 0.037 | 0.14 | |
| *Eubacterium (rectale group)* | rs112687713 | A | T | -0.205 | 0.046 | 9.54E-06 | -0.048 | 0.107 | 0.65 | |
| *Eubacterium (rectale group)* | rs117151453 | G | C | -0.113 | 0.024 | 3.39E-06 | -0.008 | 0.065 | 0.90 | |
| *Eubacterium (rectale group)* | rs16960159 | C | G | -0.157 | 0.034 | 3.20E-06 | 0.036 | 0.068 | 0.60 | |
| *Eubacterium (rectale group)* | rs2884897 | A | G | -0.129 | 0.029 | 7.61E-06 | 0.072 | 0.072 | 0.32 | |
| *Eubacterium (rectale group)* | rs314726 | T | C | 0.053 | 0.011 | 1.37E-06 | -0.015 | 0.030 | 0.63 | |
| *Eubacterium (rectale group)* | rs35398954 | A | G | -0.090 | 0.017 | 2.44E-07 | 0.083 | 0.050 | 0.10 | |
| *Eubacterium (rectale group)* | rs35953943 | G | A | 0.099 | 0.022 | 8.68E-06 | -0.044 | 0.061 | 0.47 | |
| *Eubacterium (rectale group)* | rs3980709 | A | T | -0.062 | 0.014 | 9.43E-06 | -0.043 | 0.039 | 0.27 | |
| *Eubacterium (rectale group)* | rs62547233 | A | G | 0.054 | 0.012 | 8.12E-06 | -0.006 | 0.033 | 0.86 | |
| *Eubacterium (ruminantium group)* | rs10131724 | A | C | -0.200 | 0.041 | 1.43E-06 | -0.023 | 0.067 | 0.73 | |
| *Eubacterium (ruminantium group)* | rs10923018 | G | A | 0.073 | 0.016 | 6.36E-06 | -0.015 | 0.029 | 0.62 | |
| *Eubacterium (ruminantium group)* | rs11637981 | G | T | -0.073 | 0.016 | 5.28E-06 | -0.047 | 0.030 | 0.11 | |
| *Eubacterium (ruminantium group)* | rs13025464 | T | C | -0.074 | 0.016 | 6.79E-06 | -0.018 | 0.030 | 0.55 | |
| *Eubacterium (ruminantium group)* | rs139749 | C | T | -0.085 | 0.017 | 8.60E-07 | -0.014 | 0.031 | 0.66 | |
| *Eubacterium (ruminantium group)* | rs16891896 | G | A | -0.175 | 0.039 | 7.64E-06 | -0.048 | 0.067 | 0.48 | |
| *Eubacterium (ruminantium group)* | rs17519472 | C | T | 0.108 | 0.023 | 4.08E-06 | -0.038 | 0.043 | 0.38 | |
| *Eubacterium (ruminantium group)* | rs2116427 | A | G | 0.091 | 0.018 | 5.78E-07 | -0.055 | 0.034 | 0.10 | |
| *Eubacterium (ruminantium group)* | rs2229917 | A | G | 0.154 | 0.032 | 2.14E-06 | 0.031 | 0.066 | 0.64 | |
| *Eubacterium (ruminantium group)* | rs2418654 | C | T | -0.075 | 0.017 | 6.32E-06 | 0.044 | 0.033 | 0.18 | |
| *Eubacterium (ruminantium group)* | rs2817174 | C | T | -0.073 | 0.016 | 7.26E-06 | 0.036 | 0.030 | 0.23 | |
| *Eubacterium (ruminantium group)* | rs57340348 | T | C | -0.098 | 0.021 | 3.91E-06 | 0.050 | 0.038 | 0.19 | |
| *Eubacterium (ruminantium group)* | rs606117 | A | G | 0.083 | 0.018 | 3.94E-06 | -0.006 | 0.032 | 0.84 | |
| *Eubacterium (ruminantium group)* | rs6676699 | G | T | -0.089 | 0.020 | 6.16E-06 | 0.013 | 0.036 | 0.71 | |
| *Eubacterium (ruminantium group)* | rs7000472 | A | G | -0.076 | 0.017 | 3.96E-06 | -0.041 | 0.030 | 0.17 | |
| *Eubacterium (ruminantium group)* | rs72836424 | C | T | -0.140 | 0.030 | 3.32E-06 | 0.016 | 0.052 | 0.75 | |
| *Eubacterium (ruminantium group)* | rs73139629 | A | C | -0.115 | 0.025 | 3.44E-06 | 0.009 | 0.046 | 0.84 | |
| *Eubacterium (ruminantium group)* | rs76147458 | T | C | -0.120 | 0.027 | 9.42E-06 | 0.052 | 0.048 | 0.28 | |
| *Eubacterium (ventriosum group)* | rs11617697 | A | G | -0.143 | 0.029 | 5.50E-07 | -0.052 | 0.073 | 0.47 | |
| *Eubacterium (ventriosum group)* | rs12964517 | G | A | 0.059 | 0.012 | 1.97E-06 | -0.004 | 0.031 | 0.89 | |
| *Eubacterium (ventriosum group)* | rs13082419 | C | T | -0.072 | 0.016 | 9.25E-06 | -0.003 | 0.039 | 0.95 | |
| *Eubacterium (ventriosum group)* | rs16884680 | G | T | -0.091 | 0.019 | 2.32E-06 | -0.089 | 0.059 | 0.13 | |
| *Eubacterium (ventriosum group)* | rs35179274 | C | T | -0.063 | 0.014 | 5.36E-06 | 0.028 | 0.034 | 0.41 | |
| *Eubacterium (ventriosum group)* | rs3809430 | T | C | -0.055 | 0.012 | 3.68E-06 | -0.002 | 0.030 | 0.95 | |
| *Eubacterium (ventriosum group)* | rs4725006 | G | A | -0.071 | 0.016 | 6.92E-06 | 0.114 | 0.041 | 0.01 | |
| *Eubacterium (ventriosum group)* | rs57199565 | T | C | 0.078 | 0.016 | 1.02E-06 | -0.007 | 0.049 | 0.89 | |
| *Eubacterium (ventriosum group)* | rs6048195 | A | T | -0.060 | 0.012 | 2.25E-07 | -0.034 | 0.039 | 0.38 | |
| *Eubacterium (ventriosum group)* | rs66746423 | C | T | 0.075 | 0.016 | 5.23E-06 | 0.047 | 0.042 | 0.26 | |
| *Eubacterium (ventriosum group)* | rs66830358 | T | A | 0.053 | 0.012 | 7.39E-06 | -0.004 | 0.031 | 0.90 | |
| *Eubacterium (ventriosum group)* | rs6704822 | A | G | 0.074 | 0.017 | 9.25E-06 | -0.112 | 0.042 | 0.01 | |
| *Eubacterium (ventriosum group)* | rs6791216 | C | T | -0.051 | 0.012 | 9.85E-06 | 0.025 | 0.030 | 0.40 | |
| *Eubacterium (ventriosum group)* | rs72783037 | C | A | 0.066 | 0.014 | 4.56E-06 | 0.026 | 0.036 | 0.46 | |
| *Eubacterium (ventriosum group)* | rs73615400 | T | C | -0.096 | 0.019 | 7.65E-07 | 0.008 | 0.052 | 0.88 | |
| *Eubacterium (ventriosum group)* | rs78250280 | G | A | 0.075 | 0.016 | 5.10E-06 | 0.106 | 0.047 | 0.03 | |
| *Eubacterium (ventriosum group)* | rs876734 | C | T | -0.062 | 0.013 | 3.04E-06 | -0.064 | 0.036 | 0.07 | |
| *Eubacterium (ventriosum group)* | rs9316536 | T | G | -0.082 | 0.018 | 8.11E-06 | 0.011 | 0.054 | 0.83 | |
| *Eubacterium (xylanophilum group)* | rs10140184 | A | C | 0.058 | 0.013 | 4.69E-06 | 0.008 | 0.035 | 0.82 | |
| *Eubacterium (xylanophilum group)* | rs10917203 | A | C | 0.061 | 0.013 | 2.87E-06 | -0.051 | 0.033 | 0.12 | |
| *Eubacterium (xylanophilum group)* | rs112176119 | C | T | -0.113 | 0.025 | 3.90E-06 | -0.002 | 0.063 | 0.98 | |
| *Eubacterium (xylanophilum group)* | rs12980122 | C | G | -0.108 | 0.024 | 5.51E-06 | -0.013 | 0.061 | 0.83 | |
| *Eubacterium (xylanophilum group)* | rs13239072 | G | A | 0.069 | 0.014 | 1.46E-06 | -0.028 | 0.034 | 0.41 | |
| *Eubacterium (xylanophilum group)* | rs17830032 | G | A | -0.161 | 0.031 | 2.32E-07 | 0.014 | 0.069 | 0.84 | |
| *Eubacterium (xylanophilum group)* | rs1999224 | G | T | -0.095 | 0.020 | 3.11E-06 | 0.015 | 0.049 | 0.76 | |
| *Eubacterium (xylanophilum group)* | rs2012708 | A | G | 0.057 | 0.013 | 6.28E-06 | 0.028 | 0.031 | 0.36 | |
| *Eubacterium (xylanophilum group)* | rs2213117 | T | G | 0.088 | 0.019 | 3.42E-06 | 0.098 | 0.050 | 0.05 | |
| *Eubacterium (xylanophilum group)* | rs4654122 | C | G | 0.055 | 0.012 | 7.51E-06 | 0.020 | 0.029 | 0.49 | |
| *Eubacterium (xylanophilum group)* | rs7847151 | A | G | -0.092 | 0.021 | 7.52E-06 | -0.045 | 0.049 | 0.36 | |
| *Eubacterium (xylanophilum group)* | rs79582700 | C | G | -0.095 | 0.020 | 1.94E-06 | 0.059 | 0.047 | 0.21 | |
| *Faecalibacterium* | rs10927394 | G | T | -0.232 | 0.051 | 5.81E-06 | 0.011 | 0.088 | 0.90 | |
| *Faecalibacterium* | rs114946999 | C | T | -0.086 | 0.019 | 5.52E-06 | -0.022 | 0.053 | 0.68 | |
| *Faecalibacterium* | rs11776390 | T | C | -0.078 | 0.017 | 5.12E-06 | -0.093 | 0.052 | 0.07 | |
| *Faecalibacterium* | rs12320842 | C | G | 0.095 | 0.016 | 7.30E-09 | -0.031 | 0.047 | 0.51 | |
| *Faecalibacterium* | rs1271565 | C | T | -0.058 | 0.012 | 1.46E-06 | 0.041 | 0.038 | 0.28 | |
| *Faecalibacterium* | rs149783253 | C | G | 0.161 | 0.035 | 4.95E-06 | 0.012 | 0.072 | 0.87 | |
| *Faecalibacterium* | rs28376661 | C | G | 0.050 | 0.011 | 3.92E-06 | -0.025 | 0.030 | 0.40 | |
| *Faecalibacterium* | rs61875484 | C | G | 0.082 | 0.018 | 8.47E-06 | 0.020 | 0.054 | 0.72 | |
| *Faecalibacterium* | rs6910935 | A | G | 0.135 | 0.028 | 1.13E-06 | 0.027 | 0.068 | 0.69 | |
| *Faecalibacterium* | rs75499067 | C | T | 0.228 | 0.047 | 1.01E-06 | 0.041 | 0.073 | 0.57 | |
| *Faecalibacterium* | rs79656633 | T | C | 0.146 | 0.032 | 6.53E-06 | 0.130 | 0.076 | 0.09 | |
| *Faecalibacterium* | rs9536330 | T | C | -0.048 | 0.011 | 7.64E-06 | 0.054 | 0.030 | 0.07 | |
| *Family XIIIAD (3011 group)* | rs11126423 | C | T | 0.090 | 0.020 | 4.08E-06 | -0.013 | 0.055 | 0.81 | |
| *Family XIIIAD (3011 group)* | rs12314465 | A | G | -0.092 | 0.020 | 3.10E-06 | -0.009 | 0.048 | 0.85 | |
| *Family XIIIAD (3011 group)* | rs12911842 | A | T | -0.081 | 0.018 | 9.47E-06 | -0.046 | 0.045 | 0.30 | |
| *Family XIIIAD (3011 group)* | rs13131489 | A | G | 0.060 | 0.013 | 7.90E-06 | 0.012 | 0.033 | 0.72 | |
| *Family XIIIAD (3011 group)* | rs149302 | T | C | -0.065 | 0.014 | 6.55E-06 | 0.015 | 0.036 | 0.67 | |
| *Family XIIIAD (3011 group)* | rs16840310 | A | G | -0.061 | 0.012 | 6.43E-07 | -0.008 | 0.030 | 0.80 | |
| *Family XIIIAD (3011 group)* | rs16940167 | C | T | 0.073 | 0.016 | 4.62E-06 | -0.017 | 0.040 | 0.67 | |
| *Family XIIIAD (3011 group)* | rs17156849 | G | A | -0.113 | 0.025 | 4.18E-06 | 0.064 | 0.058 | 0.27 | |
| *Family XIIIAD (3011 group)* | rs62200412 | C | T | -0.080 | 0.016 | 1.02E-06 | -0.031 | 0.043 | 0.48 | |
| *Family XIIIAD (3011 group)* | rs72730932 | C | A | -0.090 | 0.018 | 3.79E-07 | -0.083 | 0.042 | 0.05 | |
| *Family XIIIAD (3011 group)* | rs7449021 | A | G | 0.060 | 0.013 | 7.31E-06 | 0.005 | 0.033 | 0.87 | |
| *Family XIIIAD (3011 group)* | rs9837139 | A | G | 0.108 | 0.024 | 7.78E-06 | -0.089 | 0.061 | 0.15 | |
| *Family XIIIAD (3011 group)* | rs9852893 | C | G | 0.066 | 0.013 | 3.45E-07 | 0.033 | 0.035 | 0.34 | |
| *Family XIII (UCG001)* | rs111936637 | G | A | -0.133 | 0.030 | 9.82E-06 | -0.106 | 0.070 | 0.13 | |
| *Family XIII (UCG001)* | rs112362903 | A | G | -0.149 | 0.033 | 7.74E-06 | 0.056 | 0.076 | 0.46 | |
| *Family XIII (UCG001)* | rs116979587 | T | A | -0.122 | 0.026 | 3.08E-06 | 0.159 | 0.072 | 0.03 | |
| *Family XIII (UCG001)* | rs12049454 | T | C | -0.065 | 0.013 | 1.38E-06 | -0.089 | 0.032 | 0.01 | |
| *Family XIII (UCG001)* | rs1426266 | T | C | -0.067 | 0.014 | 1.22E-06 | -0.011 | 0.033 | 0.74 | |
| *Family XIII (UCG001)* | rs2276529 | C | G | -0.076 | 0.017 | 4.23E-06 | -0.006 | 0.043 | 0.88 | |
| *Family XIII (UCG001)* | rs3842897 | G | A | -0.113 | 0.024 | 3.48E-06 | 0.001 | 0.065 | 0.98 | |
| *Family XIII (UCG001)* | rs39790 | T | A | -0.085 | 0.019 | 5.48E-06 | 0.045 | 0.046 | 0.33 | |
| *Family XIII (UCG001)* | rs62414802 | C | T | -0.061 | 0.013 | 5.44E-06 | 0.013 | 0.032 | 0.68 | |
| *Family XIII (UCG001)* | rs7119679 | G | A | -0.081 | 0.017 | 3.67E-06 | 0.002 | 0.047 | 0.97 | |
| *Family XIII (UCG001)* | rs7549877 | C | T | 0.103 | 0.023 | 6.78E-06 | -0.009 | 0.055 | 0.88 | |
| *Family XIII (UCG001)* | rs76463770 | A | G | 0.193 | 0.042 | 4.23E-06 | -0.069 | 0.069 | 0.31 | |
| *Family XIII (UCG001)* | rs8076666 | A | G | 0.089 | 0.020 | 7.63E-06 | -0.063 | 0.053 | 0.24 | |
| *Flavonifractor* | rs114873521 | C | T | -0.130 | 0.029 | 9.75E-06 | 0.098 | 0.065 | 0.13 | |
| *Flavonifractor* | rs115199579 | A | G | -0.097 | 0.022 | 8.92E-06 | 0.006 | 0.046 | 0.90 | |
| *Flavonifractor* | rs11642826 | G | C | 0.147 | 0.033 | 6.30E-06 | -0.064 | 0.068 | 0.35 | |
| *Flavonifractor* | rs11811696 | T | C | -0.116 | 0.024 | 1.47E-06 | -0.050 | 0.053 | 0.35 | |
| *Flavonifractor* | rs12030302 | A | G | -0.069 | 0.014 | 4.76E-07 | 0.019 | 0.029 | 0.50 | |
| *Flavonifractor* | rs12038887 | C | G | 0.094 | 0.021 | 8.42E-06 | -0.068 | 0.052 | 0.19 | |
| *Flavonifractor* | rs34066017 | A | G | 0.076 | 0.016 | 1.73E-06 | -0.054 | 0.033 | 0.10 | |
| *Flavonifractor* | rs6761463 | G | C | -0.083 | 0.018 | 6.26E-06 | -0.008 | 0.039 | 0.84 | |
| *Flavonifractor* | rs806808 | T | C | 0.067 | 0.014 | 1.03E-06 | 0.001 | 0.029 | 0.98 | |
| *Fusicatenibacter* | rs10812048 | G | A | -0.072 | 0.016 | 8.02E-06 | -0.041 | 0.045 | 0.37 | |
| *Fusicatenibacter* | rs12042099 | T | C | -0.071 | 0.016 | 9.19E-06 | -0.025 | 0.045 | 0.58 | |
| *Fusicatenibacter* | rs1435397 | G | A | -0.059 | 0.013 | 8.90E-06 | -0.026 | 0.035 | 0.46 | |
| *Fusicatenibacter* | rs167879 | C | T | -0.066 | 0.015 | 9.27E-06 | 0.028 | 0.038 | 0.46 | |
| *Fusicatenibacter* | rs16866708 | G | A | -0.070 | 0.016 | 6.34E-06 | -0.062 | 0.040 | 0.12 | |
| *Fusicatenibacter* | rs1864685 | A | C | -0.049 | 0.011 | 4.72E-06 | -0.055 | 0.029 | 0.06 | |
| *Fusicatenibacter* | rs2025938 | G | A | -0.097 | 0.021 | 2.49E-06 | 0.013 | 0.055 | 0.82 | |
| *Fusicatenibacter* | rs2039204 | T | A | -0.050 | 0.011 | 4.06E-06 | 0.006 | 0.029 | 0.83 | |
| *Fusicatenibacter* | rs206581 | A | G | -0.057 | 0.013 | 8.84E-06 | 0.009 | 0.037 | 0.81 | |
| *Fusicatenibacter* | rs2132128 | G | A | -0.077 | 0.016 | 1.48E-06 | 0.046 | 0.045 | 0.31 | |
| *Fusicatenibacter* | rs227237 | A | G | 0.068 | 0.015 | 8.59E-06 | -0.070 | 0.044 | 0.11 | |
| *Fusicatenibacter* | rs3303 | T | C | -0.095 | 0.020 | 2.97E-06 | 0.014 | 0.060 | 0.82 | |
| *Fusicatenibacter* | rs4378146 | A | C | -0.062 | 0.013 | 8.51E-07 | 0.069 | 0.034 | 0.04 | |
| *Fusicatenibacter* | rs4671621 | C | T | -0.086 | 0.019 | 7.26E-06 | -0.083 | 0.062 | 0.18 | |
| *Fusicatenibacter* | rs57871170 | A | G | -0.128 | 0.028 | 5.18E-06 | -0.064 | 0.066 | 0.34 | |
| *Fusicatenibacter* | rs60254196 | A | G | -0.049 | 0.011 | 6.71E-06 | 0.010 | 0.031 | 0.74 | |
| *Fusicatenibacter* | rs62353480 | A | G | -0.070 | 0.015 | 1.45E-06 | 0.028 | 0.041 | 0.49 | |
| *Fusicatenibacter* | rs6515626 | G | A | 0.142 | 0.031 | 6.33E-06 | 0.035 | 0.077 | 0.65 | |
| *Fusicatenibacter* | rs704418 | T | C | 0.074 | 0.015 | 9.96E-07 | 0.017 | 0.046 | 0.72 | |
| *Fusicatenibacter* | rs7152703 | G | T | -0.148 | 0.033 | 7.85E-06 | -0.132 | 0.080 | 0.10 | |
| *Fusicatenibacter* | rs73103914 | A | G | -0.060 | 0.013 | 8.89E-06 | 0.002 | 0.036 | 0.95 | |
| *Fusicatenibacter* | rs792108 | T | C | -0.051 | 0.011 | 8.00E-06 | 0.015 | 0.032 | 0.64 | |
| *Fusicatenibacter* | rs8063430 | T | C | -0.104 | 0.022 | 2.84E-06 | -0.051 | 0.071 | 0.47 | |
| *Fusicatenibacter* | rs9905659 | G | A | -0.062 | 0.014 | 6.44E-06 | 0.037 | 0.038 | 0.33 | |
| *Gordonibacter* | rs117347059 | G | C | -0.128 | 0.029 | 6.85E-06 | -0.014 | 0.038 | 0.71 | |
| *Gordonibacter* | rs12139866 | A | G | 0.195 | 0.044 | 9.92E-06 | -0.026 | 0.060 | 0.67 | |
| *Gordonibacter* | rs13412653 | A | C | 0.108 | 0.024 | 6.87E-06 | -0.001 | 0.030 | 0.98 | |
| *Gordonibacter* | rs16955299 | G | A | -0.196 | 0.043 | 5.88E-06 | -0.047 | 0.059 | 0.43 | |
| *Gordonibacter* | rs322296 | G | A | 0.179 | 0.038 | 2.17E-06 | -0.029 | 0.048 | 0.54 | |
| *Gordonibacter* | rs35042269 | C | A | -0.180 | 0.040 | 7.80E-06 | -0.022 | 0.060 | 0.72 | |
| *Gordonibacter* | rs4596722 | A | G | 0.103 | 0.023 | 8.83E-06 | -0.015 | 0.029 | 0.61 | |
| *Gordonibacter* | rs61934597 | C | T | -0.172 | 0.039 | 9.03E-06 | -0.060 | 0.048 | 0.22 | |
| *Gordonibacter* | rs6795601 | A | G | 0.165 | 0.037 | 7.30E-06 | -0.064 | 0.050 | 0.20 | |
| *Gordonibacter* | rs71545975 | A | G | -0.154 | 0.034 | 5.54E-06 | 0.048 | 0.042 | 0.25 | |
| *Gordonibacter* | rs7220558 | A | T | 0.117 | 0.023 | 6.45E-07 | 0.016 | 0.030 | 0.60 | |
| *Gordonibacter* | rs72714787 | C | A | 0.181 | 0.038 | 1.51E-06 | -0.003 | 0.049 | 0.95 | |
| *Gordonibacter* | rs7294633 | C | T | 0.129 | 0.025 | 2.63E-07 | -0.013 | 0.033 | 0.68 | |
| *Gordonibacter* | rs76287110 | A | T | -0.243 | 0.047 | 1.87E-07 | 0.060 | 0.062 | 0.33 | |
| *Gordonibacter* | rs768830 | G | A | 0.150 | 0.033 | 6.93E-06 | 0.057 | 0.048 | 0.23 | |
| *Gordonibacter* | rs830825 | A | G | -0.133 | 0.030 | 7.95E-06 | -0.019 | 0.044 | 0.65 | |
| *Haemophilus* | rs10781340 | G | A | 0.095 | 0.020 | 3.02E-06 | -0.003 | 0.042 | 0.94 | |
| *Haemophilus* | rs10840326 | C | G | -0.068 | 0.015 | 7.74E-06 | 0.013 | 0.029 | 0.66 | |
| *Haemophilus* | rs111582866 | G | A | -0.124 | 0.026 | 1.78E-06 | 0.038 | 0.052 | 0.47 | |
| *Haemophilus* | rs11256467 | G | A | -0.138 | 0.031 | 6.00E-06 | -0.024 | 0.066 | 0.71 | |
| *Haemophilus* | rs12191680 | C | G | 0.107 | 0.020 | 1.05E-07 | -0.002 | 0.041 | 0.96 | |
| *Haemophilus* | rs12876183 | T | A | 0.075 | 0.017 | 7.35E-06 | 0.040 | 0.033 | 0.23 | |
| *Haemophilus* | rs35509 | G | A | 0.128 | 0.027 | 1.83E-06 | 0.048 | 0.051 | 0.34 | |
| *Haemophilus* | rs4822728 | T | C | 0.071 | 0.015 | 3.12E-06 | 0.016 | 0.031 | 0.60 | |
| *Haemophilus* | rs76022354 | C | T | 0.245 | 0.051 | 1.30E-06 | -0.032 | 0.073 | 0.66 | |
| *Haemophilus* | rs78909003 | T | C | -0.246 | 0.050 | 1.03E-06 | 0.007 | 0.070 | 0.92 | |
| *Haemophilus* | rs9328464 | T | C | 0.072 | 0.015 | 1.18E-06 | -0.019 | 0.029 | 0.51 | |
| *Haemophilus* | rs9382510 | C | T | -0.094 | 0.017 | 6.07E-08 | -0.081 | 0.035 | 0.02 | |
| *Haemophilus* | rs9574096 | A | T | -0.074 | 0.016 | 2.10E-06 | -0.023 | 0.030 | 0.45 | |
| *Haemophilus* | rs9895850 | T | C | -0.193 | 0.042 | 3.66E-06 | -0.037 | 0.066 | 0.57 | |
| *Holdemanella* | rs11747129 | C | T | -0.146 | 0.033 | 7.93E-06 | -0.096 | 0.067 | 0.16 | |
| *Holdemanella* | rs12513188 | G | A | 0.090 | 0.020 | 3.68E-06 | 0.022 | 0.038 | 0.56 | |
| *Holdemanella* | rs17586763 | T | C | -0.227 | 0.051 | 8.37E-06 | 0.063 | 0.073 | 0.39 | |
| *Holdemanella* | rs1830029 | C | G | -0.095 | 0.021 | 6.09E-06 | -0.054 | 0.041 | 0.19 | |
| *Holdemanella* | rs1926302 | G | A | -0.108 | 0.023 | 3.08E-06 | 0.035 | 0.047 | 0.46 | |
| *Holdemanella* | rs34187114 | C | A | -0.105 | 0.023 | 3.77E-06 | 0.039 | 0.040 | 0.33 | |
| *Holdemanella* | rs35228298 | G | A | 0.093 | 0.020 | 4.07E-06 | -0.044 | 0.037 | 0.23 | |
| *Holdemanella* | rs4541991 | T | C | -0.093 | 0.019 | 1.85E-06 | -0.065 | 0.036 | 0.07 | |
| *Holdemanella* | rs4802175 | G | A | 0.079 | 0.017 | 5.19E-06 | 0.055 | 0.042 | 0.18 | |
| *Holdemanella* | rs4990223 | T | C | -0.073 | 0.016 | 9.60E-06 | -0.006 | 0.030 | 0.84 | |
| *Holdemanella* | rs607782 | T | C | -0.085 | 0.017 | 7.36E-07 | 0.007 | 0.033 | 0.84 | |
| *Holdemanella* | rs62113381 | T | C | -0.105 | 0.023 | 5.50E-06 | 0.032 | 0.044 | 0.46 | |
| *Holdemanella* | rs73011279 | T | C | -0.096 | 0.020 | 1.40E-06 | -0.008 | 0.036 | 0.82 | |
| *Holdemanella* | rs75764681 | T | C | -0.283 | 0.060 | 2.29E-06 | -0.191 | 0.086 | 0.03 | |
| *Holdemanella* | rs761624 | C | G | 0.096 | 0.018 | 8.99E-08 | -0.002 | 0.033 | 0.95 | |
| *Holdemania* | rs10885477 | T | C | -0.135 | 0.030 | 7.60E-06 | 0.021 | 0.061 | 0.73 | |
| *Holdemania* | rs111745969 | A | G | 0.121 | 0.027 | 5.61E-06 | -0.017 | 0.052 | 0.75 | |
| *Holdemania* | rs112138427 | T | C | 0.102 | 0.023 | 8.14E-06 | -0.065 | 0.043 | 0.13 | |
| *Holdemania* | rs113593397 | A | G | -0.129 | 0.028 | 5.03E-06 | 0.006 | 0.053 | 0.90 | |
| *Holdemania* | rs116500994 | G | T | -0.138 | 0.029 | 2.75E-06 | 0.039 | 0.058 | 0.51 | |
| *Holdemania* | rs12701617 | A | G | -0.066 | 0.015 | 9.82E-06 | -0.006 | 0.029 | 0.83 | |
| *Holdemania* | rs150096134 | T | A | 0.162 | 0.033 | 1.05E-06 | 0.037 | 0.070 | 0.60 | |
| *Holdemania* | rs1867876 | T | C | 0.084 | 0.016 | 2.02E-07 | -0.031 | 0.034 | 0.36 | |
| *Holdemania* | rs41438744 | C | G | -0.125 | 0.027 | 3.79E-06 | -0.070 | 0.056 | 0.21 | |
| *Holdemania* | rs4146507 | C | T | 0.079 | 0.018 | 7.10E-06 | -0.011 | 0.035 | 0.76 | |
| *Holdemania* | rs4636956 | A | G | -0.067 | 0.015 | 8.84E-06 | 0.040 | 0.029 | 0.17 | |
| *Holdemania* | rs55888180 | C | G | 0.129 | 0.028 | 5.37E-06 | -0.029 | 0.057 | 0.61 | |
| *Holdemania* | rs6133067 | T | C | 0.091 | 0.018 | 3.38E-07 | 0.036 | 0.042 | 0.40 | |
| *Holdemania* | rs6960290 | C | T | 0.091 | 0.021 | 8.61E-06 | 0.008 | 0.040 | 0.85 | |
| *Holdemania* | rs73139538 | G | A | -0.149 | 0.033 | 5.69E-06 | -0.018 | 0.069 | 0.79 | |
| *Holdemania* | rs77293403 | A | G | 0.165 | 0.034 | 1.47E-06 | 0.005 | 0.064 | 0.94 | |
| *Holdemania* | rs80149660 | C | T | -0.233 | 0.052 | 7.20E-06 | 0.175 | 0.062 | 0.00 | |
| *Holdemania* | rs9500080 | C | T | 0.093 | 0.018 | 2.21E-07 | -0.006 | 0.037 | 0.87 | |
| *Holdemania* | rs9529719 | T | C | 0.074 | 0.016 | 3.97E-06 | 0.003 | 0.032 | 0.93 | |
| *Holdemania* | rs967319 | T | C | 0.079 | 0.018 | 8.12E-06 | -0.062 | 0.034 | 0.06 | |
| *Howardella* | rs12336926 | T | G | 0.114 | 0.026 | 8.65E-06 | 0.038 | 0.032 | 0.24 | |
| *Howardella* | rs12452946 | A | G | -0.106 | 0.023 | 3.79E-06 | -0.004 | 0.029 | 0.88 | |
| *Howardella* | rs1484873 | A | G | -0.228 | 0.046 | 8.79E-07 | 0.008 | 0.063 | 0.90 | |
| *Howardella* | rs17167098 | G | A | -0.169 | 0.035 | 1.50E-06 | -0.025 | 0.043 | 0.56 | |
| *Howardella* | rs2154047 | C | A | -0.193 | 0.042 | 4.57E-06 | 0.040 | 0.054 | 0.46 | |
| *Howardella* | rs36081916 | T | C | -0.181 | 0.040 | 6.89E-06 | -0.080 | 0.055 | 0.14 | |
| *Howardella* | rs609430 | T | G | -0.112 | 0.024 | 2.85E-06 | -0.078 | 0.031 | 0.01 | |
| *Howardella* | rs61771805 | A | T | -0.137 | 0.030 | 4.06E-06 | 0.045 | 0.036 | 0.20 | |
| *Howardella* | rs672217 | G | A | 0.164 | 0.035 | 2.73E-06 | -0.034 | 0.047 | 0.47 | |
| *Howardella* | rs6814441 | A | G | -0.104 | 0.023 | 8.53E-06 | -0.040 | 0.029 | 0.17 | |
| *Howardella* | rs901099 | T | G | -0.127 | 0.025 | 4.01E-07 | -0.040 | 0.033 | 0.23 | |
| *Hungatella* | rs12095986 | A | G | 0.183 | 0.041 | 9.98E-06 | -0.114 | 0.058 | 0.05 | |
| *Hungatella* | rs13249325 | T | G | -0.100 | 0.023 | 9.51E-06 | 0.010 | 0.030 | 0.73 | |
| *Hungatella* | rs17092615 | G | A | 0.152 | 0.034 | 6.61E-06 | 0.030 | 0.046 | 0.51 | |
| *Hungatella* | rs34471047 | A | G | -0.163 | 0.033 | 1.15E-06 | 0.095 | 0.044 | 0.03 | |
| *Hungatella* | rs62338229 | C | A | -0.128 | 0.029 | 9.39E-06 | -0.024 | 0.038 | 0.54 | |
| *Hungatella* | rs72759041 | G | T | -0.126 | 0.028 | 8.00E-06 | 0.025 | 0.040 | 0.54 | |
| *Intestinibacter* | rs11109097 | C | T | 0.062 | 0.014 | 6.60E-06 | -0.011 | 0.032 | 0.73 | |
| *Intestinibacter* | rs118030283 | G | A | -0.152 | 0.032 | 2.88E-06 | -0.068 | 0.072 | 0.35 | |
| *Intestinibacter* | rs16938435 | T | C | -0.112 | 0.024 | 1.89E-06 | 0.029 | 0.054 | 0.59 | |
| *Intestinibacter* | rs2098844 | C | T | -0.058 | 0.013 | 7.47E-06 | 0.063 | 0.030 | 0.03 | |
| *Intestinibacter* | rs2675411 | A | G | -0.065 | 0.015 | 9.29E-06 | -0.030 | 0.034 | 0.38 | |
| *Intestinibacter* | rs2702387 | A | G | 0.061 | 0.013 | 4.12E-06 | 0.026 | 0.031 | 0.40 | |
| *Intestinibacter* | rs307400 | A | T | -0.090 | 0.020 | 7.87E-06 | 0.026 | 0.051 | 0.61 | |
| *Intestinibacter* | rs4327025 | G | A | -0.081 | 0.015 | 1.53E-07 | -0.081 | 0.035 | 0.02 | |
| *Intestinibacter* | rs447950 | A | G | 0.063 | 0.014 | 4.26E-06 | -0.009 | 0.031 | 0.78 | |
| *Intestinibacter* | rs478972 | T | C | -0.143 | 0.030 | 1.57E-06 | 0.088 | 0.067 | 0.19 | |
| *Intestinibacter* | rs6062862 | A | G | 0.092 | 0.020 | 6.26E-06 | -0.027 | 0.046 | 0.55 | |
| *Intestinibacter* | rs6786654 | T | G | 0.067 | 0.015 | 9.74E-06 | -0.028 | 0.035 | 0.41 | |
| *Intestinibacter* | rs6875660 | C | T | 0.089 | 0.019 | 4.39E-06 | 0.072 | 0.049 | 0.14 | |
| *Intestinibacter* | rs893394 | G | A | 0.058 | 0.013 | 8.12E-06 | 0.004 | 0.030 | 0.91 | |
| *Intestinibacter* | rs9348442 | C | T | 0.099 | 0.022 | 7.80E-06 | -0.026 | 0.055 | 0.64 | |
| *Lachnoclostridium* | rs1031599 | G | T | -0.079 | 0.018 | 7.59E-06 | -0.009 | 0.055 | 0.87 | |
| *Lachnoclostridium* | rs117518699 | T | C | 0.114 | 0.025 | 7.04E-06 | -0.042 | 0.060 | 0.48 | |
| *Lachnoclostridium* | rs12566975 | T | C | -0.047 | 0.011 | 9.65E-06 | 0.023 | 0.029 | 0.44 | |
| *Lachnoclostridium* | rs1528479 | G | A | -0.050 | 0.011 | 8.67E-06 | 0.041 | 0.031 | 0.19 | |
| *Lachnoclostridium* | rs1997204 | T | C | -0.108 | 0.024 | 7.99E-06 | -0.020 | 0.066 | 0.76 | |
| *Lachnoclostridium* | rs3821998 | C | A | -0.086 | 0.019 | 7.18E-06 | -0.073 | 0.053 | 0.17 | |
| *Lachnoclostridium* | rs4738679 | G | A | -0.052 | 0.011 | 5.06E-06 | 0.025 | 0.031 | 0.42 | |
| *Lachnoclostridium* | rs6112314 | A | C | -0.056 | 0.011 | 2.07E-07 | -0.049 | 0.030 | 0.10 | |
| *Lachnoclostridium* | rs615997 | T | C | 0.051 | 0.011 | 1.54E-06 | -0.008 | 0.029 | 0.80 | |
| *Lachnoclostridium* | rs61915992 | A | T | 0.080 | 0.017 | 3.04E-06 | 0.018 | 0.046 | 0.69 | |
| *Lachnoclostridium* | rs62028349 | G | C | 0.047 | 0.011 | 9.20E-06 | 0.009 | 0.029 | 0.77 | |
| *Lachnoclostridium* | rs62285313 | A | G | 0.086 | 0.018 | 1.94E-06 | -0.035 | 0.048 | 0.46 | |
| *Lachnoclostridium* | rs78068103 | A | G | 0.089 | 0.019 | 5.06E-06 | -0.004 | 0.061 | 0.95 | |
| *Lachnoclostridium* | rs789029 | C | T | -0.064 | 0.014 | 3.35E-06 | -0.009 | 0.039 | 0.83 | |
| *Lachnospira* | rs13157098 | A | G | -0.077 | 0.016 | 7.60E-07 | -0.159 | 0.042 | 0.00 | |
| *Lachnospira* | rs2326833 | C | G | -0.078 | 0.017 | 4.48E-06 | -0.024 | 0.047 | 0.61 | |
| *Lachnospira* | rs2520509 | A | G | 0.052 | 0.012 | 7.35E-06 | -0.025 | 0.031 | 0.42 | |
| *Lachnospira* | rs35418758 | A | C | 0.086 | 0.019 | 5.53E-06 | -0.113 | 0.053 | 0.03 | |
| *Lachnospira* | rs4686798 | T | C | 0.053 | 0.011 | 2.93E-06 | 0.064 | 0.030 | 0.03 | |
| *Lachnospira* | rs4923324 | G | A | -0.062 | 0.013 | 3.69E-06 | 0.022 | 0.033 | 0.52 | |
| *Lachnospira* | rs56791201 | T | C | 0.052 | 0.011 | 2.85E-06 | 0.030 | 0.029 | 0.30 | |
| *Lachnospiraceae (FCS020 group)* | rs10030408 | A | G | 0.055 | 0.012 | 7.48E-06 | -0.037 | 0.030 | 0.21 | |
| *Lachnospiraceae (FCS020 group)* | rs10093861 | G | A | -0.057 | 0.012 | 2.66E-06 | 0.008 | 0.029 | 0.77 | |
| *Lachnospiraceae (FCS020 group)* | rs113859143 | G | C | -0.109 | 0.024 | 6.82E-06 | 0.006 | 0.058 | 0.92 | |
| *Lachnospiraceae (FCS020 group)* | rs12078956 | C | G | 0.106 | 0.022 | 1.94E-06 | 0.075 | 0.064 | 0.24 | |
| *Lachnospiraceae (FCS020 group)* | rs1254846 | G | A | 0.106 | 0.023 | 5.18E-06 | -0.010 | 0.057 | 0.87 | |
| *Lachnospiraceae (FCS020 group)* | rs1363769 | T | C | -0.201 | 0.045 | 8.02E-06 | 0.004 | 0.066 | 0.95 | |
| *Lachnospiraceae (FCS020 group)* | rs2322265 | C | T | -0.067 | 0.014 | 2.52E-06 | -0.007 | 0.035 | 0.84 | |
| *Lachnospiraceae (FCS020 group)* | rs2862811 | T | C | 0.056 | 0.012 | 3.47E-06 | -0.005 | 0.029 | 0.86 | |
| *Lachnospiraceae (FCS020 group)* | rs35035870 | T | C | -0.191 | 0.041 | 4.23E-06 | -0.033 | 0.073 | 0.65 | |
| *Lachnospiraceae (FCS020 group)* | rs369444 | C | G | 0.125 | 0.026 | 1.27E-06 | -0.046 | 0.063 | 0.47 | |
| *Lachnospiraceae (FCS020 group)* | rs3999074 | G | T | -0.055 | 0.012 | 6.22E-06 | 0.003 | 0.030 | 0.91 | |
| *Lachnospiraceae (FCS020 group)* | rs4358521 | C | G | -0.062 | 0.014 | 9.95E-06 | 0.051 | 0.033 | 0.12 | |
| *Lachnospiraceae (FCS020 group)* | rs4452603 | T | G | 0.060 | 0.014 | 8.84E-06 | 0.029 | 0.035 | 0.42 | |
| *Lachnospiraceae (FCS020 group)* | rs57049931 | G | A | -0.101 | 0.022 | 6.44E-06 | 0.017 | 0.062 | 0.78 | |
| *Lachnospiraceae (FCS020 group)* | rs7249113 | G | A | 0.068 | 0.013 | 3.58E-07 | 0.089 | 0.033 | 0.01 | |
| *Lachnospiraceae (FCS020 group)* | rs72793667 | A | G | -0.117 | 0.025 | 2.14E-06 | -0.015 | 0.057 | 0.80 | |
| *Lachnospiraceae (FCS020 group)* | rs9788306 | C | T | -0.063 | 0.013 | 1.56E-06 | 0.101 | 0.032 | 0.00 | |
| *Lachnospiraceae (FCS020 group)* | rs9919338 | G | C | -0.055 | 0.012 | 4.49E-06 | -0.017 | 0.029 | 0.56 | |
| *Lachnospiraceae (NC2004 group)* | rs10774762 | A | G | -0.085 | 0.019 | 7.72E-06 | -0.021 | 0.031 | 0.49 | |
| *Lachnospiraceae (NC2004 group)* | rs117467633 | T | C | -0.170 | 0.038 | 9.49E-06 | -0.028 | 0.063 | 0.65 | |
| *Lachnospiraceae (NC2004 group)* | rs12127733 | G | A | 0.115 | 0.025 | 2.84E-06 | 0.099 | 0.039 | 0.01 | |
| *Lachnospiraceae (NC2004 group)* | rs12208226 | C | A | -0.155 | 0.034 | 5.46E-06 | -0.042 | 0.053 | 0.42 | |
| *Lachnospiraceae (NC2004 group)* | rs12863463 | G | A | -0.156 | 0.035 | 5.97E-06 | 0.048 | 0.055 | 0.39 | |
| *Lachnospiraceae (NC2004 group)* | rs1331592 | C | G | 0.095 | 0.021 | 5.28E-06 | 0.065 | 0.035 | 0.06 | |
| *Lachnospiraceae (NC2004 group)* | rs1567127 | G | A | 0.103 | 0.023 | 5.72E-06 | 0.014 | 0.037 | 0.70 | |
| *Lachnospiraceae (NC2004 group)* | rs1928659 | T | C | 0.103 | 0.023 | 5.97E-06 | -0.020 | 0.033 | 0.55 | |
| *Lachnospiraceae (NC2004 group)* | rs3756315 | A | G | -0.088 | 0.019 | 2.74E-06 | -0.042 | 0.032 | 0.19 | |
| *Lachnospiraceae (NC2004 group)* | rs6116753 | G | A | 0.099 | 0.021 | 1.97E-06 | -0.038 | 0.033 | 0.25 | |
| *Lachnospiraceae (ND3007 group)* | rs13110238 | C | G | -0.064 | 0.014 | 7.31E-06 | -0.016 | 0.038 | 0.66 | |
| *Lachnospiraceae (ND3007 group)* | rs2861203 | G | A | 0.057 | 0.013 | 6.91E-06 | -0.041 | 0.038 | 0.29 | |
| *Lachnospiraceae (ND3007 group)* | rs4897506 | C | T | -0.072 | 0.016 | 9.34E-06 | -0.014 | 0.040 | 0.72 | |
| *Lachnospiraceae (ND3007 group)* | rs6477796 | G | A | 0.052 | 0.012 | 9.32E-06 | -0.027 | 0.030 | 0.36 | |
| *Lachnospiraceae (ND3007 group)* | rs9932954 | A | G | -0.056 | 0.012 | 1.27E-06 | 0.031 | 0.029 | 0.28 | |
| *Lachnospiraceae (NK4A136 group)* | rs10952110 | G | T | 0.049 | 0.011 | 8.61E-06 | -0.053 | 0.030 | 0.08 | |
| *Lachnospiraceae (NK4A136 group)* | rs11263806 | A | G | -0.052 | 0.012 | 7.02E-06 | -0.024 | 0.033 | 0.47 | |
| *Lachnospiraceae (NK4A136 group)* | rs12362320 | G | C | 0.057 | 0.012 | 6.91E-07 | 0.004 | 0.031 | 0.90 | |
| *Lachnospiraceae (NK4A136 group)* | rs12611395 | A | G | -0.090 | 0.020 | 6.17E-06 | 0.014 | 0.052 | 0.78 | |
| *Lachnospiraceae (NK4A136 group)* | rs13384748 | A | C | -0.078 | 0.018 | 8.23E-06 | -0.001 | 0.051 | 0.99 | |
| *Lachnospiraceae (NK4A136 group)* | rs13401508 | G | A | 0.181 | 0.040 | 6.13E-06 | -0.226 | 0.141 | 0.11 | |
| *Lachnospiraceae (NK4A136 group)* | rs160061 | A | G | 0.051 | 0.011 | 2.00E-06 | 0.024 | 0.029 | 0.42 | |
| *Lachnospiraceae (NK4A136 group)* | rs28540839 | A | C | 0.051 | 0.011 | 4.31E-06 | 0.056 | 0.030 | 0.06 | |
| *Lachnospiraceae (NK4A136 group)* | rs2880566 | T | C | 0.060 | 0.013 | 8.53E-06 | -0.040 | 0.036 | 0.27 | |
| *Lachnospiraceae (NK4A136 group)* | rs4955932 | T | C | -0.049 | 0.011 | 6.78E-06 | -0.012 | 0.031 | 0.70 | |
| *Lachnospiraceae (NK4A136 group)* | rs59805249 | T | C | 0.094 | 0.021 | 6.76E-06 | 0.043 | 0.062 | 0.48 | |
| *Lachnospiraceae (NK4A136 group)* | rs68104925 | T | C | -0.055 | 0.012 | 1.95E-06 | -0.035 | 0.030 | 0.25 | |
| *Lachnospiraceae (NK4A136 group)* | rs7073658 | T | G | -0.050 | 0.011 | 5.25E-06 | -0.029 | 0.030 | 0.33 | |
| *Lachnospiraceae (NK4A136 group)* | rs7616165 | G | T | -0.231 | 0.048 | 1.86E-06 | 0.004 | 0.069 | 0.96 | |
| *Lachnospiraceae (NK4A136 group)* | rs76193507 | A | G | -0.230 | 0.050 | 4.29E-06 | 0.050 | 0.068 | 0.47 | |
| *Lachnospiraceae (NK4A136 group)* | rs7832116 | A | G | -0.071 | 0.015 | 2.46E-06 | 0.027 | 0.043 | 0.53 | |
| *Lachnospiraceae (NK4A136 group)* | rs954878 | A | G | -0.052 | 0.011 | 1.81E-06 | -0.022 | 0.030 | 0.46 | |
| *Lachnospiraceae (UCG001)* | rs10815577 | C | G | -0.068 | 0.014 | 1.95E-06 | -0.044 | 0.030 | 0.15 | |
| *Lachnospiraceae (UCG001)* | rs12131224 | C | T | 0.117 | 0.026 | 6.20E-06 | 0.144 | 0.055 | 0.01 | |
| *Lachnospiraceae (UCG001)* | rs2050911 | G | A | 0.075 | 0.015 | 1.05E-06 | 0.001 | 0.032 | 0.97 | |
| *Lachnospiraceae (UCG001)* | rs2371284 | T | C | -0.076 | 0.017 | 7.52E-06 | 0.032 | 0.037 | 0.39 | |
| *Lachnospiraceae (UCG001)* | rs437876 | T | C | 0.078 | 0.014 | 5.96E-08 | 0.036 | 0.031 | 0.25 | |
| *Lachnospiraceae (UCG001)* | rs4981345 | T | C | -0.068 | 0.015 | 5.32E-06 | -0.043 | 0.035 | 0.21 | |
| *Lachnospiraceae (UCG001)* | rs573933 | T | C | -0.108 | 0.023 | 3.42E-06 | -0.015 | 0.052 | 0.77 | |
| *Lachnospiraceae (UCG001)* | rs62496417 | T | G | -0.075 | 0.017 | 6.25E-06 | 0.043 | 0.034 | 0.20 | |
| *Lachnospiraceae (UCG001)* | rs626012 | C | T | -0.127 | 0.028 | 6.47E-06 | 0.017 | 0.064 | 0.79 | |
| *Lachnospiraceae (UCG001)* | rs7213933 | T | A | -0.082 | 0.018 | 8.95E-06 | 0.006 | 0.043 | 0.89 | |
| *Lachnospiraceae (UCG001)* | rs7341608 | T | C | -0.078 | 0.018 | 9.99E-06 | -0.008 | 0.038 | 0.84 | |
| *Lachnospiraceae (UCG001)* | rs78848836 | A | G | -0.119 | 0.026 | 4.74E-06 | -0.058 | 0.055 | 0.29 | |
| *Lachnospiraceae (UCG001)* | rs79476906 | T | A | -0.087 | 0.020 | 9.85E-06 | 0.010 | 0.040 | 0.80 | |
| *Lachnospiraceae (UCG001)* | rs8052586 | T | C | 0.176 | 0.040 | 8.42E-06 | 0.077 | 0.073 | 0.29 | |
| *Lachnospiraceae (UCG001)* | rs8104225 | A | G | 0.089 | 0.020 | 6.39E-06 | 0.027 | 0.049 | 0.57 | |
| *Lachnospiraceae (UCG001)* | rs9403580 | C | T | 0.108 | 0.023 | 2.71E-06 | 0.020 | 0.048 | 0.67 | |
| *Lachnospiraceae (UCG001)* | rs985416 | C | T | 0.097 | 0.018 | 9.46E-08 | 0.025 | 0.037 | 0.50 | |
| *Lachnospiraceae (UCG004)* | rs11128180 | A | G | 0.065 | 0.014 | 3.72E-06 | -0.069 | 0.035 | 0.05 | |
| *Lachnospiraceae (UCG004)* | rs117276182 | G | A | 0.114 | 0.025 | 7.71E-06 | -0.008 | 0.058 | 0.90 | |
| *Lachnospiraceae (UCG004)* | rs12673420 | G | A | 0.055 | 0.012 | 2.83E-06 | 0.032 | 0.029 | 0.28 | |
| *Lachnospiraceae (UCG004)* | rs12747809 | G | A | -0.062 | 0.013 | 7.47E-07 | 0.061 | 0.031 | 0.05 | |
| *Lachnospiraceae (UCG004)* | rs12894272 | A | G | 0.058 | 0.013 | 3.66E-06 | 0.048 | 0.031 | 0.13 | |
| *Lachnospiraceae (UCG004)* | rs233486 | A | G | -0.080 | 0.018 | 6.86E-06 | -0.031 | 0.046 | 0.49 | |
| *Lachnospiraceae (UCG004)* | rs2444793 | C | T | -0.054 | 0.012 | 4.44E-06 | -0.028 | 0.029 | 0.33 | |
| *Lachnospiraceae (UCG004)* | rs2706242 | G | C | -0.090 | 0.020 | 6.38E-06 | 0.075 | 0.050 | 0.13 | |
| *Lachnospiraceae (UCG004)* | rs2726805 | A | G | 0.055 | 0.012 | 5.68E-06 | -0.041 | 0.032 | 0.20 | |
| *Lachnospiraceae (UCG004)* | rs2882478 | G | A | -0.058 | 0.012 | 1.08E-06 | -0.060 | 0.029 | 0.04 | |
| *Lachnospiraceae (UCG004)* | rs35182105 | A | G | -0.110 | 0.024 | 5.89E-06 | -0.011 | 0.059 | 0.85 | |
| *Lachnospiraceae (UCG004)* | rs6656451 | C | T | -0.054 | 0.012 | 5.36E-06 | 0.036 | 0.029 | 0.22 | |
| *Lachnospiraceae (UCG004)* | rs7629954 | A | G | 0.108 | 0.024 | 5.32E-06 | -0.143 | 0.072 | 0.05 | |
| *Lachnospiraceae (UCG004)* | rs7833187 | C | T | -0.083 | 0.018 | 6.82E-06 | -0.009 | 0.047 | 0.84 | |
| *Lachnospiraceae (UCG008)* | rs10741777 | T | C | -0.097 | 0.019 | 5.75E-07 | -0.018 | 0.035 | 0.61 | |
| *Lachnospiraceae (UCG008)* | rs10793103 | C | T | 0.097 | 0.018 | 7.67E-08 | -0.012 | 0.031 | 0.71 | |
| *Lachnospiraceae (UCG008)* | rs10801803 | G | A | -0.117 | 0.024 | 1.48E-06 | -0.033 | 0.043 | 0.45 | |
| *Lachnospiraceae (UCG008)* | rs12594517 | G | A | -0.102 | 0.023 | 8.26E-06 | 0.055 | 0.042 | 0.20 | |
| *Lachnospiraceae (UCG008)* | rs13024781 | T | C | -0.080 | 0.017 | 2.24E-06 | -0.005 | 0.029 | 0.87 | |
| *Lachnospiraceae (UCG008)* | rs57091572 | A | G | -0.110 | 0.024 | 2.82E-06 | -0.027 | 0.041 | 0.51 | |
| *Lachnospiraceae (UCG008)* | rs57254474 | G | A | 0.089 | 0.020 | 8.44E-06 | 0.024 | 0.034 | 0.48 | |
| *Lachnospiraceae (UCG008)* | rs61944774 | A | G | 0.180 | 0.039 | 4.95E-06 | -0.001 | 0.065 | 0.98 | |
| *Lachnospiraceae (UCG008)* | rs62277846 | C | T | 0.102 | 0.021 | 1.45E-06 | -0.108 | 0.039 | 0.01 | |
| *Lachnospiraceae (UCG008)* | rs67078837 | T | C | -0.085 | 0.017 | 7.22E-07 | 0.031 | 0.029 | 0.30 | |
| *Lachnospiraceae (UCG008)* | rs75326324 | G | A | 0.158 | 0.036 | 9.26E-06 | 0.046 | 0.063 | 0.47 | |
| *Lachnospiraceae (UCG008)* | rs75356640 | G | A | 0.137 | 0.030 | 6.67E-06 | -0.045 | 0.055 | 0.41 | |
| *Lachnospiraceae (UCG008)* | rs955844 | A | C | 0.112 | 0.023 | 9.24E-07 | -0.046 | 0.043 | 0.29 | |
| *Lachnospiraceae (UCG008)* | rs9873555 | G | C | -0.121 | 0.023 | 2.11E-07 | -0.017 | 0.039 | 0.66 | |
| *Lachnospiraceae (UCG010)* | rs11192447 | A | G | 0.127 | 0.024 | 1.99E-07 | 0.013 | 0.067 | 0.84 | |
| *Lachnospiraceae (UCG010)* | rs12346653 | C | T | 0.066 | 0.014 | 2.45E-06 | 0.000 | 0.034 | 0.99 | |
| *Lachnospiraceae (UCG010)* | rs17730011 | G | A | -0.070 | 0.016 | 7.75E-06 | 0.024 | 0.038 | 0.53 | |
| *Lachnospiraceae (UCG010)* | rs336138 | G | T | 0.078 | 0.017 | 5.74E-06 | 0.093 | 0.039 | 0.02 | |
| *Lachnospiraceae (UCG010)* | rs4576377 | A | C | -0.057 | 0.013 | 6.72E-06 | -0.017 | 0.030 | 0.57 | |
| *Lachnospiraceae (UCG010)* | rs72761829 | A | T | 0.112 | 0.024 | 2.82E-06 | -0.076 | 0.056 | 0.17 | |
| *Lachnospiraceae (UCG010)* | rs72894957 | G | A | 0.222 | 0.049 | 4.89E-06 | 0.110 | 0.071 | 0.12 | |
| *Lachnospiraceae (UCG010)* | rs74315802 | G | T | 0.087 | 0.018 | 2.28E-06 | -0.048 | 0.047 | 0.30 | |
| *Lachnospiraceae (UCG010)* | rs7949326 | G | C | -0.110 | 0.025 | 8.56E-06 | -0.020 | 0.063 | 0.76 | |
| *Lachnospiraceae (UCG010)* | rs9981767 | A | C | 0.066 | 0.013 | 6.95E-07 | -0.026 | 0.032 | 0.40 | |
| *Lactobacillus* | rs11674854 | C | T | -0.085 | 0.018 | 1.36E-06 | 0.007 | 0.030 | 0.81 | |
| *Lactobacillus* | rs12693845 | C | T | -0.081 | 0.018 | 5.64E-06 | -0.021 | 0.037 | 0.56 | |
| *Lactobacillus* | rs1530559 | G | A | 0.080 | 0.018 | 6.43E-06 | 0.041 | 0.032 | 0.20 | |
| *Lactobacillus* | rs16861661 | G | A | -0.183 | 0.038 | 1.58E-06 | 0.003 | 0.072 | 0.96 | |
| *Lactobacillus* | rs328312 | T | A | 0.082 | 0.017 | 1.51E-06 | -0.018 | 0.029 | 0.54 | |
| *Lactobacillus* | rs6092149 | A | T | -0.080 | 0.017 | 2.97E-06 | 0.024 | 0.029 | 0.41 | |
| *Lactobacillus* | rs62314653 | C | A | 0.188 | 0.039 | 1.97E-06 | 0.052 | 0.070 | 0.46 | |
| *Lactobacillus* | rs7399658 | G | A | -0.107 | 0.022 | 1.38E-06 | 0.007 | 0.036 | 0.85 | |
| *Lactobacillus* | rs75127669 | C | A | 0.140 | 0.031 | 6.70E-06 | 0.062 | 0.056 | 0.26 | |
| *Lactobacillus* | rs768253 | T | G | -0.079 | 0.017 | 4.03E-06 | -0.043 | 0.030 | 0.15 | |
| *Lactobacillus* | rs77478751 | A | G | -0.220 | 0.048 | 3.80E-06 | -0.006 | 0.083 | 0.94 | |
| *Lactobacillus* | rs921925 | A | C | 0.099 | 0.020 | 1.25E-06 | -0.028 | 0.036 | 0.44 | |
| *Lactococcus* | rs10417872 | T | G | 0.118 | 0.025 | 1.40E-06 | -0.003 | 0.032 | 0.93 | |
| *Lactococcus* | rs123059 | T | C | -0.137 | 0.027 | 6.46E-07 | -0.050 | 0.039 | 0.20 | |
| *Lactococcus* | rs12621813 | G | A | 0.108 | 0.024 | 6.24E-06 | 0.040 | 0.032 | 0.21 | |
| *Lactococcus* | rs17168302 | G | A | 0.192 | 0.042 | 6.28E-06 | 0.025 | 0.061 | 0.68 | |
| *Lactococcus* | rs2293361 | C | T | -0.199 | 0.043 | 3.79E-06 | 0.038 | 0.060 | 0.53 | |
| *Lactococcus* | rs34757988 | G | C | 0.122 | 0.023 | 9.25E-08 | -0.014 | 0.030 | 0.65 | |
| *Lactococcus* | rs4766997 | C | T | 0.115 | 0.024 | 1.53E-06 | 0.068 | 0.032 | 0.03 | |
| *Lactococcus* | rs55910161 | C | T | 0.146 | 0.031 | 1.90E-06 | -0.066 | 0.039 | 0.09 | |
| *Lactococcus* | rs6674304 | C | T | 0.201 | 0.044 | 5.60E-06 | 0.061 | 0.060 | 0.31 | |
| *Lactococcus* | rs757872 | G | C | 0.141 | 0.028 | 3.31E-07 | 0.000 | 0.035 | 0.99 | |
| *Lactococcus* | rs7992246 | T | C | 0.104 | 0.023 | 6.29E-06 | -0.012 | 0.032 | 0.70 | |
| *Marvinbryantia* | rs11645029 | G | C | -0.061 | 0.013 | 4.07E-06 | 0.009 | 0.030 | 0.76 | |
| *Marvinbryantia* | rs1187983 | C | T | -0.094 | 0.019 | 1.28E-06 | -0.026 | 0.048 | 0.59 | |
| *Marvinbryantia* | rs12963345 | G | C | -0.060 | 0.013 | 6.44E-06 | -0.011 | 0.036 | 0.77 | |
| *Marvinbryantia* | rs146541147 | G | A | 0.119 | 0.027 | 9.53E-06 | -0.056 | 0.062 | 0.37 | |
| *Marvinbryantia* | rs17042065 | G | A | -0.104 | 0.024 | 9.60E-06 | 0.031 | 0.056 | 0.58 | |
| *Marvinbryantia* | rs2724813 | A | G | -0.084 | 0.017 | 5.22E-07 | -0.049 | 0.038 | 0.20 | |
| *Marvinbryantia* | rs2842896 | C | T | -0.065 | 0.013 | 7.36E-07 | 0.051 | 0.030 | 0.09 | |
| *Marvinbryantia* | rs2863363 | A | G | 0.063 | 0.014 | 3.21E-06 | 0.005 | 0.030 | 0.86 | |
| *Marvinbryantia* | rs3125832 | A | C | 0.068 | 0.015 | 6.03E-06 | -0.034 | 0.034 | 0.31 | |
| *Marvinbryantia* | rs61884471 | G | A | 0.124 | 0.025 | 5.49E-07 | 0.035 | 0.057 | 0.53 | |
| *Marvinbryantia* | rs72948274 | A | C | -0.126 | 0.027 | 3.45E-06 | 0.005 | 0.069 | 0.94 | |
| *Methanobrevibacter* | rs10202904 | T | G | -0.113 | 0.024 | 2.38E-06 | 0.037 | 0.029 | 0.21 | |
| *Methanobrevibacter* | rs11018665 | A | T | 0.113 | 0.025 | 8.86E-06 | 0.074 | 0.031 | 0.02 | |
| *Methanobrevibacter* | rs113023771 | A | G | -0.221 | 0.049 | 5.59E-06 | 0.025 | 0.066 | 0.71 | |
| *Methanobrevibacter* | rs1334944 | T | C | 0.115 | 0.026 | 6.52E-06 | 0.001 | 0.031 | 0.97 | |
| *Methanobrevibacter* | rs4779844 | G | C | 0.110 | 0.025 | 9.81E-06 | -0.005 | 0.031 | 0.87 | |
| *Methanobrevibacter* | rs6004341 | C | T | 0.225 | 0.051 | 9.49E-06 | 0.022 | 0.060 | 0.71 | |
| *Methanobrevibacter* | rs6776814 | T | C | -0.189 | 0.042 | 6.80E-06 | -0.006 | 0.050 | 0.91 | |
| *Methanobrevibacter* | rs73457410 | A | G | 0.218 | 0.044 | 9.32E-07 | -0.049 | 0.055 | 0.38 | |
| *Methanobrevibacter* | rs75208022 | C | T | -0.222 | 0.049 | 6.02E-06 | -0.025 | 0.057 | 0.66 | |
| *Methanobrevibacter* | rs894996 | C | A | 0.214 | 0.046 | 2.64E-06 | 0.009 | 0.058 | 0.87 | |
| *Odoribacter* | rs10093869 | A | G | -0.058 | 0.013 | 4.07E-06 | -0.036 | 0.031 | 0.26 | |
| *Odoribacter* | rs10423795 | C | T | 0.055 | 0.012 | 5.49E-06 | 0.007 | 0.032 | 0.82 | |
| *Odoribacter* | rs11959849 | T | C | -0.145 | 0.032 | 7.75E-06 | 0.137 | 0.066 | 0.04 | |
| *Odoribacter* | rs16918425 | A | T | 0.100 | 0.022 | 7.91E-06 | -0.061 | 0.059 | 0.30 | |
| *Odoribacter* | rs28417404 | A | G | -0.073 | 0.016 | 6.65E-06 | 0.024 | 0.040 | 0.54 | |
| *Odoribacter* | rs34627176 | A | G | -0.067 | 0.015 | 8.42E-06 | 0.002 | 0.036 | 0.95 | |
| *Odoribacter* | rs34677296 | T | A | 0.068 | 0.015 | 9.04E-06 | -0.040 | 0.038 | 0.30 | |
| *Odoribacter* | rs4793970 | A | G | -0.058 | 0.013 | 8.11E-06 | -0.091 | 0.033 | 0.01 | |
| *Odoribacter* | rs503751 | C | G | 0.062 | 0.012 | 1.99E-07 | -0.005 | 0.029 | 0.86 | |
| *Odoribacter* | rs6856150 | G | A | 0.088 | 0.019 | 5.56E-06 | -0.055 | 0.053 | 0.31 | |
| *Odoribacter* | rs74553962 | T | G | 0.121 | 0.026 | 4.26E-06 | 0.081 | 0.064 | 0.20 | |
| *Odoribacter* | rs74810361 | A | G | 0.220 | 0.049 | 6.24E-06 | 0.027 | 0.075 | 0.72 | |
| *Odoribacter* | rs77779484 | G | A | -0.133 | 0.027 | 6.65E-07 | 0.155 | 0.066 | 0.02 | |
| *Olsenella* | rs1035588 | A | G | -0.108 | 0.024 | 4.97E-06 | 0.007 | 0.030 | 0.82 | |
| *Olsenella* | rs11603650 | A | T | -0.103 | 0.023 | 9.82E-06 | -0.046 | 0.030 | 0.13 | |
| *Olsenella* | rs17148768 | G | A | 0.140 | 0.030 | 2.03E-06 | 0.025 | 0.039 | 0.52 | |
| *Olsenella* | rs2759329 | G | A | -0.111 | 0.024 | 2.80E-06 | -0.068 | 0.030 | 0.02 | |
| *Olsenella* | rs35225860 | A | G | -0.224 | 0.048 | 3.56E-06 | -0.021 | 0.064 | 0.74 | |
| *Olsenella* | rs6046522 | C | T | 0.123 | 0.027 | 5.25E-06 | 0.012 | 0.034 | 0.73 | |
| *Olsenella* | rs61090148 | A | G | -0.105 | 0.023 | 5.92E-06 | -0.061 | 0.031 | 0.05 | |
| *Olsenella* | rs62112538 | C | T | -0.199 | 0.041 | 9.60E-07 | 0.067 | 0.056 | 0.23 | |
| *Olsenella* | rs72691585 | C | A | -0.249 | 0.052 | 1.73E-06 | -0.051 | 0.065 | 0.43 | |
| *Olsenella* | rs7540303 | C | T | 0.108 | 0.024 | 4.86E-06 | 0.054 | 0.030 | 0.07 | |
| *Olsenella* | rs775859 | T | C | 0.190 | 0.042 | 6.73E-06 | 0.070 | 0.056 | 0.21 | |
| *Olsenella* | rs8066522 | G | A | -0.107 | 0.024 | 9.35E-06 | -0.017 | 0.034 | 0.63 | |
| *Olsenella* | rs9460691 | C | A | 0.120 | 0.027 | 7.98E-06 | -0.028 | 0.037 | 0.45 | |
| *Oscillibacter* | rs11627628 | T | C | 0.144 | 0.029 | 7.04E-07 | -0.168 | 0.070 | 0.02 | |
| *Oscillibacter* | rs11990279 | T | C | -0.082 | 0.018 | 4.85E-06 | 0.012 | 0.034 | 0.71 | |
| *Oscillibacter* | rs12417956 | C | G | 0.079 | 0.017 | 6.42E-06 | -0.011 | 0.034 | 0.75 | |
| *Oscillibacter* | rs12649930 | T | G | 0.122 | 0.026 | 2.82E-06 | -0.009 | 0.053 | 0.86 | |
| *Oscillibacter* | rs133832 | A | C | -0.080 | 0.016 | 9.67E-07 | 0.011 | 0.031 | 0.72 | |
| *Oscillibacter* | rs137917150 | T | A | -0.175 | 0.039 | 6.62E-06 | -0.089 | 0.069 | 0.20 | |
| *Oscillibacter* | rs16866406 | A | G | 0.099 | 0.021 | 2.18E-06 | -0.068 | 0.042 | 0.10 | |
| *Oscillibacter* | rs16934185 | A | G | -0.130 | 0.028 | 4.19E-06 | -0.097 | 0.052 | 0.06 | |
| *Oscillibacter* | rs234108 | A | G | 0.075 | 0.015 | 9.07E-07 | 0.038 | 0.029 | 0.20 | |
| *Oscillibacter* | rs36095275 | C | T | -0.075 | 0.016 | 1.62E-06 | 0.050 | 0.030 | 0.10 | |
| *Oscillibacter* | rs4506202 | A | G | -0.071 | 0.015 | 2.99E-06 | -0.028 | 0.038 | 0.47 | |
| *Oscillibacter* | rs61883564 | A | G | -0.101 | 0.022 | 4.52E-06 | -0.017 | 0.044 | 0.70 | |
| *Oscillibacter* | rs62206502 | C | A | -0.068 | 0.015 | 6.72E-06 | 0.049 | 0.029 | 0.09 | |
| *Oscillibacter* | rs6901560 | C | G | 0.086 | 0.019 | 4.78E-06 | -0.051 | 0.036 | 0.16 | |
| *Oscillibacter* | rs75453768 | G | T | 0.122 | 0.027 | 5.47E-06 | -0.001 | 0.057 | 0.99 | |
| *Oscillibacter* | rs761240 | T | G | -0.177 | 0.039 | 5.54E-06 | -0.028 | 0.072 | 0.69 | |
| *Oscillibacter* | rs9393920 | A | G | -0.074 | 0.015 | 8.27E-07 | 0.058 | 0.029 | 0.05 | |
| *Oscillospira* | rs12206468 | G | A | -0.133 | 0.027 | 8.17E-07 | 0.051 | 0.054 | 0.35 | |
| *Oscillospira* | rs12586346 | A | G | 0.117 | 0.026 | 7.60E-06 | -0.020 | 0.064 | 0.76 | |
| *Oscillospira* | rs1954532 | T | C | -0.083 | 0.018 | 2.42E-06 | -0.001 | 0.039 | 0.98 | |
| *Oscillospira* | rs28889936 | A | C | 0.114 | 0.025 | 6.46E-06 | 0.027 | 0.056 | 0.63 | |
| *Oscillospira* | rs35516923 | A | G | -0.076 | 0.017 | 9.36E-06 | -0.030 | 0.034 | 0.37 | |
| *Oscillospira* | rs3758971 | T | C | 0.134 | 0.030 | 7.78E-06 | -0.122 | 0.067 | 0.07 | |
| *Oscillospira* | rs62422654 | C | T | 0.090 | 0.020 | 5.74E-06 | -0.023 | 0.043 | 0.60 | |
| *Oscillospira* | rs72866977 | A | C | -0.131 | 0.028 | 3.56E-06 | -0.030 | 0.058 | 0.61 | |
| *Oscillospira* | rs73038677 | T | A | -0.083 | 0.017 | 8.08E-07 | -0.025 | 0.037 | 0.49 | |
| *Oscillospira* | rs751183 | T | C | -0.077 | 0.017 | 6.92E-06 | 0.031 | 0.037 | 0.40 | |
| *Oscillospira* | rs75570604 | C | G | 0.137 | 0.031 | 8.11E-06 | -0.022 | 0.049 | 0.65 | |
| *Oscillospira* | rs8076323 | A | G | 0.072 | 0.016 | 4.88E-06 | -0.033 | 0.034 | 0.33 | |
| *Oxalobacter* | rs10464997 | G | A | 0.138 | 0.029 | 3.00E-06 | 0.009 | 0.044 | 0.85 | |
| *Oxalobacter* | rs11108500 | A | G | -0.199 | 0.043 | 3.17E-06 | -0.061 | 0.068 | 0.36 | |
| *Oxalobacter* | rs111966731 | T | C | 0.213 | 0.047 | 6.22E-06 | -0.007 | 0.061 | 0.90 | |
| *Oxalobacter* | rs12002250 | A | C | 0.217 | 0.047 | 3.22E-06 | 0.054 | 0.073 | 0.46 | |
| *Oxalobacter* | rs1569853 | T | C | -0.138 | 0.030 | 3.33E-06 | 0.106 | 0.040 | 0.01 | |
| *Oxalobacter* | rs36057338 | G | T | 0.208 | 0.042 | 8.15E-07 | 0.050 | 0.057 | 0.38 | |
| *Oxalobacter* | rs4428215 | G | A | 0.130 | 0.024 | 7.50E-08 | -0.055 | 0.035 | 0.12 | |
| *Oxalobacter* | rs6000536 | C | T | -0.131 | 0.025 | 2.45E-07 | -0.001 | 0.037 | 0.98 | |
| *Oxalobacter* | rs6071435 | T | A | -0.106 | 0.021 | 9.10E-07 | -0.013 | 0.030 | 0.68 | |
| *Oxalobacter* | rs6993398 | G | A | 0.127 | 0.028 | 5.06E-06 | -0.037 | 0.041 | 0.37 | |
| *Oxalobacter* | rs736744 | C | T | 0.118 | 0.021 | 2.41E-08 | 0.011 | 0.030 | 0.71 | |
| *Oxalobacter* | rs76681900 | G | T | -0.174 | 0.039 | 8.16E-06 | 0.104 | 0.056 | 0.06 | |
| *Oxalobacter* | rs9321445 | C | T | 0.104 | 0.023 | 7.44E-06 | -0.053 | 0.033 | 0.11 | |
| *Parabacteroides* | rs114567323 | T | C | 0.186 | 0.041 | 4.18E-06 | -0.028 | 0.086 | 0.75 | |
| *Parabacteroides* | rs115602804 | G | A | 0.103 | 0.022 | 3.69E-06 | -0.051 | 0.064 | 0.43 | |
| *Parabacteroides* | rs17141986 | G | C | 0.050 | 0.011 | 7.17E-06 | 0.001 | 0.031 | 0.97 | |
| *Parabacteroides* | rs3860755 | G | C | 0.056 | 0.012 | 1.66E-06 | 0.000 | 0.032 | 1.00 | |
| *Parabacteroides* | rs4236095 | G | A | 0.076 | 0.016 | 1.22E-06 | 0.064 | 0.043 | 0.14 | |
| *Parabacteroides* | rs60884758 | C | T | -0.070 | 0.014 | 7.82E-07 | -0.007 | 0.039 | 0.86 | |
| *Parabacteroides* | rs6657302 | T | C | -0.105 | 0.023 | 3.58E-06 | 0.027 | 0.061 | 0.66 | |
| *Parabacteroides* | rs72893646 | A | T | -0.072 | 0.016 | 4.93E-06 | 0.002 | 0.041 | 0.96 | |
| *Parabacteroides* | rs7298818 | C | T | 0.089 | 0.020 | 9.68E-06 | 0.101 | 0.053 | 0.06 | |
| *Paraprevotella* | rs11881426 | T | A | -0.152 | 0.034 | 8.85E-06 | 0.085 | 0.057 | 0.13 | |
| *Paraprevotella* | rs13023298 | A | G | 0.104 | 0.024 | 9.08E-06 | 0.059 | 0.043 | 0.16 | |
| *Paraprevotella* | rs145020347 | A | G | -0.125 | 0.026 | 2.02E-06 | -0.058 | 0.049 | 0.23 | |
| *Paraprevotella* | rs17109926 | A | G | -0.099 | 0.022 | 4.83E-06 | -0.033 | 0.038 | 0.38 | |
| *Paraprevotella* | rs17785622 | A | G | 0.248 | 0.052 | 2.23E-06 | 0.040 | 0.063 | 0.52 | |
| *Paraprevotella* | rs2081023 | A | G | -0.123 | 0.024 | 2.19E-07 | -0.012 | 0.045 | 0.78 | |
| *Paraprevotella* | rs3008582 | T | C | 0.106 | 0.023 | 3.28E-06 | 0.025 | 0.042 | 0.56 | |
| *Paraprevotella* | rs3801748 | G | A | 0.078 | 0.017 | 5.59E-06 | -0.029 | 0.030 | 0.33 | |
| *Paraprevotella* | rs4756632 | G | T | -0.139 | 0.029 | 1.65E-06 | -0.008 | 0.054 | 0.89 | |
| *Paraprevotella* | rs4767113 | C | T | 0.088 | 0.018 | 1.58E-06 | 0.003 | 0.032 | 0.93 | |
| *Paraprevotella* | rs58117850 | C | A | -0.150 | 0.032 | 3.68E-06 | 0.027 | 0.058 | 0.63 | |
| *Paraprevotella* | rs77693755 | C | T | -0.142 | 0.031 | 4.26E-06 | 0.026 | 0.057 | 0.65 | |
| *Paraprevotella* | rs9602779 | A | C | -0.107 | 0.022 | 1.27E-06 | -0.040 | 0.037 | 0.27 | |
| *Paraprevotella* | rs9900242 | A | G | -0.085 | 0.018 | 1.13E-06 | 0.013 | 0.030 | 0.67 | |
| *Parasutterella* | rs10899911 | A | G | -0.072 | 0.015 | 1.30E-06 | -0.026 | 0.033 | 0.42 | |
| *Parasutterella* | rs11715853 | G | A | -0.066 | 0.015 | 5.70E-06 | -0.043 | 0.032 | 0.18 | |
| *Parasutterella* | rs1403396 | A | T | -0.076 | 0.016 | 1.95E-06 | 0.010 | 0.035 | 0.78 | |
| *Parasutterella* | rs1816916 | A | T | 0.088 | 0.020 | 9.21E-06 | 0.010 | 0.050 | 0.84 | |
| *Parasutterella* | rs2090816 | A | C | 0.084 | 0.018 | 2.11E-06 | 0.003 | 0.039 | 0.94 | |
| *Parasutterella* | rs2387977 | T | C | -0.068 | 0.013 | 4.21E-07 | 0.004 | 0.032 | 0.90 | |
| *Parasutterella* | rs35055552 | T | C | 0.110 | 0.024 | 3.27E-06 | -0.063 | 0.058 | 0.27 | |
| *Parasutterella* | rs35414597 | T | A | -0.068 | 0.014 | 1.44E-06 | 0.026 | 0.031 | 0.40 | |
| *Parasutterella* | rs4671249 | T | C | 0.147 | 0.032 | 5.24E-06 | 0.026 | 0.061 | 0.67 | |
| *Parasutterella* | rs55877868 | A | C | -0.104 | 0.023 | 4.66E-06 | -0.030 | 0.049 | 0.54 | |
| *Parasutterella* | rs60622892 | T | G | -0.148 | 0.032 | 4.81E-06 | 0.034 | 0.068 | 0.62 | |
| *Parasutterella* | rs62273907 | A | G | 0.229 | 0.050 | 4.91E-06 | 0.025 | 0.068 | 0.72 | |
| *Parasutterella* | rs6828768 | C | T | 0.064 | 0.013 | 1.58E-06 | 0.028 | 0.029 | 0.34 | |
| *Parasutterella* | rs7303158 | C | T | 0.065 | 0.013 | 1.45E-06 | 0.043 | 0.029 | 0.14 | |
| *Parasutterella* | rs7311004 | T | C | -0.062 | 0.014 | 6.01E-06 | -0.036 | 0.030 | 0.23 | |
| *Parasutterella* | rs7572229 | G | A | 0.066 | 0.013 | 5.95E-07 | 0.032 | 0.029 | 0.28 | |
| *Parasutterella* | rs78383039 | T | C | -0.146 | 0.030 | 8.46E-07 | 0.023 | 0.062 | 0.70 | |
| *Parasutterella* | rs8039785 | T | G | 0.062 | 0.013 | 3.33E-06 | -0.061 | 0.029 | 0.04 | |
| *Parasutterella* | rs823424 | G | A | -0.071 | 0.016 | 5.48E-06 | -0.016 | 0.035 | 0.65 | |
| *Parasutterella* | rs900566 | A | G | -0.059 | 0.013 | 9.92E-06 | 0.050 | 0.029 | 0.09 | |
| *Peptococcus* | rs10031059 | T | C | -0.121 | 0.023 | 8.09E-08 | -0.027 | 0.035 | 0.43 | |
| *Peptococcus* | rs11001948 | C | A | -0.191 | 0.038 | 5.35E-07 | -0.021 | 0.059 | 0.72 | |
| *Peptococcus* | rs11030569 | A | T | -0.174 | 0.037 | 3.30E-06 | 0.003 | 0.065 | 0.96 | |
| *Peptococcus* | rs2054133 | G | A | 0.090 | 0.019 | 1.99E-06 | 0.045 | 0.031 | 0.15 | |
| *Peptococcus* | rs34282744 | G | C | 0.192 | 0.040 | 1.61E-06 | -0.029 | 0.070 | 0.68 | |
| *Peptococcus* | rs36121075 | A | G | -0.141 | 0.031 | 4.37E-06 | -0.048 | 0.051 | 0.35 | |
| *Peptococcus* | rs413827 | G | A | 0.110 | 0.024 | 3.47E-06 | -0.077 | 0.036 | 0.03 | |
| *Peptococcus* | rs5770862 | T | C | 0.162 | 0.036 | 5.61E-06 | -0.040 | 0.056 | 0.47 | |
| *Peptococcus* | rs57905414 | T | A | 0.185 | 0.032 | 1.04E-08 | 0.112 | 0.055 | 0.04 | |
| *Peptococcus* | rs62424012 | G | A | 0.137 | 0.029 | 2.66E-06 | -0.105 | 0.048 | 0.03 | |
| *Peptococcus* | rs6821675 | C | A | 0.144 | 0.031 | 4.92E-06 | 0.036 | 0.048 | 0.45 | |
| *Peptococcus* | rs7033353 | T | G | 0.090 | 0.019 | 2.07E-06 | 0.005 | 0.030 | 0.85 | |
| *Peptococcus* | rs72850165 | T | C | -0.134 | 0.030 | 7.80E-06 | -0.032 | 0.048 | 0.51 | |
| *Peptococcus* | rs74592222 | G | A | 0.138 | 0.030 | 5.27E-06 | -0.025 | 0.047 | 0.60 | |
| *Peptococcus* | rs7766680 | G | C | 0.098 | 0.021 | 5.08E-06 | -0.014 | 0.032 | 0.67 | |
| *Peptococcus* | rs77681628 | C | T | 0.200 | 0.039 | 2.32E-07 | 0.145 | 0.061 | 0.02 | |
| *Phascolarctobacterium* | rs12618201 | A | G | 0.064 | 0.014 | 3.44E-06 | 0.016 | 0.030 | 0.61 | |
| *Phascolarctobacterium* | rs1264476 | T | G | 0.077 | 0.017 | 3.81E-06 | -0.011 | 0.036 | 0.75 | |
| *Phascolarctobacterium* | rs130483 | A | G | 0.066 | 0.014 | 5.07E-06 | -0.012 | 0.032 | 0.70 | |
| *Phascolarctobacterium* | rs139811148 | A | G | -0.088 | 0.020 | 9.86E-06 | -0.007 | 0.043 | 0.87 | |
| *Phascolarctobacterium* | rs55766124 | T | C | -0.168 | 0.036 | 3.54E-06 | 0.071 | 0.065 | 0.27 | |
| *Phascolarctobacterium* | rs56069061 | G | A | -0.111 | 0.023 | 1.40E-06 | 0.003 | 0.056 | 0.96 | |
| *Phascolarctobacterium* | rs56157888 | A | C | 0.095 | 0.019 | 8.54E-07 | -0.079 | 0.042 | 0.06 | |
| *Phascolarctobacterium* | rs6427992 | G | C | -0.065 | 0.014 | 2.09E-06 | -0.021 | 0.030 | 0.48 | |
| *Phascolarctobacterium* | rs74540770 | G | A | -0.121 | 0.026 | 2.88E-06 | -0.046 | 0.054 | 0.40 | |
| *Phascolarctobacterium* | rs75882962 | T | C | 0.097 | 0.019 | 3.72E-07 | -0.035 | 0.041 | 0.40 | |
| *Phascolarctobacterium* | rs76124218 | C | G | -0.159 | 0.034 | 3.74E-06 | 0.070 | 0.066 | 0.29 | |
| *Phascolarctobacterium* | rs7982713 | G | A | 0.073 | 0.016 | 8.42E-06 | -0.069 | 0.035 | 0.05 | |
| *Prevotella 7* | rs11035469 | A | G | -0.144 | 0.031 | 3.80E-06 | 0.042 | 0.039 | 0.28 | |
| *Prevotella 7* | rs12195431 | T | C | 0.197 | 0.044 | 8.87E-06 | -0.121 | 0.063 | 0.06 | |
| *Prevotella 7* | rs2240542 | C | T | 0.121 | 0.026 | 3.90E-06 | -0.082 | 0.033 | 0.01 | |
| *Prevotella 7* | rs2918132 | C | T | -0.115 | 0.025 | 6.88E-06 | -0.020 | 0.033 | 0.55 | |
| *Prevotella 7* | rs385483 | A | G | 0.137 | 0.029 | 2.67E-06 | 0.020 | 0.037 | 0.59 | |
| *Prevotella 7* | rs4879924 | C | T | -0.158 | 0.034 | 4.37E-06 | 0.031 | 0.042 | 0.46 | |
| *Prevotella 7* | rs57404562 | C | A | 0.155 | 0.032 | 8.67E-07 | 0.064 | 0.037 | 0.08 | |
| *Prevotella 7* | rs62026720 | C | T | -0.219 | 0.049 | 9.12E-06 | -0.080 | 0.057 | 0.16 | |
| *Prevotella 7* | rs9426434 | T | C | -0.124 | 0.028 | 9.00E-06 | 0.039 | 0.033 | 0.25 | |
| *Prevotella 7* | rs9608249 | A | G | -0.158 | 0.034 | 2.55E-06 | 0.103 | 0.043 | 0.02 | |
| *Prevotella 7* | rs9959718 | G | A | 0.133 | 0.028 | 1.36E-06 | 0.054 | 0.033 | 0.10 | |
| *Prevotella 9* | rs10512344 | C | G | 0.247 | 0.054 | 5.45E-06 | 0.084 | 0.073 | 0.25 | |
| *Prevotella 9* | rs11199734 | A | T | 0.077 | 0.017 | 5.49E-06 | -0.033 | 0.037 | 0.36 | |
| *Prevotella 9* | rs11685699 | C | T | -0.141 | 0.030 | 1.74E-06 | -0.066 | 0.058 | 0.25 | |
| *Prevotella 9* | rs117271932 | A | G | 0.208 | 0.044 | 2.30E-06 | 0.150 | 0.071 | 0.03 | |
| *Prevotella 9* | rs12648235 | T | C | 0.079 | 0.018 | 9.72E-06 | -0.114 | 0.043 | 0.01 | |
| *Prevotella 9* | rs1304512 | G | A | 0.076 | 0.017 | 4.41E-06 | -0.053 | 0.035 | 0.13 | |
| *Prevotella 9* | rs16966465 | G | C | 0.074 | 0.017 | 6.88E-06 | -0.065 | 0.036 | 0.07 | |
| *Prevotella 9* | rs2104588 | T | C | 0.106 | 0.024 | 8.98E-06 | 0.064 | 0.050 | 0.20 | |
| *Prevotella 9* | rs2495052 | A | G | 0.084 | 0.019 | 8.58E-06 | 0.039 | 0.040 | 0.33 | |
| *Prevotella 9* | rs2683313 | A | G | -0.072 | 0.015 | 1.76E-06 | -0.036 | 0.032 | 0.25 | |
| *Prevotella 9* | rs6853047 | T | C | 0.088 | 0.020 | 9.03E-06 | 0.003 | 0.044 | 0.95 | |
| *Prevotella 9* | rs7232121 | G | C | 0.067 | 0.014 | 3.25E-06 | -0.001 | 0.038 | 0.98 | |
| *Prevotella 9* | rs746764 | T | C | -0.092 | 0.019 | 2.15E-06 | 0.004 | 0.038 | 0.91 | |
| *Prevotella 9* | rs9428102 | A | G | -0.078 | 0.018 | 9.71E-06 | -0.002 | 0.036 | 0.95 | |
| *Prevotella 9* | rs9613013 | G | A | 0.092 | 0.020 | 5.99E-06 | -0.050 | 0.045 | 0.26 | |
| *Rikenellaceae (RC9 gut group)* | rs10078748 | G | T | -0.150 | 0.033 | 4.81E-06 | -0.029 | 0.040 | 0.46 | |
| *Rikenellaceae (RC9 gut group)* | rs11060474 | A | G | 0.179 | 0.040 | 9.52E-06 | 0.020 | 0.051 | 0.70 | |
| *Rikenellaceae (RC9 gut group)* | rs12501673 | A | G | 0.116 | 0.026 | 9.07E-06 | 0.024 | 0.032 | 0.44 | |
| *Rikenellaceae (RC9 gut group)* | rs17032291 | T | C | -0.170 | 0.037 | 4.13E-06 | -0.021 | 0.047 | 0.65 | |
| *Rikenellaceae (RC9 gut group)* | rs17582787 | A | G | -0.158 | 0.034 | 3.48E-06 | 0.032 | 0.041 | 0.44 | |
| *Rikenellaceae (RC9 gut group)* | rs2900503 | G | T | -0.172 | 0.033 | 1.33E-07 | -0.042 | 0.040 | 0.29 | |
| *Rikenellaceae (RC9 gut group)* | rs2998141 | T | C | -0.136 | 0.029 | 3.28E-06 | -0.046 | 0.039 | 0.23 | |
| *Rikenellaceae (RC9 gut group)* | rs4717843 | G | T | -0.119 | 0.026 | 4.61E-06 | -0.038 | 0.042 | 0.37 | |
| *Rikenellaceae (RC9 gut group)* | rs7113155 | G | C | 0.114 | 0.025 | 3.88E-06 | 0.009 | 0.029 | 0.76 | |
| *Rikenellaceae (RC9 gut group)* | rs7193937 | G | C | 0.124 | 0.028 | 7.63E-06 | -0.001 | 0.039 | 0.97 | |
| *Rikenellaceae (RC9 gut group)* | rs7712231 | A | G | 0.156 | 0.035 | 8.22E-06 | 0.056 | 0.041 | 0.17 | |
| *Rikenellaceae (RC9 gut group)* | rs80309088 | G | A | 0.174 | 0.038 | 5.64E-06 | 0.065 | 0.044 | 0.14 | |
| *Rikenellaceae (RC9 gut group)* | rs9887954 | G | A | -0.115 | 0.025 | 3.98E-06 | -0.036 | 0.030 | 0.23 | |
| *Romboutsia* | rs10279978 | A | G | -0.062 | 0.013 | 1.10E-06 | 0.034 | 0.034 | 0.32 | |
| *Romboutsia* | rs11221428 | T | C | -0.073 | 0.016 | 4.39E-06 | 0.026 | 0.038 | 0.51 | |
| *Romboutsia* | rs114398731 | G | C | -0.131 | 0.029 | 8.20E-06 | 0.086 | 0.069 | 0.21 | |
| *Romboutsia* | rs16843578 | C | T | -0.088 | 0.020 | 8.67E-06 | 0.102 | 0.051 | 0.04 | |
| *Romboutsia* | rs28603357 | T | C | -0.215 | 0.047 | 5.98E-06 | 0.025 | 0.079 | 0.75 | |
| *Romboutsia* | rs34302036 | A | G | 0.055 | 0.012 | 5.27E-06 | -0.013 | 0.029 | 0.67 | |
| *Romboutsia* | rs61841503 | G | A | 0.093 | 0.017 | 6.04E-08 | -0.005 | 0.039 | 0.90 | |
| *Romboutsia* | rs62504452 | A | G | -0.071 | 0.016 | 5.83E-06 | -0.048 | 0.039 | 0.21 | |
| *Romboutsia* | rs7109293 | A | G | 0.092 | 0.021 | 7.92E-06 | 0.100 | 0.061 | 0.10 | |
| *Romboutsia* | rs74180876 | C | T | 0.086 | 0.019 | 9.52E-06 | 0.056 | 0.044 | 0.20 | |
| *Romboutsia* | rs75200530 | T | G | -0.191 | 0.042 | 5.96E-06 | 0.004 | 0.068 | 0.95 | |
| *Romboutsia* | rs75987356 | G | A | -0.130 | 0.028 | 3.83E-06 | 0.001 | 0.069 | 0.99 | |
| *Romboutsia* | rs77702691 | A | G | -0.094 | 0.021 | 5.99E-06 | 0.054 | 0.051 | 0.28 | |
| *Romboutsia* | rs9389266 | T | G | 0.072 | 0.016 | 8.50E-06 | 0.018 | 0.040 | 0.65 | |
| *Romboutsia* | rs9567264 | C | T | 0.058 | 0.013 | 5.38E-06 | 0.021 | 0.032 | 0.51 | |
| *Roseburia* | rs11632648 | C | A | -0.053 | 0.012 | 8.61E-06 | 0.035 | 0.035 | 0.31 | |
| *Roseburia* | rs12740451 | T | C | 0.070 | 0.015 | 5.60E-06 | 0.017 | 0.044 | 0.69 | |
| *Roseburia* | rs16910295 | T | C | -0.098 | 0.021 | 2.89E-06 | -0.019 | 0.062 | 0.76 | |
| *Roseburia* | rs2034589 | G | C | 0.063 | 0.012 | 3.40E-07 | -0.009 | 0.037 | 0.80 | |
| *Roseburia* | rs2160994 | T | C | 0.055 | 0.011 | 9.79E-07 | -0.034 | 0.032 | 0.28 | |
| *Roseburia* | rs2943022 | T | C | 0.049 | 0.011 | 3.75E-06 | 0.025 | 0.029 | 0.40 | |
| *Roseburia* | rs302266 | T | C | -0.078 | 0.017 | 7.01E-06 | -0.061 | 0.047 | 0.20 | |
| *Roseburia* | rs329182 | T | C | 0.069 | 0.015 | 6.32E-06 | 0.004 | 0.042 | 0.93 | |
| *Roseburia* | rs4748237 | G | C | 0.049 | 0.011 | 4.45E-06 | -0.011 | 0.029 | 0.71 | |
| *Roseburia* | rs55858165 | A | C | 0.179 | 0.040 | 9.54E-06 | 0.095 | 0.074 | 0.20 | |
| *Roseburia* | rs56058612 | T | C | -0.065 | 0.015 | 9.85E-06 | -0.009 | 0.041 | 0.82 | |
| *Roseburia* | rs6445851 | G | A | -0.050 | 0.011 | 4.26E-06 | 0.046 | 0.030 | 0.12 | |
| *Roseburia* | rs6930661 | C | T | -0.096 | 0.020 | 2.72E-06 | 0.072 | 0.058 | 0.22 | |
| *Roseburia* | rs75326254 | C | T | -0.105 | 0.023 | 5.86E-06 | -0.149 | 0.062 | 0.02 | |
| *Roseburia* | rs78753150 | A | C | 0.097 | 0.021 | 6.03E-06 | -0.011 | 0.060 | 0.86 | |
| *Roseburia* | rs9300744 | C | T | -0.059 | 0.013 | 3.13E-06 | -0.045 | 0.034 | 0.18 | |
| *Ruminiclostridium 5* | rs10827477 | A | G | -0.055 | 0.012 | 2.00E-06 | 0.027 | 0.031 | 0.38 | |
| *Ruminiclostridium 5* | rs113753996 | T | C | 0.082 | 0.017 | 2.55E-06 | 0.044 | 0.050 | 0.38 | |
| *Ruminiclostridium 5* | rs1223978 | T | C | 0.048 | 0.011 | 7.73E-06 | -0.039 | 0.030 | 0.20 | |
| *Ruminiclostridium 5* | rs1492620 | T | C | -0.083 | 0.018 | 3.99E-06 | 0.026 | 0.048 | 0.58 | |
| *Ruminiclostridium 5* | rs2286384 | G | C | -0.052 | 0.011 | 1.39E-06 | 0.026 | 0.029 | 0.38 | |
| *Ruminiclostridium 5* | rs243585 | C | G | -0.059 | 0.012 | 1.29E-06 | -0.012 | 0.037 | 0.74 | |
| *Ruminiclostridium 5* | rs2503704 | T | C | 0.052 | 0.012 | 9.38E-06 | -0.012 | 0.036 | 0.75 | |
| *Ruminiclostridium 5* | rs2791343 | T | C | 0.052 | 0.011 | 5.07E-06 | -0.010 | 0.031 | 0.74 | |
| *Ruminiclostridium 5* | rs2801960 | C | G | 0.052 | 0.012 | 6.02E-06 | 0.027 | 0.032 | 0.38 | |
| *Ruminiclostridium 5* | rs2833828 | G | A | 0.049 | 0.011 | 6.66E-06 | 0.017 | 0.029 | 0.57 | |
| *Ruminiclostridium 5* | rs6121460 | G | A | 0.093 | 0.020 | 2.82E-06 | 0.045 | 0.068 | 0.51 | |
| *Ruminiclostridium 5* | rs79968837 | A | G | -0.095 | 0.019 | 9.06E-07 | -0.052 | 0.053 | 0.33 | |
| *Ruminiclostridium 5* | rs8053158 | A | G | -0.074 | 0.016 | 3.27E-06 | 0.014 | 0.052 | 0.79 | |
| *Ruminiclostridium 6* | rs10829821 | T | C | -0.098 | 0.022 | 6.26E-06 | -0.010 | 0.053 | 0.86 | |
| *Ruminiclostridium 6* | rs116969552 | A | G | -0.167 | 0.038 | 9.48E-06 | -0.137 | 0.074 | 0.06 | |
| *Ruminiclostridium 6* | rs11992182 | A | C | 0.063 | 0.014 | 5.75E-06 | 0.034 | 0.033 | 0.30 | |
| *Ruminiclostridium 6* | rs12362316 | A | G | 0.074 | 0.015 | 1.48E-06 | 0.026 | 0.037 | 0.48 | |
| *Ruminiclostridium 6* | rs12595638 | A | G | -0.104 | 0.023 | 8.27E-06 | 0.016 | 0.055 | 0.77 | |
| *Ruminiclostridium 6* | rs1756364 | G | T | 0.100 | 0.020 | 4.03E-07 | 0.062 | 0.056 | 0.26 | |
| *Ruminiclostridium 6* | rs2548459 | C | T | 0.055 | 0.012 | 6.18E-06 | -0.018 | 0.029 | 0.54 | |
| *Ruminiclostridium 6* | rs56212330 | A | G | 0.059 | 0.013 | 8.68E-06 | -0.016 | 0.031 | 0.61 | |
| *Ruminiclostridium 6* | rs589368 | G | A | -0.147 | 0.033 | 8.81E-06 | -0.071 | 0.065 | 0.27 | |
| *Ruminiclostridium 6* | rs61060922 | T | G | 0.159 | 0.032 | 7.91E-07 | 0.187 | 0.064 | 0.00 | |
| *Ruminiclostridium 6* | rs67479537 | T | C | 0.119 | 0.026 | 7.04E-06 | -0.026 | 0.068 | 0.70 | |
| *Ruminiclostridium 6* | rs71414120 | T | G | 0.201 | 0.041 | 7.70E-07 | -0.069 | 0.078 | 0.37 | |
| *Ruminiclostridium 6* | rs72991535 | T | G | 0.136 | 0.030 | 4.35E-06 | 0.067 | 0.070 | 0.34 | |
| *Ruminiclostridium 6* | rs792058 | G | A | 0.055 | 0.013 | 9.92E-06 | -0.037 | 0.032 | 0.25 | |
| *Ruminiclostridium 6* | rs79968172 | G | A | 0.116 | 0.024 | 1.77E-06 | -0.055 | 0.061 | 0.36 | |
| *Ruminiclostridium 6* | rs9555756 | A | C | -0.080 | 0.018 | 5.34E-06 | -0.008 | 0.042 | 0.85 | |
| *Ruminiclostridium 9* | rs113048721 | C | G | 0.060 | 0.013 | 6.02E-06 | -0.010 | 0.034 | 0.77 | |
| *Ruminiclostridium 9* | rs115044523 | G | A | -0.098 | 0.020 | 1.32E-06 | 0.016 | 0.052 | 0.76 | |
| *Ruminiclostridium 9* | rs12040548 | G | T | 0.057 | 0.012 | 3.13E-06 | 0.030 | 0.032 | 0.36 | |
| *Ruminiclostridium 9* | rs12419854 | T | A | -0.073 | 0.016 | 2.89E-06 | 0.043 | 0.043 | 0.32 | |
| *Ruminiclostridium 9* | rs13033315 | T | A | 0.051 | 0.011 | 4.47E-06 | 0.084 | 0.029 | 0.00 | |
| *Ruminiclostridium 9* | rs57665991 | C | G | -0.064 | 0.012 | 1.98E-07 | -0.041 | 0.032 | 0.20 | |
| *Ruminiclostridium 9* | rs6082461 | A | C | 0.059 | 0.013 | 7.59E-06 | 0.025 | 0.035 | 0.48 | |
| *Ruminiclostridium 9* | rs6686918 | A | G | -0.055 | 0.012 | 8.94E-06 | -0.003 | 0.033 | 0.94 | |
| *Ruminiclostridium 9* | rs68063877 | G | A | -0.070 | 0.016 | 7.67E-06 | -0.048 | 0.041 | 0.25 | |
| *Ruminiclostridium 9* | rs7137760 | C | T | 0.051 | 0.011 | 5.95E-06 | -0.014 | 0.029 | 0.64 | |
| *Ruminiclostridium 9* | rs74303178 | T | C | 0.053 | 0.012 | 7.95E-06 | 0.009 | 0.032 | 0.78 | |
| *Ruminiclostridium 9* | rs78191726 | T | C | 0.094 | 0.021 | 6.94E-06 | 0.035 | 0.051 | 0.49 | |
| *Ruminiclostridium 9* | rs79082720 | C | G | 0.093 | 0.020 | 5.83E-06 | -0.057 | 0.058 | 0.32 | |
| *Ruminiclostridium 9* | rs918449 | A | G | -0.095 | 0.020 | 1.42E-06 | 0.002 | 0.056 | 0.96 | |
| *Ruminiclostridium 9* | rs9522712 | T | C | 0.070 | 0.015 | 6.30E-06 | -0.006 | 0.040 | 0.87 | |
| *Ruminiclostridium 9* | rs9809789 | C | T | -0.072 | 0.016 | 7.01E-06 | 0.031 | 0.042 | 0.46 | |
| *Ruminococcaceae (NK4A214 group)* | rs11241747 | C | T | 0.053 | 0.012 | 8.69E-06 | 0.010 | 0.034 | 0.77 | |
| *Ruminococcaceae (NK4A214 group)* | rs114244418 | C | G | -0.175 | 0.037 | 2.58E-06 | 0.112 | 0.071 | 0.12 | |
| *Ruminococcaceae (NK4A214 group)* | rs11586410 | G | A | -0.086 | 0.017 | 3.76E-07 | -0.054 | 0.048 | 0.26 | |
| *Ruminococcaceae (NK4A214 group)* | rs12642039 | T | C | -0.055 | 0.012 | 3.63E-06 | 0.019 | 0.031 | 0.54 | |
| *Ruminococcaceae (NK4A214 group)* | rs12731 | A | G | -0.053 | 0.012 | 4.51E-06 | -0.002 | 0.029 | 0.96 | |
| *Ruminococcaceae (NK4A214 group)* | rs13087692 | T | G | 0.057 | 0.013 | 5.05E-06 | -0.051 | 0.031 | 0.10 | |
| *Ruminococcaceae (NK4A214 group)* | rs136761 | G | A | -0.059 | 0.012 | 8.19E-07 | 0.013 | 0.032 | 0.70 | |
| *Ruminococcaceae (NK4A214 group)* | rs147475196 | A | G | -0.134 | 0.030 | 5.86E-06 | 0.010 | 0.073 | 0.89 | |
| *Ruminococcaceae (NK4A214 group)* | rs3087587 | A | G | -0.051 | 0.012 | 9.92E-06 | -0.014 | 0.030 | 0.65 | |
| *Ruminococcaceae (NK4A214 group)* | rs34576931 | G | C | -0.087 | 0.019 | 7.42E-06 | 0.049 | 0.049 | 0.31 | |
| *Ruminococcaceae (NK4A214 group)* | rs35559912 | T | C | -0.093 | 0.020 | 5.57E-06 | -0.037 | 0.057 | 0.52 | |
| *Ruminococcaceae (NK4A214 group)* | rs4814689 | C | T | -0.108 | 0.023 | 2.68E-06 | -0.042 | 0.052 | 0.42 | |
| *Ruminococcaceae (NK4A214 group)* | rs5994253 | A | G | -0.081 | 0.016 | 2.64E-07 | -0.003 | 0.047 | 0.95 | |
| *Ruminococcaceae (NK4A214 group)* | rs62027366 | T | C | 0.062 | 0.014 | 7.75E-06 | 0.020 | 0.036 | 0.59 | |
| *Ruminococcaceae (NK4A214 group)* | rs73158814 | C | G | -0.109 | 0.023 | 1.56E-06 | 0.063 | 0.060 | 0.30 | |
| *Ruminococcaceae (NK4A214 group)* | rs7573569 | T | C | 0.108 | 0.023 | 4.00E-06 | 0.083 | 0.063 | 0.19 | |
| *Ruminococcaceae (UCG002)* | rs10916131 | C | T | -0.069 | 0.015 | 2.31E-06 | 0.015 | 0.041 | 0.71 | |
| *Ruminococcaceae (UCG002)* | rs10927423 | C | A | -0.071 | 0.015 | 1.36E-06 | -0.021 | 0.047 | 0.66 | |
| *Ruminococcaceae (UCG002)* | rs11078171 | C | G | 0.050 | 0.011 | 8.98E-06 | -0.049 | 0.030 | 0.10 | |
| *Ruminococcaceae (UCG002)* | rs113147300 | A | G | -0.076 | 0.016 | 4.05E-06 | -0.028 | 0.042 | 0.50 | |
| *Ruminococcaceae (UCG002)* | rs11607472 | A | G | -0.078 | 0.018 | 9.65E-06 | 0.051 | 0.047 | 0.28 | |
| *Ruminococcaceae (UCG002)* | rs116974815 | C | A | -0.190 | 0.040 | 1.72E-06 | 0.052 | 0.079 | 0.51 | |
| *Ruminococcaceae (UCG002)* | rs12463378 | A | G | -0.052 | 0.011 | 3.20E-06 | 0.012 | 0.030 | 0.69 | |
| *Ruminococcaceae (UCG002)* | rs2265670 | C | G | -0.051 | 0.011 | 2.75E-06 | 0.031 | 0.029 | 0.28 | |
| *Ruminococcaceae (UCG002)* | rs362417 | G | C | -0.055 | 0.012 | 5.77E-06 | -0.015 | 0.032 | 0.65 | |
| *Ruminococcaceae (UCG002)* | rs55793120 | T | C | 0.137 | 0.027 | 5.39E-07 | -0.145 | 0.076 | 0.05 | |
| *Ruminococcaceae (UCG002)* | rs56030423 | G | A | -0.098 | 0.022 | 5.54E-06 | 0.004 | 0.056 | 0.94 | |
| *Ruminococcaceae (UCG002)* | rs57079348 | T | G | -0.077 | 0.017 | 9.39E-06 | -0.023 | 0.046 | 0.62 | |
| *Ruminococcaceae (UCG002)* | rs62374283 | T | C | -0.058 | 0.012 | 1.45E-06 | -0.013 | 0.033 | 0.70 | |
| *Ruminococcaceae (UCG002)* | rs6542556 | A | G | 0.051 | 0.011 | 7.86E-06 | 0.093 | 0.031 | 0.00 | |
| *Ruminococcaceae (UCG002)* | rs67746927 | C | G | -0.054 | 0.011 | 8.89E-07 | 0.028 | 0.030 | 0.35 | |
| *Ruminococcaceae (UCG002)* | rs6793778 | C | T | -0.056 | 0.013 | 8.18E-06 | -0.069 | 0.034 | 0.04 | |
| *Ruminococcaceae (UCG002)* | rs7120052 | A | C | 0.062 | 0.014 | 4.02E-06 | 0.010 | 0.038 | 0.78 | |
| *Ruminococcaceae (UCG002)* | rs7155595 | C | A | 0.057 | 0.012 | 1.11E-06 | -0.041 | 0.031 | 0.18 | |
| *Ruminococcaceae (UCG002)* | rs7249614 | A | G | -0.049 | 0.011 | 8.70E-06 | 0.035 | 0.039 | 0.37 | |
| *Ruminococcaceae (UCG002)* | rs72874194 | G | C | -0.077 | 0.017 | 4.11E-06 | 0.076 | 0.049 | 0.12 | |
| *Ruminococcaceae (UCG002)* | rs7342369 | C | A | -0.053 | 0.012 | 5.39E-06 | -0.001 | 0.032 | 0.98 | |
| *Ruminococcaceae (UCG002)* | rs76847269 | A | G | 0.164 | 0.036 | 4.41E-06 | -0.032 | 0.063 | 0.62 | |
| *Ruminococcaceae (UCG002)* | rs77564310 | A | C | -0.071 | 0.014 | 4.14E-07 | 0.003 | 0.036 | 0.94 | |
| *Ruminococcaceae (UCG002)* | rs7898496 | T | C | 0.081 | 0.018 | 9.23E-06 | -0.022 | 0.051 | 0.67 | |
| *Ruminococcaceae (UCG002)* | rs79016051 | C | T | -0.089 | 0.019 | 2.78E-06 | -0.132 | 0.053 | 0.01 | |
| *Ruminococcaceae (UCG002)* | rs882348 | A | G | -0.080 | 0.018 | 7.52E-06 | 0.055 | 0.052 | 0.29 | |
| *Ruminococcaceae (UCG003)* | rs10490280 | C | T | -0.067 | 0.014 | 2.73E-06 | -0.054 | 0.035 | 0.12 | |
| *Ruminococcaceae (UCG003)* | rs11243416 | T | C | -0.093 | 0.019 | 1.29E-06 | 0.004 | 0.050 | 0.93 | |
| *Ruminococcaceae (UCG003)* | rs11831525 | A | C | 0.071 | 0.015 | 2.71E-06 | 0.014 | 0.040 | 0.72 | |
| *Ruminococcaceae (UCG003)* | rs139730 | G | C | -0.058 | 0.013 | 9.80E-06 | 0.014 | 0.033 | 0.67 | |
| *Ruminococcaceae (UCG003)* | rs16959793 | A | C | -0.063 | 0.013 | 1.90E-06 | -0.006 | 0.033 | 0.87 | |
| *Ruminococcaceae (UCG003)* | rs2523124 | T | C | -0.055 | 0.012 | 6.06E-06 | -0.039 | 0.030 | 0.20 | |
| *Ruminococcaceae (UCG003)* | rs2686073 | G | A | -0.057 | 0.013 | 8.43E-06 | -0.009 | 0.031 | 0.76 | |
| *Ruminococcaceae (UCG003)* | rs3013089 | G | A | -0.055 | 0.012 | 4.60E-06 | 0.014 | 0.030 | 0.64 | |
| *Ruminococcaceae (UCG003)* | rs4452755 | A | C | -0.063 | 0.013 | 2.49E-06 | 0.034 | 0.033 | 0.31 | |
| *Ruminococcaceae (UCG003)* | rs4532474 | G | A | 0.077 | 0.017 | 6.39E-06 | 0.073 | 0.044 | 0.10 | |
| *Ruminococcaceae (UCG003)* | rs4629039 | T | A | 0.055 | 0.012 | 6.35E-06 | 0.008 | 0.031 | 0.80 | |
| *Ruminococcaceae (UCG003)* | rs61977762 | T | C | -0.138 | 0.031 | 9.85E-06 | 0.038 | 0.065 | 0.56 | |
| *Ruminococcaceae (UCG003)* | rs646327 | G | A | 0.059 | 0.012 | 7.18E-07 | -0.018 | 0.029 | 0.55 | |
| *Ruminococcaceae (UCG003)* | rs6759615 | A | G | 0.103 | 0.020 | 3.04E-07 | 0.008 | 0.056 | 0.88 | |
| *Ruminococcaceae (UCG003)* | rs73341548 | T | G | -0.170 | 0.032 | 9.50E-08 | 0.089 | 0.068 | 0.19 | |
| *Ruminococcaceae (UCG003)* | rs78720113 | A | G | -0.115 | 0.025 | 3.81E-06 | -0.048 | 0.071 | 0.50 | |
| *Ruminococcaceae (UCG004)* | rs10976229 | T | G | 0.096 | 0.021 | 7.70E-06 | -0.025 | 0.042 | 0.55 | |
| *Ruminococcaceae (UCG004)* | rs12125734 | G | T | 0.134 | 0.026 | 1.95E-07 | 0.023 | 0.048 | 0.63 | |
| *Ruminococcaceae (UCG004)* | rs2248146 | T | C | 0.069 | 0.015 | 7.22E-06 | 0.042 | 0.031 | 0.18 | |
| *Ruminococcaceae (UCG004)* | rs3800178 | C | T | -0.080 | 0.018 | 6.15E-06 | -0.001 | 0.036 | 0.99 | |
| *Ruminococcaceae (UCG004)* | rs516741 | T | G | -0.076 | 0.016 | 3.09E-06 | 0.002 | 0.033 | 0.96 | |
| *Ruminococcaceae (UCG004)* | rs61926359 | A | G | 0.092 | 0.021 | 7.11E-06 | 0.044 | 0.040 | 0.28 | |
| *Ruminococcaceae (UCG004)* | rs6769553 | A | G | 0.085 | 0.016 | 6.78E-08 | -0.056 | 0.032 | 0.08 | |
| *Ruminococcaceae (UCG004)* | rs7566377 | A | G | -0.154 | 0.034 | 7.65E-06 | 0.058 | 0.080 | 0.46 | |
| *Ruminococcaceae (UCG004)* | rs7569771 | A | G | -0.076 | 0.017 | 8.29E-06 | 0.032 | 0.036 | 0.38 | |
| *Ruminococcaceae (UCG004)* | rs9818949 | G | T | 0.086 | 0.019 | 5.17E-06 | -0.025 | 0.043 | 0.56 | |
| *Ruminococcaceae (UCG005)* | rs10873449 | T | C | 0.065 | 0.014 | 5.41E-06 | -0.041 | 0.039 | 0.29 | |
| *Ruminococcaceae (UCG005)* | rs10937802 | G | A | 0.076 | 0.017 | 7.03E-06 | -0.011 | 0.049 | 0.83 | |
| *Ruminococcaceae (UCG005)* | rs10950694 | T | C | 0.058 | 0.011 | 4.16E-07 | -0.021 | 0.029 | 0.47 | |
| *Ruminococcaceae (UCG005)* | rs114279581 | A | G | -0.147 | 0.032 | 3.50E-06 | 0.083 | 0.062 | 0.18 | |
| *Ruminococcaceae (UCG005)* | rs12288512 | A | G | 0.067 | 0.014 | 3.91E-06 | 0.019 | 0.038 | 0.61 | |
| *Ruminococcaceae (UCG005)* | rs12604884 | G | T | 0.068 | 0.014 | 2.63E-06 | -0.035 | 0.039 | 0.36 | |
| *Ruminococcaceae (UCG005)* | rs2893871 | G | A | -0.074 | 0.016 | 2.17E-06 | 0.034 | 0.040 | 0.40 | |
| *Ruminococcaceae (UCG005)* | rs34781347 | G | A | 0.189 | 0.039 | 1.05E-06 | 0.052 | 0.070 | 0.45 | |
| *Ruminococcaceae (UCG005)* | rs35166120 | C | G | -0.069 | 0.015 | 2.71E-06 | 0.065 | 0.040 | 0.10 | |
| *Ruminococcaceae (UCG005)* | rs394449 | A | T | 0.069 | 0.015 | 3.17E-06 | 0.037 | 0.038 | 0.33 | |
| *Ruminococcaceae (UCG005)* | rs60081663 | C | G | 0.158 | 0.032 | 7.56E-07 | 0.050 | 0.065 | 0.44 | |
| *Ruminococcaceae (UCG005)* | rs7449320 | C | A | 0.060 | 0.013 | 4.66E-06 | 0.038 | 0.035 | 0.28 | |
| *Ruminococcaceae (UCG005)* | rs7495495 | A | G | 0.088 | 0.019 | 6.83E-06 | -0.112 | 0.064 | 0.08 | |
| *Ruminococcaceae (UCG005)* | rs7555878 | A | G | 0.059 | 0.013 | 2.83E-06 | 0.038 | 0.034 | 0.26 | |
| *Ruminococcaceae (UCG005)* | rs7586445 | G | A | 0.078 | 0.018 | 9.27E-06 | 0.013 | 0.041 | 0.74 | |
| *Ruminococcaceae (UCG009)* | rs111304297 | G | T | 0.073 | 0.016 | 8.41E-06 | 0.028 | 0.032 | 0.37 | |
| *Ruminococcaceae (UCG009)* | rs113006825 | T | C | -0.093 | 0.021 | 8.00E-06 | 0.092 | 0.036 | 0.01 | |
| *Ruminococcaceae (UCG009)* | rs12508214 | C | T | -0.077 | 0.017 | 4.53E-06 | -0.025 | 0.032 | 0.44 | |
| *Ruminococcaceae (UCG009)* | rs1550196 | G | A | 0.131 | 0.026 | 6.20E-07 | -0.051 | 0.051 | 0.32 | |
| *Ruminococcaceae (UCG009)* | rs2058609 | A | G | 0.082 | 0.017 | 2.97E-06 | -0.009 | 0.031 | 0.77 | |
| *Ruminococcaceae (UCG009)* | rs2192926 | A | G | -0.089 | 0.019 | 3.95E-06 | -0.015 | 0.034 | 0.65 | |
| *Ruminococcaceae (UCG009)* | rs4079028 | C | T | 0.092 | 0.020 | 4.37E-06 | -0.023 | 0.036 | 0.52 | |
| *Ruminococcaceae (UCG009)* | rs4708333 | T | G | -0.084 | 0.017 | 1.52E-06 | -0.013 | 0.032 | 0.68 | |
| *Ruminococcaceae (UCG009)* | rs61779334 | G | C | -0.138 | 0.029 | 2.25E-06 | -0.027 | 0.050 | 0.60 | |
| *Ruminococcaceae (UCG009)* | rs758191 | T | G | 0.177 | 0.038 | 2.37E-06 | 0.110 | 0.071 | 0.12 | |
| *Ruminococcaceae (UCG009)* | rs8009993 | G | C | -0.136 | 0.024 | 2.83E-08 | -0.046 | 0.050 | 0.35 | |
| *Ruminococcaceae (UCG009)* | rs9558661 | T | C | -0.090 | 0.020 | 7.73E-06 | -0.023 | 0.037 | 0.54 | |
| *Ruminococcaceae (UCG010)* | rs12597105 | G | A | 0.067 | 0.014 | 3.40E-06 | -0.030 | 0.033 | 0.37 | |
| *Ruminococcaceae (UCG010)* | rs2820282 | A | C | -0.059 | 0.013 | 2.55E-06 | 0.027 | 0.031 | 0.38 | |
| *Ruminococcaceae (UCG010)* | rs35506912 | G | C | -0.069 | 0.015 | 2.73E-06 | 0.002 | 0.037 | 0.95 | |
| *Ruminococcaceae (UCG010)* | rs5996509 | G | A | -0.126 | 0.028 | 7.84E-06 | -0.099 | 0.069 | 0.15 | |
| *Ruminococcaceae (UCG010)* | rs682403 | A | G | -0.059 | 0.012 | 2.39E-06 | -0.018 | 0.029 | 0.53 | |
| *Ruminococcaceae (UCG010)* | rs6958419 | C | T | -0.059 | 0.012 | 2.79E-06 | 0.040 | 0.029 | 0.17 | |
| *Ruminococcaceae (UCG010)* | rs73218807 | G | A | -0.166 | 0.037 | 6.26E-06 | 0.066 | 0.077 | 0.39 | |
| *Ruminococcaceae (UCG010)* | rs7441445 | C | T | -0.057 | 0.013 | 6.74E-06 | -0.035 | 0.030 | 0.24 | |
| *Ruminococcaceae (UCG010)* | rs7935775 | A | T | -0.063 | 0.014 | 4.73E-06 | -0.016 | 0.033 | 0.62 | |
| *Ruminococcaceae (UCG011)* | rs10274562 | C | T | 0.111 | 0.024 | 5.75E-06 | -0.007 | 0.031 | 0.83 | |
| *Ruminococcaceae (UCG011)* | rs12636310 | G | A | 0.133 | 0.028 | 2.53E-06 | 0.035 | 0.036 | 0.32 | |
| *Ruminococcaceae (UCG011)* | rs12724320 | C | T | -0.121 | 0.025 | 1.23E-06 | -0.028 | 0.031 | 0.36 | |
| *Ruminococcaceae (UCG011)* | rs1416041 | A | C | -0.182 | 0.034 | 8.12E-08 | -0.036 | 0.043 | 0.41 | |
| *Ruminococcaceae (UCG011)* | rs2729556 | C | T | -0.109 | 0.023 | 3.04E-06 | -0.021 | 0.030 | 0.48 | |
| *Ruminococcaceae (UCG011)* | rs4490371 | T | C | -0.112 | 0.025 | 7.08E-06 | -0.003 | 0.031 | 0.93 | |
| *Ruminococcaceae (UCG011)* | rs4973017 | G | A | 0.215 | 0.047 | 5.71E-06 | -0.084 | 0.072 | 0.24 | |
| *Ruminococcaceae (UCG011)* | rs79113084 | C | T | -0.152 | 0.032 | 1.65E-06 | -0.023 | 0.042 | 0.59 | |
| *Ruminococcaceae (UCG011)* | rs9322545 | C | G | -0.110 | 0.025 | 8.61E-06 | 0.032 | 0.031 | 0.29 | |
| *Ruminococcaceae (UCG011)* | rs9729514 | A | G | 0.185 | 0.039 | 2.78E-06 | -0.008 | 0.051 | 0.87 | |
| *Ruminococcaceae (UCG013)* | rs10877955 | A | G | -0.107 | 0.023 | 3.99E-06 | -0.073 | 0.059 | 0.21 | |
| *Ruminococcaceae (UCG013)* | rs11581881 | C | T | 0.066 | 0.014 | 4.92E-06 | -0.008 | 0.044 | 0.85 | |
| *Ruminococcaceae (UCG013)* | rs12189346 | G | A | 0.068 | 0.015 | 2.54E-06 | -0.064 | 0.043 | 0.14 | |
| *Ruminococcaceae (UCG013)* | rs12336782 | T | C | -0.086 | 0.019 | 6.13E-06 | 0.029 | 0.053 | 0.58 | |
| *Ruminococcaceae (UCG013)* | rs12485353 | G | A | -0.061 | 0.013 | 3.39E-06 | -0.040 | 0.036 | 0.26 | |
| *Ruminococcaceae (UCG013)* | rs12781711 | C | T | -0.066 | 0.012 | 2.33E-08 | 0.016 | 0.031 | 0.61 | |
| *Ruminococcaceae (UCG013)* | rs16918863 | A | C | 0.111 | 0.024 | 3.44E-06 | 0.074 | 0.060 | 0.22 | |
| *Ruminococcaceae (UCG013)* | rs1729063 | G | C | -0.053 | 0.012 | 1.00E-05 | -0.007 | 0.039 | 0.86 | |
| *Ruminococcaceae (UCG013)* | rs2428106 | C | G | -0.049 | 0.011 | 7.96E-06 | 0.021 | 0.029 | 0.48 | |
| *Ruminococcaceae (UCG013)* | rs2730183 | G | A | -0.049 | 0.011 | 8.73E-06 | 0.039 | 0.029 | 0.18 | |
| *Ruminococcaceae (UCG013)* | rs4385846 | G | T | 0.060 | 0.013 | 5.63E-06 | 0.027 | 0.035 | 0.44 | |
| *Ruminococcaceae (UCG013)* | rs75088940 | T | C | -0.094 | 0.020 | 2.63E-06 | 0.006 | 0.053 | 0.91 | |
| *Ruminococcaceae (UCG013)* | rs76973485 | G | T | 0.195 | 0.042 | 3.13E-06 | 0.036 | 0.068 | 0.59 | |
| *Ruminococcaceae (UCG013)* | rs7784330 | G | A | -0.050 | 0.011 | 8.72E-06 | 0.004 | 0.030 | 0.88 | |
| *Ruminococcaceae (UCG013)* | rs9313055 | T | C | 0.105 | 0.023 | 7.39E-06 | -0.004 | 0.066 | 0.95 | |
| *Ruminococcaceae (UCG013)* | rs9499480 | G | A | 0.056 | 0.013 | 9.39E-06 | -0.058 | 0.034 | 0.08 | |
| *Ruminococcaceae (UCG013)* | rs9565219 | T | A | -0.052 | 0.012 | 8.31E-06 | 0.050 | 0.031 | 0.11 | |
| *Ruminococcaceae (UCG014)* | rs10791168 | A | G | -0.066 | 0.015 | 9.48E-06 | 0.011 | 0.040 | 0.79 | |
| *Ruminococcaceae (UCG014)* | rs10941294 | C | T | -0.122 | 0.026 | 2.68E-06 | 0.107 | 0.061 | 0.08 | |
| *Ruminococcaceae (UCG014)* | rs112829527 | G | A | 0.121 | 0.027 | 6.79E-06 | -0.016 | 0.065 | 0.80 | |
| *Ruminococcaceae (UCG014)* | rs115777838 | T | C | -0.188 | 0.039 | 1.11E-06 | 0.018 | 0.073 | 0.80 | |
| *Ruminococcaceae (UCG014)* | rs12638134 | T | G | 0.058 | 0.012 | 1.12E-06 | -0.013 | 0.029 | 0.65 | |
| *Ruminococcaceae (UCG014)* | rs17296933 | C | G | -0.083 | 0.019 | 7.37E-06 | 0.017 | 0.044 | 0.70 | |
| *Ruminococcaceae (UCG014)* | rs439810 | G | C | -0.058 | 0.013 | 5.21E-06 | -0.011 | 0.031 | 0.73 | |
| *Ruminococcaceae (UCG014)* | rs62478832 | T | A | -0.058 | 0.013 | 6.64E-06 | 0.003 | 0.032 | 0.92 | |
| *Ruminococcaceae (UCG014)* | rs72809222 | T | C | 0.067 | 0.014 | 1.56E-06 | 0.015 | 0.033 | 0.64 | |
| *Ruminococcaceae (UCG014)* | rs73186226 | G | A | -0.099 | 0.022 | 4.57E-06 | -0.099 | 0.054 | 0.07 | |
| *Ruminococcaceae (UCG014)* | rs74060145 | C | G | -0.116 | 0.025 | 5.26E-06 | 0.103 | 0.062 | 0.10 | |
| *Ruminococcaceae (UCG014)* | rs7528298 | A | C | -0.097 | 0.022 | 9.62E-06 | 0.068 | 0.053 | 0.21 | |
| *Ruminococcaceae (UCG014)* | rs7655928 | C | A | -0.065 | 0.014 | 6.65E-06 | -0.071 | 0.035 | 0.04 | |
| *Ruminococcaceae (UCG014)* | rs77627087 | C | G | 0.068 | 0.015 | 6.76E-06 | 0.001 | 0.038 | 0.99 | |
| *Ruminococcaceae (UCG014)* | rs853612 | A | G | -0.053 | 0.012 | 9.62E-06 | -0.029 | 0.029 | 0.32 | |
| *Ruminococcaceae (UCG014)* | rs995642 | C | T | 0.060 | 0.013 | 2.03E-06 | -0.011 | 0.031 | 0.74 | |
| *Ruminococcus (gauvreauii group)* | rs10931481 | G | A | 0.061 | 0.013 | 2.93E-06 | -0.147 | 0.031 | 0.00 | |
| *Ruminococcus (gauvreauii group)* | rs11750752 | C | T | 0.071 | 0.016 | 5.64E-06 | 0.011 | 0.036 | 0.77 | |
| *Ruminococcus (gauvreauii group)* | rs12079579 | A | G | 0.096 | 0.021 | 7.61E-06 | 0.032 | 0.051 | 0.53 | |
| *Ruminococcus (gauvreauii group)* | rs12539819 | C | T | 0.111 | 0.024 | 4.28E-06 | -0.106 | 0.064 | 0.10 | |
| *Ruminococcus (gauvreauii group)* | rs1391597 | C | T | 0.059 | 0.012 | 2.28E-06 | -0.015 | 0.031 | 0.63 | |
| *Ruminococcus (gauvreauii group)* | rs1510207 | C | G | 0.067 | 0.015 | 7.81E-06 | 0.008 | 0.035 | 0.81 | |
| *Ruminococcus (gauvreauii group)* | rs2047242 | A | G | -0.068 | 0.013 | 4.31E-07 | 0.056 | 0.032 | 0.08 | |
| *Ruminococcus (gauvreauii group)* | rs2105937 | A | G | 0.058 | 0.013 | 5.63E-06 | 0.040 | 0.030 | 0.19 | |
| *Ruminococcus (gauvreauii group)* | rs2166943 | A | C | 0.057 | 0.012 | 4.41E-06 | 0.055 | 0.029 | 0.06 | |
| *Ruminococcus (gauvreauii group)* | rs289410 | G | A | -0.065 | 0.014 | 2.50E-06 | 0.003 | 0.033 | 0.93 | |
| *Ruminococcus (gauvreauii group)* | rs431418 | A | G | -0.095 | 0.021 | 6.55E-06 | 0.038 | 0.050 | 0.45 | |
| *Ruminococcus (gauvreauii group)* | rs71386687 | T | G | 0.121 | 0.024 | 3.92E-07 | 0.025 | 0.062 | 0.69 | |
| *Ruminococcus (gauvreauii group)* | rs9870933 | A | G | 0.062 | 0.013 | 8.19E-07 | 0.021 | 0.030 | 0.48 | |
| *Ruminococcus (gnavus group)* | rs11597105 | A | G | 0.115 | 0.025 | 4.76E-06 | 0.001 | 0.042 | 0.98 | |
| *Ruminococcus (gnavus group)* | rs12136548 | C | T | 0.090 | 0.020 | 4.42E-06 | -0.055 | 0.031 | 0.08 | |
| *Ruminococcus (gnavus group)* | rs12989336 | G | A | -0.085 | 0.019 | 6.60E-06 | 0.017 | 0.031 | 0.58 | |
| *Ruminococcus (gnavus group)* | rs13163520 | G | A | -0.127 | 0.023 | 5.15E-08 | 0.022 | 0.039 | 0.58 | |
| *Ruminococcus (gnavus group)* | rs1916786 | C | T | -0.083 | 0.019 | 7.91E-06 | 0.020 | 0.034 | 0.56 | |
| *Ruminococcus (gnavus group)* | rs2909242 | C | A | -0.091 | 0.018 | 7.10E-07 | 0.024 | 0.031 | 0.43 | |
| *Ruminococcus (gnavus group)* | rs2975106 | T | C | -0.138 | 0.031 | 7.82E-06 | 0.001 | 0.056 | 0.98 | |
| *Ruminococcus (gnavus group)* | rs3124783 | A | G | -0.116 | 0.025 | 3.22E-06 | -0.018 | 0.043 | 0.68 | |
| *Ruminococcus (gnavus group)* | rs4388134 | C | T | -0.090 | 0.020 | 8.74E-06 | 0.038 | 0.035 | 0.28 | |
| *Ruminococcus (gnavus group)* | rs62167033 | T | C | 0.185 | 0.040 | 2.93E-06 | -0.032 | 0.068 | 0.64 | |
| *Ruminococcus (gnavus group)* | rs78399089 | T | C | 0.144 | 0.033 | 9.76E-06 | 0.096 | 0.053 | 0.07 | |
| *Ruminococcus (gnavus group)* | rs934940 | A | C | -0.105 | 0.023 | 4.75E-06 | -0.075 | 0.040 | 0.06 | |
| *Ruminococcus (gnavus group)* | rs9872758 | T | C | 0.085 | 0.018 | 1.53E-06 | 0.025 | 0.030 | 0.40 | |
| *Ruminococcus (torques group)* | rs10967781 | C | A | 0.051 | 0.011 | 7.37E-06 | 0.021 | 0.031 | 0.49 | |
| *Ruminococcus (torques group)* | rs1475330 | T | C | 0.052 | 0.012 | 9.47E-06 | 0.035 | 0.033 | 0.29 | |
| *Ruminococcus (torques group)* | rs158487 | A | G | 0.053 | 0.012 | 4.86E-06 | 0.005 | 0.033 | 0.87 | |
| *Ruminococcus (torques group)* | rs1972694 | T | A | -0.061 | 0.014 | 7.75E-06 | -0.054 | 0.042 | 0.20 | |
| *Ruminococcus (torques group)* | rs35866622 | T | C | -0.061 | 0.011 | 2.23E-08 | -0.041 | 0.030 | 0.17 | |
| *Ruminococcus (torques group)* | rs4073731 | T | C | 0.065 | 0.014 | 4.56E-06 | -0.023 | 0.040 | 0.56 | |
| *Ruminococcus (torques group)* | rs60603763 | C | G | 0.124 | 0.028 | 6.29E-06 | -0.095 | 0.065 | 0.14 | |
| *Ruminococcus (torques group)* | rs62263521 | C | T | 0.050 | 0.011 | 7.68E-06 | -0.060 | 0.032 | 0.06 | |
| *Ruminococcus (torques group)* | rs7042929 | A | C | 0.102 | 0.022 | 3.01E-06 | -0.044 | 0.062 | 0.48 | |
| *Ruminococcus (torques group)* | rs73130967 | A | T | 0.077 | 0.017 | 4.69E-06 | 0.025 | 0.043 | 0.56 | |
| *Ruminococcus (torques group)* | rs77034621 | T | G | -0.152 | 0.034 | 6.42E-06 | 0.046 | 0.063 | 0.47 | |
| *Ruminococcus (torques group)* | rs773123 | T | A | 0.082 | 0.017 | 2.14E-06 | 0.107 | 0.045 | 0.02 | |
| *Ruminococcus (torques group)* | rs8080469 | G | A | 0.049 | 0.011 | 4.55E-06 | -0.018 | 0.029 | 0.55 | |
| *Ruminococcus (torques group)* | rs8141465 | A | G | 0.048 | 0.011 | 7.65E-06 | -0.028 | 0.030 | 0.35 | |
| *Ruminococcus 1* | rs10769159 | G | C | -0.064 | 0.011 | 5.97E-09 | -0.004 | 0.029 | 0.88 | |
| *Ruminococcus 1* | rs11783695 | G | T | -0.073 | 0.016 | 5.40E-06 | 0.012 | 0.043 | 0.78 | |
| *Ruminococcus 1* | rs13219502 | C | T | 0.076 | 0.017 | 7.98E-06 | 0.000 | 0.050 | 0.99 | |
| *Ruminococcus 1* | rs17781867 | C | T | 0.100 | 0.021 | 2.36E-06 | -0.159 | 0.063 | 0.01 | |
| *Ruminococcus 1* | rs3819978 | C | T | -0.115 | 0.026 | 9.77E-06 | -0.130 | 0.073 | 0.07 | |
| *Ruminococcus 1* | rs4849717 | T | A | 0.133 | 0.030 | 9.50E-06 | 0.105 | 0.070 | 0.13 | |
| *Ruminococcus 1* | rs6105066 | T | C | -0.061 | 0.013 | 6.13E-06 | 0.043 | 0.037 | 0.25 | |
| *Ruminococcus 1* | rs6493760 | C | T | 0.054 | 0.012 | 3.86E-06 | -0.016 | 0.032 | 0.62 | |
| *Ruminococcus 1* | rs6750529 | C | T | 0.057 | 0.013 | 7.79E-06 | -0.047 | 0.033 | 0.15 | |
| *Ruminococcus 1* | rs7117576 | A | G | 0.083 | 0.017 | 1.21E-06 | -0.050 | 0.049 | 0.31 | |
| *Ruminococcus 1* | rs7583465 | C | T | 0.053 | 0.011 | 2.80E-06 | -0.014 | 0.029 | 0.63 | |
| *Ruminococcus 1* | rs78572139 | G | A | 0.125 | 0.028 | 7.64E-06 | -0.079 | 0.064 | 0.22 | |
| *Ruminococcus 1* | rs78613526 | G | A | 0.167 | 0.037 | 5.21E-06 | 0.086 | 0.079 | 0.28 | |
| *Ruminococcus 1* | rs9527268 | A | T | -0.109 | 0.024 | 5.15E-06 | 0.049 | 0.062 | 0.43 | |
| *Ruminococcus 2* | rs10199274 | G | C | 0.066 | 0.014 | 1.94E-06 | 0.059 | 0.038 | 0.12 | |
| *Ruminococcus 2* | rs12406309 | A | C | -0.063 | 0.014 | 8.93E-06 | 0.015 | 0.038 | 0.70 | |
| *Ruminococcus 2* | rs1819812 | G | T | 0.084 | 0.018 | 5.22E-06 | -0.032 | 0.049 | 0.52 | |
| *Ruminococcus 2* | rs2368224 | T | G | 0.200 | 0.044 | 5.28E-06 | -0.057 | 0.082 | 0.49 | |
| *Ruminococcus 2* | rs2846589 | G | T | 0.052 | 0.012 | 7.26E-06 | -0.025 | 0.031 | 0.41 | |
| *Ruminococcus 2* | rs2997412 | A | G | -0.057 | 0.012 | 3.37E-06 | -0.020 | 0.031 | 0.53 | |
| *Ruminococcus 2* | rs4400279 | A | G | 0.055 | 0.012 | 5.42E-06 | 0.061 | 0.030 | 0.04 | |
| *Ruminococcus 2* | rs4799823 | C | T | 0.084 | 0.018 | 4.38E-06 | 0.064 | 0.050 | 0.20 | |
| *Ruminococcus 2* | rs55707116 | C | A | 0.087 | 0.019 | 4.74E-06 | 0.012 | 0.049 | 0.81 | |
| *Ruminococcus 2* | rs58681734 | A | G | 0.072 | 0.016 | 6.73E-06 | 0.050 | 0.041 | 0.23 | |
| *Ruminococcus 2* | rs61791565 | T | C | -0.052 | 0.012 | 7.83E-06 | 0.026 | 0.030 | 0.40 | |
| *Ruminococcus 2* | rs75140805 | T | G | 0.084 | 0.018 | 2.11E-06 | 0.130 | 0.053 | 0.01 | |
| *Ruminococcus 2* | rs7635831 | G | A | 0.062 | 0.013 | 1.65E-06 | 0.031 | 0.039 | 0.42 | |
| *Ruminococcus 2* | rs78120384 | A | G | -0.193 | 0.039 | 8.73E-07 | -0.105 | 0.079 | 0.18 | |
| *Sellimonas* | rs113379006 | T | C | -0.163 | 0.036 | 5.15E-06 | 0.065 | 0.041 | 0.11 | |
| *Sellimonas* | rs11711574 | T | C | 0.154 | 0.035 | 9.04E-06 | -0.031 | 0.041 | 0.44 | |
| *Sellimonas* | rs13417181 | T | C | 0.167 | 0.034 | 8.09E-07 | -0.007 | 0.049 | 0.89 | |
| *Sellimonas* | rs2016057 | A | C | -0.126 | 0.026 | 8.92E-07 | -0.020 | 0.031 | 0.53 | |
| *Sellimonas* | rs2187447 | A | C | 0.243 | 0.053 | 3.96E-06 | 0.031 | 0.061 | 0.61 | |
| *Sellimonas* | rs2371572 | A | C | 0.127 | 0.025 | 3.85E-07 | -0.014 | 0.029 | 0.64 | |
| *Sellimonas* | rs41816 | A | G | 0.132 | 0.029 | 5.58E-06 | -0.027 | 0.035 | 0.44 | |
| *Sellimonas* | rs4600608 | A | G | -0.137 | 0.030 | 5.47E-06 | -0.059 | 0.037 | 0.11 | |
| *Sellimonas* | rs553697 | T | C | -0.154 | 0.034 | 5.77E-06 | -0.003 | 0.041 | 0.93 | |
| *Sellimonas* | rs56203279 | T | C | -0.124 | 0.027 | 4.04E-06 | 0.023 | 0.032 | 0.48 | |
| *Sellimonas* | rs72553859 | G | C | -0.150 | 0.033 | 6.09E-06 | 0.024 | 0.039 | 0.54 | |
| *Sellimonas* | rs7968030 | A | T | -0.127 | 0.028 | 5.91E-06 | 0.003 | 0.034 | 0.93 | |
| *Senegalimassilia* | rs10036909 | C | T | 0.186 | 0.040 | 3.70E-06 | 0.023 | 0.057 | 0.69 | |
| *Senegalimassilia* | rs10221578 | G | A | 0.078 | 0.017 | 5.72E-06 | 0.030 | 0.029 | 0.30 | |
| *Senegalimassilia* | rs11787826 | C | A | 0.081 | 0.017 | 2.02E-06 | -0.011 | 0.029 | 0.70 | |
| *Senegalimassilia* | rs1514381 | G | C | -0.170 | 0.038 | 8.68E-06 | -0.006 | 0.057 | 0.91 | |
| *Senegalimassilia* | rs1990708 | A | C | -0.110 | 0.025 | 9.69E-06 | 0.011 | 0.043 | 0.80 | |
| *Senegalimassilia* | rs2017373 | C | T | 0.078 | 0.018 | 9.71E-06 | 0.029 | 0.031 | 0.36 | |
| *Senegalimassilia* | rs57512504 | T | A | 0.082 | 0.017 | 1.96E-06 | -0.053 | 0.030 | 0.07 | |
| *Senegalimassilia* | rs7225245 | G | A | 0.079 | 0.017 | 3.39E-06 | -0.002 | 0.036 | 0.96 | |
| *Senegalimassilia* | rs72887800 | T | A | -0.082 | 0.018 | 2.86E-06 | 0.073 | 0.031 | 0.02 | |
| *Slackia* | rs1006200 | G | A | -0.090 | 0.020 | 9.46E-06 | -0.020 | 0.034 | 0.56 | |
| *Slackia* | rs10409783 | A | G | 0.095 | 0.021 | 6.76E-06 | -0.011 | 0.040 | 0.78 | |
| *Slackia* | rs112764253 | T | A | 0.195 | 0.041 | 2.24E-06 | -0.102 | 0.067 | 0.12 | |
| *Slackia* | rs12440440 | A | G | 0.090 | 0.019 | 2.22E-06 | 0.042 | 0.031 | 0.17 | |
| *Slackia* | rs13339230 | C | G | 0.147 | 0.033 | 8.68E-06 | 0.015 | 0.055 | 0.79 | |
| *Slackia* | rs16894137 | C | T | -0.123 | 0.026 | 3.04E-06 | 0.048 | 0.043 | 0.27 | |
| *Slackia* | rs35156985 | T | C | -0.156 | 0.035 | 7.70E-06 | -0.122 | 0.063 | 0.05 | |
| *Slackia* | rs4492265 | A | G | -0.091 | 0.019 | 2.29E-06 | 0.056 | 0.031 | 0.07 | |
| *Slackia* | rs58767323 | G | C | -0.103 | 0.023 | 5.94E-06 | 0.005 | 0.038 | 0.90 | |
| *Slackia* | rs6133228 | A | G | -0.082 | 0.019 | 9.72E-06 | 0.028 | 0.031 | 0.37 | |
| *Slackia* | rs6544974 | G | C | 0.081 | 0.018 | 9.89E-06 | -0.002 | 0.032 | 0.95 | |
| *Slackia* | rs7710333 | C | T | 0.144 | 0.032 | 6.53E-06 | 0.023 | 0.051 | 0.65 | |
| *Slackia* | rs8901 | C | T | 0.093 | 0.019 | 5.65E-07 | -0.009 | 0.030 | 0.77 | |
| *Streptococcus* | rs10028567 | C | T | -0.092 | 0.019 | 1.58E-06 | -0.083 | 0.050 | 0.05 | |
| *Streptococcus* | rs10448310 | A | G | -0.052 | 0.011 | 3.28E-06 | -0.014 | 0.029 | 0.03 | |
| *Streptococcus* | rs11110281 | T | C | -0.138 | 0.023 | 1.47E-09 | 0.084 | 0.060 | 0.06 | |
| *Streptococcus* | rs11720390 | G | A | 0.107 | 0.023 | 2.71E-06 | -0.085 | 0.058 | 0.06 | |
| *Streptococcus* | rs17708276 | A | G | -0.079 | 0.017 | 3.27E-06 | 0.039 | 0.043 | 0.04 | |
| *Streptococcus* | rs1918540 | G | A | 0.060 | 0.013 | 3.26E-06 | 0.042 | 0.034 | 0.03 | |
| *Streptococcus* | rs2561144 | G | T | -0.076 | 0.017 | 9.14E-06 | 0.099 | 0.047 | 0.05 | |
| *Streptococcus* | rs395407 | G | C | -0.079 | 0.017 | 5.02E-06 | 0.075 | 0.051 | 0.05 | |
| *Streptococcus* | rs60486012 | G | A | -0.091 | 0.020 | 5.52E-06 | 0.071 | 0.057 | 0.06 | |
| *Streptococcus* | rs6563952 | G | C | 0.083 | 0.018 | 4.32E-06 | -0.084 | 0.051 | 0.05 | |
| *Streptococcus* | rs6806351 | T | C | -0.063 | 0.014 | 3.51E-06 | 0.036 | 0.035 | 0.03 | |
| *Streptococcus* | rs71481756 | T | G | 0.093 | 0.021 | 7.56E-06 | 0.046 | 0.061 | 0.06 | |
| *Streptococcus* | rs72739637 | A | G | 0.096 | 0.019 | 6.75E-07 | 0.034 | 0.048 | 0.05 | |
| *Streptococcus* | rs7916711 | A | G | 0.103 | 0.022 | 2.21E-06 | 0.028 | 0.062 | 0.06 | |
| *Streptococcus* | rs957755 | T | G | -0.070 | 0.014 | 1.22E-06 | 0.044 | 0.037 | 0.04 | |
| *Streptococcus* | rs9895557 | T | C | -0.051 | 0.011 | 4.29E-06 | -0.003 | 0.029 | 0.03 | |
| *Streptococcus* | rs9903102 | C | A | -0.071 | 0.016 | 4.90E-06 | -0.022 | 0.043 | 0.04 | |
| *Subdoligranulum* | rs10065321 | T | C | -0.051 | 0.011 | 2.10E-06 | -0.004 | 0.030 | 0.89 | |
| *Subdoligranulum* | rs11841695 | C | T | 0.079 | 0.018 | 9.70E-06 | -0.125 | 0.049 | 0.01 | |
| *Subdoligranulum* | rs12638227 | G | C | -0.056 | 0.011 | 2.84E-07 | -0.011 | 0.032 | 0.74 | |
| *Subdoligranulum* | rs1667315 | G | A | 0.049 | 0.011 | 6.37E-06 | -0.016 | 0.030 | 0.60 | |
| *Subdoligranulum* | rs16962433 | A | T | 0.086 | 0.019 | 6.07E-06 | -0.119 | 0.049 | 0.02 | |
| *Subdoligranulum* | rs2114677 | C | T | -0.104 | 0.023 | 6.39E-06 | -0.117 | 0.064 | 0.07 | |
| *Subdoligranulum* | rs2171249 | C | T | 0.107 | 0.023 | 4.71E-06 | -0.053 | 0.063 | 0.40 | |
| *Subdoligranulum* | rs35940633 | G | A | -0.051 | 0.011 | 3.40E-06 | -0.046 | 0.030 | 0.13 | |
| *Subdoligranulum* | rs3761728 | T | G | -0.054 | 0.012 | 4.83E-06 | 0.047 | 0.032 | 0.14 | |
| *Subdoligranulum* | rs4347804 | A | G | 0.166 | 0.036 | 3.40E-06 | 0.090 | 0.073 | 0.22 | |
| *Subdoligranulum* | rs56273211 | T | C | -0.048 | 0.011 | 9.84E-06 | 0.024 | 0.029 | 0.42 | |
| *Subdoligranulum* | rs6555306 | T | C | -0.074 | 0.016 | 1.89E-06 | -0.020 | 0.044 | 0.64 | |
| *Subdoligranulum* | rs75158211 | T | C | -0.072 | 0.016 | 5.61E-06 | -0.076 | 0.045 | 0.09 | |
| *Subdoligranulum* | rs76528319 | G | T | -0.143 | 0.031 | 3.99E-06 | 0.029 | 0.074 | 0.69 | |
| *Subdoligranulum* | rs76664262 | T | A | 0.083 | 0.019 | 6.80E-06 | -0.009 | 0.051 | 0.87 | |
| *Sutterella* | rs1145877 | A | G | -0.074 | 0.016 | 5.94E-06 | 0.007 | 0.042 | 0.87 | |
| *Sutterella* | rs11591622 | T | G | -0.069 | 0.015 | 5.43E-06 | -0.018 | 0.034 | 0.59 | |
| *Sutterella* | rs13173038 | A | G | -0.072 | 0.015 | 2.18E-06 | -0.026 | 0.036 | 0.47 | |
| *Sutterella* | rs143438747 | T | C | -0.146 | 0.031 | 2.02E-06 | 0.023 | 0.066 | 0.73 | |
| *Sutterella* | rs2050185 | G | A | 0.058 | 0.013 | 7.95E-06 | -0.036 | 0.030 | 0.24 | |
| *Sutterella* | rs2321387 | G | A | -0.059 | 0.012 | 1.92E-06 | -0.052 | 0.030 | 0.08 | |
| *Sutterella* | rs2613606 | C | T | -0.056 | 0.012 | 7.26E-06 | 0.019 | 0.029 | 0.52 | |
| *Sutterella* | rs35716880 | T | C | -0.106 | 0.024 | 9.41E-06 | -0.056 | 0.059 | 0.34 | |
| *Sutterella* | rs607327 | C | T | 0.058 | 0.013 | 7.42E-06 | 0.031 | 0.030 | 0.30 | |
| *Sutterella* | rs62501473 | G | A | 0.069 | 0.015 | 3.38E-06 | -0.052 | 0.037 | 0.16 | |
| *Sutterella* | rs7499539 | A | G | 0.062 | 0.013 | 2.43E-06 | 0.014 | 0.031 | 0.65 | |
| *Sutterella* | rs7638039 | T | C | 0.065 | 0.014 | 7.22E-06 | -0.067 | 0.034 | 0.05 | |
| *Sutterella* | rs9350083 | T | G | -0.059 | 0.013 | 9.50E-06 | 0.048 | 0.031 | 0.12 | |
| *Terrisporobacter* | rs1883097 | C | T | 0.226 | 0.045 | 6.37E-07 | -0.039 | 0.063 | 0.54 | |
| *Terrisporobacter* | rs2569953 | A | C | -0.078 | 0.017 | 8.95E-06 | -0.063 | 0.029 | 0.03 | |
| *Terrisporobacter* | rs2872237 | C | A | -0.081 | 0.018 | 3.67E-06 | 0.007 | 0.030 | 0.81 | |
| *Terrisporobacter* | rs58405430 | G | T | 0.135 | 0.030 | 7.83E-06 | -0.033 | 0.047 | 0.49 | |
| *Terrisporobacter* | rs7034891 | G | C | -0.080 | 0.017 | 4.25E-06 | 0.041 | 0.029 | 0.16 | |
| *Terrisporobacter* | rs7184125 | T | C | 0.091 | 0.021 | 8.96E-06 | -0.017 | 0.037 | 0.64 | |
| *Terrisporobacter* | rs7812756 | C | T | 0.120 | 0.027 | 8.56E-06 | -0.009 | 0.056 | 0.87 | |
| *Turicibacter* | rs11054680 | T | C | -0.105 | 0.023 | 3.94E-06 | -0.010 | 0.046 | 0.84 | |
| *Turicibacter* | rs114145484 | G | C | -0.122 | 0.027 | 6.32E-06 | 0.010 | 0.053 | 0.85 | |
| *Turicibacter* | rs11649454 | G | C | 0.095 | 0.020 | 2.95E-06 | -0.011 | 0.042 | 0.80 | |
| *Turicibacter* | rs11666533 | C | T | -0.112 | 0.025 | 6.93E-06 | -0.036 | 0.055 | 0.52 | |
| *Turicibacter* | rs11929602 | C | T | -0.084 | 0.019 | 8.41E-06 | 0.017 | 0.039 | 0.66 | |
| *Turicibacter* | rs12603364 | T | C | 0.111 | 0.023 | 8.92E-07 | 0.013 | 0.046 | 0.77 | |
| *Turicibacter* | rs149744580 | A | G | 0.170 | 0.032 | 7.25E-08 | -0.034 | 0.065 | 0.60 | |
| *Turicibacter* | rs2221441 | G | C | 0.071 | 0.015 | 3.66E-06 | -0.016 | 0.032 | 0.61 | |
| *Turicibacter* | rs2834977 | T | C | -0.096 | 0.021 | 4.03E-06 | -0.093 | 0.041 | 0.02 | |
| *Turicibacter* | rs2952020 | G | A | -0.076 | 0.017 | 4.67E-06 | -0.049 | 0.034 | 0.14 | |
| *Turicibacter* | rs4247078 | C | G | 0.071 | 0.016 | 4.73E-06 | -0.045 | 0.030 | 0.14 | |
| *Turicibacter* | rs4869133 | G | A | 0.131 | 0.027 | 1.41E-06 | 0.042 | 0.058 | 0.47 | |
| *Turicibacter* | rs55756211 | T | C | -0.115 | 0.024 | 1.73E-06 | 0.027 | 0.049 | 0.58 | |
| *Turicibacter* | rs61265175 | G | C | -0.086 | 0.019 | 3.81E-06 | -0.024 | 0.036 | 0.51 | |
| *Turicibacter* | rs7199484 | G | A | -0.073 | 0.016 | 4.96E-06 | 0.012 | 0.032 | 0.70 | |
| *Turicibacter* | rs73174561 | A | C | 0.214 | 0.048 | 9.38E-06 | 0.034 | 0.070 | 0.63 | |
| *Turicibacter* | rs9508664 | A | G | 0.086 | 0.019 | 8.85E-06 | 0.014 | 0.039 | 0.72 | |
| *Tyzzerella 3* | rs10463669 | C | A | -0.090 | 0.020 | 7.15E-06 | -0.058 | 0.031 | 0.06 | |
| *Tyzzerella 3* | rs10898797 | C | T | 0.122 | 0.027 | 8.38E-06 | 0.038 | 0.040 | 0.34 | |
| *Tyzzerella 3* | rs112102233 | A | G | -0.216 | 0.048 | 5.89E-06 | 0.100 | 0.067 | 0.13 | |
| *Tyzzerella 3* | rs1232220 | G | T | -0.144 | 0.032 | 6.18E-06 | 0.017 | 0.050 | 0.73 | |
| *Tyzzerella 3* | rs17706273 | T | C | -0.140 | 0.027 | 3.23E-07 | 0.004 | 0.043 | 0.93 | |
| *Tyzzerella 3* | rs17809157 | A | T | -0.164 | 0.034 | 1.12E-06 | 0.015 | 0.056 | 0.79 | |
| *Tyzzerella 3* | rs191093 | G | A | 0.159 | 0.035 | 6.78E-06 | -0.004 | 0.056 | 0.94 | |
| *Tyzzerella 3* | rs2144925 | A | G | -0.125 | 0.028 | 9.56E-06 | -0.034 | 0.044 | 0.44 | |
| *Tyzzerella 3* | rs4904512 | T | C | -0.117 | 0.025 | 2.86E-06 | 0.034 | 0.037 | 0.36 | |
| *Tyzzerella 3* | rs55799124 | A | G | -0.114 | 0.024 | 1.65E-06 | -0.033 | 0.037 | 0.37 | |
| *Tyzzerella 3* | rs57025483 | C | A | -0.089 | 0.020 | 8.83E-06 | -0.089 | 0.031 | 0.00 | |
| *Tyzzerella 3* | rs5759580 | A | G | -0.096 | 0.022 | 8.03E-06 | -0.031 | 0.034 | 0.37 | |
| *Tyzzerella 3* | rs67476743 | T | G | 0.132 | 0.022 | 2.66E-09 | -0.001 | 0.034 | 0.97 | |
| *Tyzzerella 3* | rs6920448 | C | T | -0.141 | 0.031 | 3.86E-06 | 0.032 | 0.047 | 0.49 | |
| *Tyzzerella 3* | rs7019909 | T | C | 0.144 | 0.030 | 1.76E-06 | 0.004 | 0.049 | 0.93 | |
| *Tyzzerella 3* | rs7333521 | T | C | -0.207 | 0.045 | 4.82E-06 | -0.125 | 0.065 | 0.05 | |
| *Tyzzerella 3* | rs75091807 | G | T | -0.185 | 0.038 | 1.37E-06 | -0.031 | 0.057 | 0.58 | |
| *Tyzzerella 3* | rs7561370 | T | C | 0.131 | 0.029 | 4.48E-06 | -0.016 | 0.043 | 0.72 | |
| *Veillonella* | rs10981640 | A | C | 0.082 | 0.019 | 9.30E-06 | 0.002 | 0.038 | 0.96 | |
| *Veillonella* | rs11141494 | G | A | -0.078 | 0.017 | 7.05E-06 | 0.018 | 0.036 | 0.61 | |
| *Veillonella* | rs11614532 | G | C | 0.074 | 0.017 | 6.59E-06 | -0.020 | 0.032 | 0.55 | |
| *Veillonella* | rs12679709 | C | G | -0.079 | 0.016 | 1.48E-06 | -0.043 | 0.032 | 0.18 | |
| *Veillonella* | rs147857104 | C | A | 0.191 | 0.043 | 7.97E-06 | -0.087 | 0.079 | 0.27 | |
| *Veillonella* | rs1882878 | A | G | -0.077 | 0.016 | 2.71E-06 | -0.009 | 0.033 | 0.78 | |
| *Veillonella* | rs2013594 | T | C | -0.072 | 0.016 | 3.40E-06 | -0.016 | 0.032 | 0.62 | |
| *Veillonella* | rs203802 | C | T | -0.066 | 0.015 | 9.84E-06 | 0.059 | 0.030 | 0.05 | |
| *Veillonella* | rs55807413 | A | G | 0.107 | 0.024 | 6.28E-06 | 0.001 | 0.046 | 0.98 | |
| *Veillonella* | rs62376424 | C | T | -0.076 | 0.016 | 3.13E-06 | 0.013 | 0.031 | 0.66 | |
| *Veillonella* | rs6656807 | A | G | 0.070 | 0.015 | 4.95E-06 | 0.007 | 0.035 | 0.84 | |
| *Veillonella* | rs742016 | A | G | -0.069 | 0.015 | 4.27E-06 | -0.003 | 0.030 | 0.93 | |
| *Veillonella* | rs7645873 | A | T | 0.076 | 0.016 | 3.55E-06 | -0.023 | 0.032 | 0.48 | |
| *Victivallis* | rs11899949 | G | A | 0.131 | 0.028 | 2.28E-06 | 0.004 | 0.034 | 0.91 | |
| *Victivallis* | rs12512543 | A | C | -0.178 | 0.037 | 1.98E-06 | 0.054 | 0.045 | 0.23 | |
| *Victivallis* | rs173120 | T | C | 0.134 | 0.029 | 3.99E-06 | 0.047 | 0.034 | 0.16 | |
| *Victivallis* | rs1882775 | A | G | -0.138 | 0.031 | 9.81E-06 | 0.031 | 0.037 | 0.41 | |
| *Victivallis* | rs2546432 | T | C | -0.111 | 0.025 | 9.07E-06 | -0.012 | 0.029 | 0.69 | |
| *Victivallis* | rs4764863 | G | A | 0.122 | 0.025 | 7.80E-07 | -0.026 | 0.030 | 0.39 | |
| *Victivallis* | rs4895919 | T | C | -0.117 | 0.025 | 2.32E-06 | 0.003 | 0.030 | 0.91 | |
| *Victivallis* | rs56349194 | A | G | -0.159 | 0.032 | 4.95E-07 | 0.096 | 0.038 | 0.01 | |
| *Victivallis* | rs592514 | T | A | -0.181 | 0.039 | 3.61E-06 | 0.060 | 0.044 | 0.18 | |
| *Victivallis* | rs6445926 | G | C | 0.117 | 0.025 | 2.79E-06 | -0.022 | 0.030 | 0.46 | |
| *Victivallis* | rs911666 | T | C | -0.119 | 0.026 | 6.60E-06 | -0.025 | 0.031 | 0.42 | |
| MR, Mendelian randomization; JIA, Juvenile idiopathic arthritis; SNP, single nucleotide polymorphism; SE, standard error. | | | | | | | | | |  |
